# Genetic analysis of the barley variegation mutant, *grandpa1.a*

**DOI:** 10.1186/s12870-021-02915-9

**Published:** 2021-03-13

**Authors:** Shengming Yang, Megan Overlander, Jason Fiedler

**Affiliations:** 1 USDA-ARS Cereals Research Unit, Edward T. Schafer Agriculture Research Center, Fargo, ND 58102 USA; 2 Department of Plant Sciences, North Dakota State University, Fargo, ND 58102 USA; 3 Department of Plant Pathology, North Dakota State University, Fargo, ND 58102 USA

**Keywords:** Gpa1, Chloroplast biogenesis, Genetic mapping, Barley

## Abstract

**Background:**

Providing the photosynthesis factory for plants, chloroplasts are critical for crop biomass and economic yield. However, chloroplast development is a complicated process, coordinated by the cross-communication between the nucleus and plastids, and the underlying biogenesis mechanism has not been fully revealed. Variegation mutants have provided ideal models to identify genes or factors involved in chloroplast development. Well-developed chloroplasts are present in the green tissue areas, while the white areas contain undifferentiated plastids that are deficient in chlorophyll. Unlike albino plants, variegation mutants survive to maturity and enable investigation into the signaling pathways underlying chloroplast biogenesis. The allelic variegated mutants in barley, *grandpa 1* (*gpa1*), have long been identified but have not been genetically characterized.

**Results:**

We characterized and genetically analyzed the *grandpa1.a* (*gpa1.a*) mutant. The chloroplast ultrastructure was evaluated using transmission electron microscopy (TEM), and it was confirmed that chloroplast biogenesis was disrupted in the white sections of *gpa1.a*. To determine the precise position of *Gpa1*, a high-resolution genetic map was constructed. Segregating individuals were genotyped with the barley 50 k iSelect SNP Array, and the linked SNPs were converted to PCR-based markers for genetic mapping. The *Gpa1* gene was mapped to chromosome 2H within a gene cluster functionally related to photosynthesis or chloroplast differentiation. In the variegated *gpa1.a* mutant, we identified a large deletion in this gene cluster that eliminates a putative plastid terminal oxidase (PTOX).

**Conclusions:**

Here we characterized and genetically mapped the *gpa1.a* mutation causing a variegation phenotype in barley. The PTOX-encoding gene in the delimited region is a promising candidate for *Gpa1*. Therefore, the present study provides a foundation for the cloning of *Gpa1*, which will elevate our understanding of the molecular mechanisms underlying chloroplast biogenesis, particularly in monocot plants.

**Supplementary Information:**

The online version contains supplementary material available at 10.1186/s12870-021-02915-9.

## Background

As the characteristic organelle in plant cells, the chloroplast is critical for plant photosynthesis and has a significant impact on biomass and economic yield. An increasing number of studies have revealed that chloroplasts also make important contributions to plant immunity through the synthesis of secondary metabolites and defense phytohormones, such as reactive oxygen species, nitric oxide, jasmonic acid, and salicylic acid [1, 2]. To restrict pathogen infection, chloroplasts can navigate to the penetration site and directly suppress host cell invasion [3]. Therefore, the understanding of chloroplast biogenesis is necessary to meet the increasing food demand under rising population pressure.

Chloroplasts are differentiated from undeveloped plastids, which contain undifferentiated vesicles and lack stacked thylakoids (grana), the mounting-platform for chlorophyll. Chloroplast biogenesis is highly complex, being orchestrated by anterograde (nucleus to chloroplast) and retrograde (chloroplast to nucleus) signaling [4, 5]. More than 95% of the ∼3000 proteins found in chloroplasts are encoded by nuclear genes and imported into chloroplast following synthesis in the cytosol, suggesting that chloroplast development is predominantly controlled by the nuclear genome [6]. Therefore, identification and functional characterization of such nuclear genes is important to understand the regulatory mechanisms underlying chloroplast biosynthesis. Variegation mutants have provided ideal models to identify genes or factors involved in chloroplast development [7]. Well-developed chloroplasts are present in the green tissue areas, while the white areas contain undifferentiated plastids that are deficient in chlorophyll. Unlike albino plants, variegation mutants survive to set seed and enable investigation of cross-communication between the nucleus and plastids.

Two representative *Arabidopsis* variegation mutants, *immutans* (*im*) and *variegated 2* (*var2*), have been characterized and provided fundamental perspectives to the understanding of chloroplast biogenesis in plants [8–12]. The *im* mutant is caused by loss-of-function of a nuclear-encoded plastid terminal oxidase (PTOX), normally present in the thylakoid membranes. This plastoquinol oxidase also has homology to the alternative oxidase (AOX) in mitochondria [8, 9]. The *VAR2* gene, also known as *Filamentous temperature-sensitive H2* (*FtsH2*), encodes a chloroplast-targeting ATP-dependent zinc metalloprotease homologous to the *E. coli* FtsH [13]. Both IM and VAR2 play roles in photoprotection and in regulation of redox state of the photosynthetic electron transport chain [8, 10, 14, 15]. Lack of these proteins resulted in photodamaged/photooxidized plastids under high light, particularly in the white sections. Moreover, FtsH-mediated proteolysis is involved in retrograde signaling activated by ROS [16].

The green sections containing competent chloroplasts in variegation mutants may indicate the existence of compensatory mechanisms to escape the defect of mutation. Suppressor screening in the *im* and *var2* backgrounds has identified a few second-site mutations restoring the variegation phenotype. Suppressors of *im* include a thylakoid membrane protein (Chlororespiratory reduction 2–2, Crr2–2) and a plant combinatorial and modular protein (PCMP) family member (Proton Gradient Regulation 5, PGR5) [17]. The PCMP family is closely related to pentatricopeptide repeat (PPR) proteins functioning in the editing and maturation of organellar RNA. Both Crr2–2 and PGR5 are also required for alternative electron transport pathways that alleviate photodamage during chloroplast biogenesis and photosynthesis [18]. Most of the identified *var2* suppressors are involved in chloroplast translation or rRNA processing and editing, such as a chloroplast-localized pseudouridine synthase (Suppressor of Variegation1, SVR1), a ClpR1 subunit of the chloroplast ClpP/R protease (SVR2), a chloroplast translation initiation factor 2 (Fu-gaeri1, FUG1), a chloroplast translation elongation factor EF-G (Snow Cotyledon 1, SCO1), and a PPR protein (SVR7) [19]. Although variegated mutants and suppressor screening enabled the cloning of many genes involved in chloroplast development, there are still major gaps in the knowledge of chloroplast biogenesis and the variegation mechanism.

Due to the difference in chloroplast development between monocotyledonous and dicotyledonous plants, variegation mutants of monocots exhibit striping phenotype with alternating white and green bands on the leaf. Barley (*Hordeum vulgare subsp. vulgare*) is the fourth most important cereal crop which is used as feed grain, human food, and raw material for the malting and brewing industry. It is also a valuable model monocot for plant genomics research. Chemical- and radiation-mediated mutagenesis has created abundant genetic material for barley improvement and genomic research (http://www.nordgen.org/). Introgression of characterized mutations from various sources into the common background, Bowman, has generated a series of near-isogenic lines (NILs) with single mutated alleles, providing a powerful tool for rapid gene identification [20]. Several variegated mutants have been identified but few have been genetically characterized [21], including the allelic mutants of *grandpa* (*gpa* or *gp*) identified in 1940s [22–24].

An allelism test and linkage study with morphological markers anchored the *Gpa* gene onto the long arm of chromosome 2 (2HL) [20, 25]. However, the genetic control of the *gpa* mutants has not been investigated. The *gpa1.a* allele was caused by a spontaneous mutation in cultivar Lyallpur (GSHO 519), and it was introduced into the Bowman background with introgression [20]. Using BW397, a NIL of Bowman carrying the *gpa1.a* mutation, we characterized and finely mapped the *Gpa1* gene in the present study. It was revealed that chloroplast biogenesis is defective at the white stripes in the mutant, and the *Gpa1* gene is located in a gene cluster functionally related to photosynthesis or chloroplast differentiation. One gene in the delimited region codes for a putative PTOX homologous to IM of *Arabidopsis*, and we found that a large deletion occurring to this gene totally disrupted its function in BW397. Therefore, this PTOX-encoding gene was considered as a promising candidate for *Gpa1*. The high-resolution genetic map provided here lays the foundation for the cloning of this gene, which will further our understanding of molecular mechanisms underlying chloroplast biosynthesis.

## Results

### Phenotype characterization of the *gpa1* mutant

**Fig. 1:**
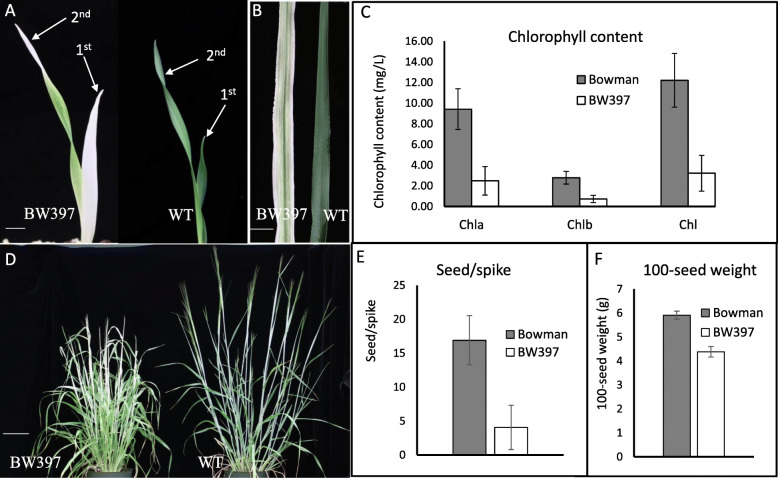
Phenotypic comparison between Bowman and BW397. Albino and striped leaves were observed on BW397 seedlings (**a** and **b**), chlorophyll-deficiency was confirmed by chemical analysis with four replications (**c**). With a smaller plant size, flowering BW397 produced albino or striped flag leaf, spike, and awns (**d**). Seed/spike (determined with 20 spikes, **e**) and seed weight (5 replications, **f**) of mutant was significantly reduced. Chl, chlorophyll; chla, chlorophyll a; chlb, chlorophyll b. Scale bars in A and B, 5 mm; Scale bar in D, 10 cm

Normally, the seedlings of *gpa1* have an albino first leaf and display chlorosis at the tip of the second leaf (Fig. 1a). A striped pattern then develops with expansion of the second leaf (Fig. 1a and b). Striping occasionally occurs on the third and subsequent leaves. Consistent with the visual difference, chemical analysis also indicated that levels of chlorophyll a, b and total in striped leaves of BW397 were significantly lower than that of WT Bowman plants (Fig. 1c). An adult BW397 plant produces albino or striped flag leaf, spike, awns and even anthers (Fig. 1d and Additional Fig. 1), and the mutant is much shorter than WT (Fig. 1d). The mutants display reduced fertility with an average of 4 seeds/spike compared to 18 seeds/spike in WT (Fig. 1e). Seed size of BW397, measured as 100-seed weight, is only 3/4 that of the WT’s (Fig. 1f). In addition to the striped leaf phenotype, the *gpa1* mutation causes a systemic effect on barley growth and development.

**Fig. 2:**
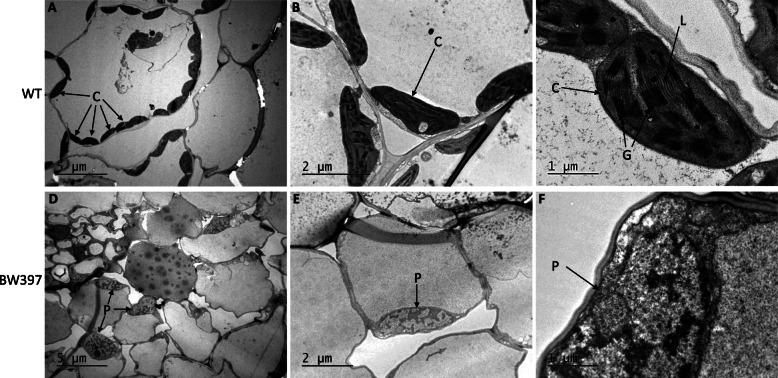
Chloroplast ultrastructure observed with a transmission electron microscope. Functional chloroplasts are well organized in the WT cells (A-C). Chloroplast are not developed in the white section of BW397 (**a**). Lamellae and stacked grana are missing in these undifferentiated plastids (**b**, **c**). C, chloroplast; G, grana; L, lamellae, P, plastid

To confirm the striped phenotype is associated with defects in chloroplast biogenesis, we compared the chloroplast ultrastructure of Bowman and BW397 using transmission electron microscopy (TEM) (Fig. 2). As expected, numerous normal chloroplasts with well-organized thylakoids are developed in the WT cells (Fig. 2a and b). Thylakoids are arranged in grana stacks, which are connected by stroma lamellae (Fig. 2c). On the contrary, BW397 cells in the albino sections contain few undeveloped plastids (Fig. 2d). Lamellae, acting like the skeleton in a normal chloroplast, is not well developed in the mutant (Fig. 2d, e, and f). Stacked grana and thylakoid membranes are also missing in these undifferentiated plastids (Fig. 2e and f). Therefore, chloroplast biogenesis in the white stripes of BW397 is abolished by the *gpa1* mutation.

### Genetic mapping of *Gpa1*

A total of 510 F_2_ plants derived from the cross between Bowman and BW397 were used for genetic mapping of *Gpa1*. Of those, 133 were striped, while the remaining 377 F_2_ plants exhibited normal green leaf color. The segregation ratio of striped/green fits 1:3 (χ^2^ = 0.316, df = 1, and *P* = 0.57), suggesting that the *gpa1* mutation is monofactorial recessive. As an immediate strategy for gene localization and marker discovery, SNP array analysis was used to genotype 48 F_2_ plants (24 striped and 24 green) together with the parental lines. As the *Gpa1* gene was anchored to 2H [20], we focused on the SNPs on 2H and identified 1013 polymorphic markers on this chromosome (Additional Table 1).

**Fig. 3:**
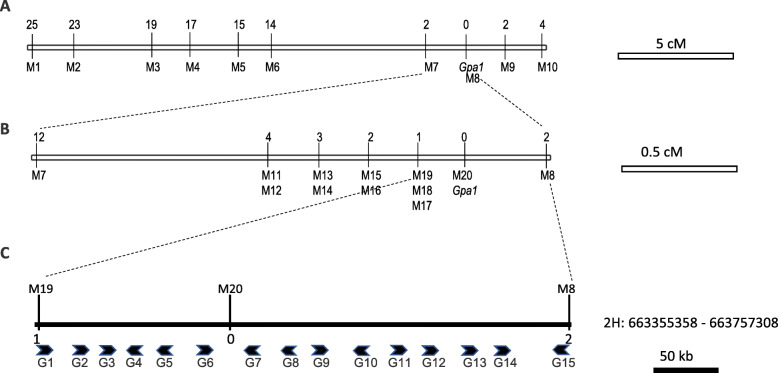
Genetic and physical mapping of the *Gpa1* locus. Genetic mapping was conducted sequentially with 104 (**a**) and 510 (**b**) F_2_ individuals. *Gpa1* is located on 2H, delimited to an ~ 0.7 cM region between markers M19 and M8 (**b**). Total of 15 protein-coding genes were identified in the *Gpa1* region spanning ~ 400 kb (**c**). Numbers above the linkage group indicate the number of recombination breakpoints separating the marker from *Gpa1*. The maps are drawn to scale. M, marker; G, gene

Initial genotype analysis of 48 F_2_ progeny revealed that the *Gpa1* gene was located in ~ 5.56 Mb region flanked by two array SNPs, JHI-Hv50k-2016-139,629 and JHI-Hv50k-2016-142,540 (Additional Table 1). To increase the resolution of mapping, we enriched this region with SSR markers previously co-located to the stripped phenotype [20], known SNPs on 2H consensus map [26], and specific 50 k markers within this region (Table 1). The analysis of an additional 104 F_2_ progeny genotyped with these markers further delimited the *Gpa1* gene between M4 and M6 (Fig. 3a). When we enlarged the segregating population to 510 F_2_ plants, the *Gpa1* region was narrowed down to a 410-kb region by M19 and M8 (Fig. 3b), where one co-segregating SNP, M20, was identified.

**Table 1:** Genetic markers used for mapping of the *Gpa1* gene

Marker name	SNP or ploymorphism source	Marker type	Forward primer 1	Forward primer 2	Reverse primer
**M1**	**Ebmac0415**	**SSR**	**GAAACCCATCATAGCAGC**		**AAACAGCAGCAAGAGGAG**
**M2**	**HVM54**	**SSR**	**AACCCAGTAACACCGTCCTG**		**AGTTCCCTGACCCGATGTC**
**M3**	**Gbm1437**	**SSR**	**ATTCCACTCGCCATTGTTTC**		**CACTCCGGATTGATGCTCTT**
**M4**	**GBM1498**	**SSR**	**TGCTCCAACCCAAAAGCTAC**		**GAAGACGACGAGCGGTACTC**
**M5**	**POPA1_10315**	**STARP**	**GCAACAGGAACCAGCTATGACGGGGAGGTCGGGGAAC**	**GACGCAAGTGAGCAGTATGACGGGGAGGTCGGGAAGG**	**CGTCCATCCCATTCCCCAAA**
**M6**	**POPA1_11380**	**STARP**	**GCAACAGGAACCAGCTATGACCTGTTTCTGTTTTATTTTTCTTCCG**	**GACGCAAGTGAGCAGTATGACCTGTTTCTGTTTTATTTTTCTCTCA**	**AGATCGGGGGAGTTGCACTA**
**M7**	**POPA1_10791**	**STARP**	**GCAACAGGAACCAGCTATGACGTGCCGCACACCATTG**	**GACGCAAGTGAGCAGTATGACGTGCCGCACACTGTTT**	**GACAACCCATCCAAGGCACT**
**M8**	**Bmag0749**	**SSR**	**CGGATTCTTGAGTAGTCTCTG**		**GATCTGTTTTTGTAGAACATGC**
**M9**	**POPA1_21453**	**STARP**	**GCAACAGGAACCAGCTATGACGTTGCTCACGGAAATTGG**	**GACGCAAGTGAGCAGTATGACGTTGCTCACGGAAACCGA**	**GAACCCCTGAATAGGTGGCA**
**M10**	**POPA1_10085**	**STARP**	**GCAACAGGAACCAGCTATGACCGCGGAAATCTTGTATTAACAAC**	**GACGCAAGTGAGCAGTATGACCGCGGAAATCTTGTATTAATCAT**	**AGTCTCGGCTATTCCCCACT**
**M11**	**JHI-Hv50k-2016-140,235**	**STARP**	**GCAACAGGAACCAGCTATGACCCTATGTAATTTCCAGACACATC**	**GACGCAAGTGAGCAGTATGACCCTATGTAATTTCCAGACAACTT**	**TCGCTGCATTTTGGAGACCT**
**M12**	**JHI-Hv50k-2016-140,239**	**STARP**	**GCAACAGGAACCAGCTATGACGTTTTCCTGAGAAATACCTCAC**	**GACGCAAGTGAGCAGTATGACGTTTTCCTGAGAAATACCCAAA**	**GTTGGCCTAGACGACGGTT**
**M13**	**JHI-Hv50k-2016-140,382**	**STARP**	**GCAACAGGAACCAGCTATGACAGGAAGACAGAGAGTTGACTTC**	**GACGCAAGTGAGCAGTATGACAGGAAGACAGAGAGTTGGTTTG**	**ATAAGCCCCACTTCCACCAG**
**M14**	**JHI-Hv50k-2016-140,496**	**STARP**	**GCAACAGGAACCAGCTATGACGTACTGCCGCGTGAGG**	**GACGCAAGTGAGCAGTATGACGTACTGCCGCGTAGGA**	**TGATGTACTCTCGGCAAGCG**
**M15**	**JHI-Hv50k-2016-140,683**	**STARP**	**GCAACAGGAACCAGCTATGACAGTCGATGTAATCAGCTCCC**	**GACGCAAGTGAGCAGTATGACAGTCGATGTAATCAGCCACA**	**ACAACATACGCAGCACCAAC**
**M16**	**JHI-Hv50k-2016-140,672**	**STARP**	**GCAACAGGAACCAGCTATGACTGCGCAATCACTTGCTTG**	**GACGCAAGTGAGCAGTATGACTGCGCAATCACTTGTTCA**	**ATGCCAGCAAGAGAGCATCA**
**M17**	**JHI-Hv50k-2016-140,582**	**STARP**	**GCAACAGGAACCAGCTATGACTTTTGCCTGAGTGTAATGCAC**	**GACGCAAGTGAGCAGTATGACTTTTGCCTGAGTGTAATGTCG**	**GAGCGATGGGACATGAGGAG**
**M18**	**JHI-Hv50k-2016-140,579**	**STARP**	**GCAACAGGAACCAGCTATGACATAGCAATAGCATAGCAGTGTG**	**GACGCAAGTGAGCAGTATGACATAGCAATAGCATAGCAGTACA**	**ACCAGGTTTTGCCTGAGTGT**
**M19**	**JHI-Hv50k-2016-140,869**	**STARP**	**GCAACAGGAACCAGCTATGACAGGTGAGCTTCCTCATGG**	**GACGCAAGTGAGCAGTATGACAGGTGAGCTTCCTTGTGA**	**ATTGCTTGCTCGACGCTTTG**
**M20**	**Sequence results**	**STARP**	**GCAACAGGAACCAGCTATGACCGCCGTTTTGCTCGTC**	**GACGCAAGTGAGCAGTATGACCGCCGTTTTGCCTGCA**	**AGAAAGCGCGAATACGTCCA**

### Physical localization of *Gpa1*

Fifteen putative protein-coding genes were identified in the fine-mapping interval according to the reference genome assembly [27] (Table 2). Of them, three putatively encode acyl-protein thioesterase 1-like proteins (*HORVU.MOREX.r2.2HG0177100/G1*, *HORVU.MOREX.r2.2HG0177110/G2*, and *HORVU.MOREX.r2.2HG0177120/G3*), and one each for EH domain-containing protein 1 (*HORVU.MOREX.r2.2HG0177170/G4*), ethylene-responsive transcription factor (*HORVU.MOREX.r2.2HG0177180/G6*), FAD-binding Berberine family protein (*HORVU.MOREX.r2.2HG0177170/G7*), and choline transporter-related family protein (*HORVU.MOREX.r2.2HG0177310/G15*). A gene cluster functionally related to photosynthesis or organelle biogenesis was also identified in the *Gpa1* region, including 4 blue copper genes with high similarity (*HORVU.MOREX.r2.2HG0177210/G7*, *HORVU.MOREX.r2.2HG0177220/G9*, *HORVU.MOREX.r2.2HG0177230/G10*, and *HORVU.MOREX.r2.2HG0177250/G12*), one DNA topoisomerase gene (*HORVU.MOREX.r2.2HG0177240/G11*), and 2 genes encoding putative PTOXs with homology to alternative oxidase (*HORVU.MOREX.r2.2HG0177260/G13* and *HORVU.MOREX.r2.2HG0177270/G14*).

**Table 2:** Predicted genes in the *Gpa1* region. G, gene

Gene number	Gene name	Homology
**G1**	**HORVU.MOREX.r2.2HG0177100**	**Acyl-protein thioesterase 1**
**G2**	**HORVU.MOREX.r2.2HG0177110**	**Acyl-protein thioesterase 1**
**G3**	**HORVU.MOREX.r2.2HG0177120**	**Acyl-protein thioesterase 1**
**G4**	**HORVU.MOREX.r2.2HG0177150**	**Uncharacterized protein**
**G5**	**HORVU.MOREX.r2.2HG0177170**	**EH domain-containing protein 1**
**G6**	**HORVU.MOREX.r2.2HG0177180**	**Ethylene-responsive transcription factor**
**G7**	**HORVU.MOREX.r2.2HG0177190**	**FAD-binding Berberine family protein**
**G8**	**HORVU.MOREX.r2.2HG0177210**	**Blue copper protein**
**G9**	**HORVU.MOREX.r2.2HG0177220**	**Blue copper protein**
**G10**	**HORVU.MOREX.r2.2HG0177230**	**Blue copper protein**
**G11**	**HORVU.MOREX.r2.2HG0177240**	**DNA gyrase/PPR protein**
**G12**	**HORVU.MOREX.r2.2HG0177250**	**Blue copper protein**
**G13**	**HORVU.MOREX.r2.2HG0177260**	**Alternative oxidase (Incomplete)**
**G14**	**HORVU.MOREX.r2.2HG0177270**	**Alternative oxidase**
**G15**	**HORVU.MOREX.r2.2HG0177310**	**Choline transporter-related family protein**

Blue copper protein function as an electron shuffler in electron transfer reactions, such as biological nitrogen fixation, respiration and photosynthesis. Structure analysis indicated the putative blue copper proteins, G7, G9, G10, and G12, in the *Gpa1* region contain a domain identified in plastocyanin, the long-range electron carrier between photosystems II and I [28]. Alternative oxidase is involved in the regulation of redox state of the electron transport chain in organelles [8, 9, 18]. Particularly, the putative coding product of *G14* shares high homology with IM (AT4G22260), the plastid terminal oxidase in *Arabidopsis* [8, 9]. Therefore, *G14* was named *HvPTOX* hereafter. For the *G11* gene, two different products, one PPR protein and one DNA gyrase, were predicted in the sense and antisense strand, respectively (Additional Fig. 2). The putative introns of the DNA gyrase gene contain coding exons in the reverse complementary strand for the predicted PRR gene, and vice versa (Additional Fig. 2). The EST match for the putative DNA gyrase was identified (FD525137), but we did not find ESTs for the predicted PPR from the available databases. DNA gyrase or topoisomerase has been linked to regulation of DNA replication and transcription during chloroplast biogenesis [29]. Although lacking EST matches, the predicted PPR protein is highly homologous to SVR7, one of the suppressors of the *Arabidopsis var2* mutation [19]. Therefore, members of this functionally related gene cluster were selected for further analysis.

### Selection of the *Gpa1* candidate

The four blue copper proteins within this region share at least 75% sequence identity, and the coding products of *G9* and *G12* vary by only one amino acid (aa) substitution. We speculated that these blue copper proteins may function redundantly, and mutation on one gene may not result in apparent phenotype change. Moreover, the AOX encoded by *G13* is incomplete, and was eliminated from further analysis. Using Bowman, BW397, Lyallpur and its isogenic mutant GSHO 519, we focused on identifying sequence polymorphism between the gene alleles in *G11* and *G14* (*HvPTOX*).

Although a few SNPs were detected between the Bowman and Morex alleles of *G11*, Bowman, BW397, GSHO 519 and Lyallpur share identical genomic sequences including the coding region. This suggested that *G11* might not be one of the candidates for *Gpa1*.

**Fig. 4:**
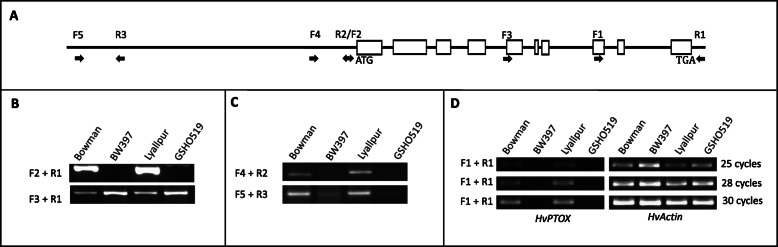
Gene structure and mutation analysis of the *HvPTOX* gene. The coding region of *HvPTOX* contains 10 exons (rectangles) and 9 introns (straight lines) (**a**). Various primers indicated with arrows (**a**) were used to analyze the mutated allele, and the primer sequences were included in Additional Table 2. Full-length of the coding region could not be obtained with the primer combination of F2 and R1 in mutants, but half of that at the 3′ was successfully amplified with primers F3 and R1 (**b**). The promoter (F4 and R2) and a region at the 2.5 kb upstream of the start codon (F5 and R3) were deleted altogether in mutants (**c**). RT-PCR analysis with 25, 28 and 30 PCR cycles failed to detect the expression of *Hvptox* in mutants. Actin was used as the internal control. The original gel images were included in Additional Figs. 3 and 4

Gene prediction and EST matches (DK626738, DK619131, BF626913, RUS39D06w and HB15J15r) showed that *HvPTOX* contains 10 coding exons and 9 introns (Fig. 4a). The full-length coding region was successfully amplified in Bowman and Lyallpur, but not in mutants of BW397 and GSHO519 (Fig. 4b, Additional Table 2). We only obtained the 3′ region of the coding sequence in the mutants (Fig. 4b, Additional Table 2). To capture the full length of *HvPTOX*, we conducted *FPNI-PCR* to acquire the coding sequence at the 5′ region. Sequencing of the product derived from *FPNI-PCR* indicated that the first three and almost half of the fourth exon were missing in the mutated allele. However, the sequence proximal to the fourth exon of *HvPTOX* in BW397 cannot be aligned to the assembled reference genome, and it is not homologous to any known protein coding sequences or transposable elements.

To determine if the gene mutation was caused by insertion or deletion, we designed more markers at the potential promoter and far-upstream regions (2.5 kb upstream of the start codon) of *HvPTOX*. Our result indicated that those markers were all missing in mutants (Fig. 4c). In line with the deletion of the promoter, RT-PCR confirmed that *HvPTOX* was not expressed in mutants (Fig. 4d). Its expression in mutants could not be detected even with 30 PCR cycles, in contrast to the expression abundance indicated by the 25 cycles in wild types (Fig. 4d). Quantitative real-time PCR (qRT-PCR) also revealed that the expression of *gpa1* is significantly suppressed in mutants (Additional Fig. 5). Although the size of the deletion was unknown, *HvPTOX* structure, expression and thus function was totally disrupted in mutants. Therefore, the *HvPTOX* gene (*G4*/*HORVU.MOREX.r2.2HG0177270*) was selected as a strong candidate. The identity of *Gpa1* still needs to be further verified in homologous or heterologous systems.

## Discussion

Plant chloroplast biogenesis is important for biomass and economic yield. Variegation mutants provide an ideal model to understand the development of functional chloroplasts. The allelic mutants of *gpa* has long been identified, but their genetic basis underlying variegation remains unknown. In the present study, we characterized the chloroplast ultrastructure of the *gpa1.a* mutant and localized the corresponding locus. The lack of chlorophyll in the white stripes of the mutant is caused by the interruption of chloroplast biogenesis. The *Gpa1* gene was delimited within a 410-kb region containing a cluster of functionally related genes. A genomic deletion in the PTOX-encoding gene in mutants abolished its gene function, suggesting it is a promising candidate for *Gpa1*.

Many functionally related genes are distributed nonrandomly throughout the genome as functional clusters in eukaryotes [30]. In plants, many biosynthetic gene clusters for secondary metabolism have been identified [31]. Those clusters may have arose from recruitment of genes from elsewhere in the genome through duplication and neofunctionalization, but not by horizontal gene transfer from prokaryotes [32]. From an evolutionary perspective, the physical clustering of functionally related genes may facilitate coordinated gene expression and prevent the breakup of co-adapted alleles during recombination [32, 33]. The cluster members in the *Gpa1* region may target chloroplast for photosynthesis or chloroplast development. However, their actual functional roles require further investigation. Using CRISPR technology to knock out the clustered genes individually will possibly solve this puzzling question.

Although *G11* may not be *Gpa1*, it is interesting to discover if two different genes are derived from the same genomic fragment. Eukaryotic operon-like transcription has been observed, and one mRNA can be translated to several separate products in the cytoplasm [34, 35]. However, there have been no reports for two independent genes harbored on reverse complementary strands. The lack of EST matches for the PRR-coding gene suggests that it may not be a true gene, but this conclusion needs to be confirmed with rapid amplification of cDNA ends (RACE).

The PTOX protein encoded by *G14* shares high identity with IM in *Arabidopsis*. The variegation mutant *im* in Arabidopsis and the orthologous *ghost* mutant in tomato both display the loss of PTOX caused defect in chloroplast biosynthesis [9, 36]. It was discovered that PTOX functions as a terminal oxidase in controlling the redox state of the plastoquinone (PQ) pool in developing and mature thylakoids [37, 38]. PTOX is involved in transferring electrons from plastoquinol (PQH2) to molecular oxygen, forming water and PQ [38]. The role in regulation of the redox state of the photosynthetic apparatus makes PTOX crucial for a growing number of biochemical pathways, such as the desaturation reactions in carotenoid biosynthesis, chlororespiration, PSI cyclic electron flow, and photoprotection [37, 39, 40].

Biosynthesis of carotenoid is impaired in the *im* mutant due to the lack of phytoene desaturation (PDS), and the intermediate phytoene is accumulated in the white sections of leaves [8, 9]. The white areas of *im* might accumulate white photodamaged plasmids due to the lack of photoprotective carotenoid [10]. It was also demonstrated that the redox state of the PQ pool may control chloroplast biogenesis as a potent initiator of retrograde signaling [37, 41]. Under these scenarios, the predicted *HvPTOX* (*HORVU.MOREX.r2.2HG0177270*) in the *Gpa1* region was designated a strong candidate. This series of *gpa* mutants were identified almost 80 years ago, and fine localization of *Gpa1* in this study has provided candidates for functional validation, facilitating cloning of this long-elusive gene.

## Conclusions

In summary, we have characterized and genetically mapped the *gpa1.a* mutation causing a variegation phenotype in barley. Our results indicate that chloroplast biogenesis is defective in white sections of the mutant, and the *gpa1* mutation imposes a systemic effect on barley growth and development. The *Gpa1* gene was mapped to chromosome 2H within a 410 kb region. In addition, we have shown that *Gpa1* is harbored in a gene cluster functionally related to photosynthesis or the chloroplast. Further studies have indicated that a genomic deletion disrupts the expression and function of the PTOX-encoding gene, *G14*/*HORVU.MOREX.r2.2HG0177270*. Therefore, the present study paves the way for the cloning of *Gpa1*, which will improve our understanding of the molecular mechanisms underlying chloroplast biogenesis.

## Methods

### Plant materials

An F_2_ segregating population derived from the cross between Bowman (wild type) and BW397 (the *gpa1.a* mutant) was used for genetic mapping of the *Gpa1* gene. The *gpa1.a* mutation was donated by GSHO519 carrying the genetic background of Lyallpur. Seeds of Bowman, BW397, GSHO519 and Lyallpur were obtained from the USDA-ARS National Plant Germplasm System (NPGS). A total of 510 F_2_ plants were used to generate a genetic map. All plants together with parents were grown in a greenhouse under a 16 h light/8 h dark photoperiod at 25 °C. Phenotyping was conducted at the 1st leaf stage and repeated at the boot stage. Phenotype of F_2_ recombinants delimiting the *Gpa1* region was confirmed with 30 F_3_ individuals.

### DNA extraction

DNA was extracted according to the CTAB protocol [42]. Around 100 mg leaf samples were collected from plants at the three-leaf stage and quantified using a NanoDrop spectrophotometer (NanoDrop 8000, Thermo Fisher Scientific) according to the manufacturer’s instructions. The final concentration was adjusted to 100 ng/uL for PCR application.

### SNP genotyping and marker development

Forty-eight F_2_s (24 each for wild type and stripped) and parental lines were first genotyped with a barley 50 k iSelect SNP Array [43]. Marker positions are based on the barley pseudo-molecule assembly of Morex V1 [44]. Genotype calling was performed with the de novo calling algorithm in GenomeStudio (Illumina). Clusters of polymorphic SNPs were inspected and manually adjusted if necessary. The linked SNPs were used to develop semi-thermal asymmetric reverse PCR (STARP) markers to genotype the F_2_ population [45]. PCR was conducted in a 10 μl reaction volume with Taq DNA polymerase (New England Biolab) according to the manufacturer’s instructions. Sequences of priming element-adjustable primer (PEA-primer) 1 and 2 are 5′-AGCTGGTT-SP9-GCAACAGGAACCAGCTATGAC-3′ and 5′-ACTGCTCAAGAG-SP9-GACGCAAGTGAGCAGTATGAC-3′, respectively [45]. The thermal amplification program followed a touchdown protocol as described previously [45, 46]. Stained with GelRed™ nucleic acid stain (MilliporeSigma), amplicons were analyzed with 6% polyacrylamide gel which was imaged using a Typhoon™ FLA 9500 variable mode laser scanner (GE Healthcare Life Sciences, Marlborough, MA). The markers used in the present study are listed in Table 1.

### Physical mapping and gene prediction

The genome sequences of barley cv Morex v2 and Golden Promise v1 were used for marker localization and physical mapping [27, 47, 48]. Gene predication and annotation was conducted with the programs of FGENESH and Pfam 32.0, respectively [49, 50]. Gene annotation was also confirmed with the BLASTP program.

### Gene expression analysis

Gene expression analysis was conducted with semi-quantitative reverse transcription (RT)-PCR and quantitative real-time PCR (qRT-PCR). RNA isolated from leaves of booting plants was used for gene analysis and cDNA sequencing. RNA was extracted using the NucleoSpin RNA Plant kit (Macherey-Nagel, Düren, Germany). First-strand cDNA was synthesized using M-MLV Reverse Transcriptase (Invitrogen). The PCR amplification was conducted in a 20 μl reactions containing 2 μl cDNA (equivalent to 50 ng of total RNA), 2 μl of each gene-specific primer (2.5 μM), 10 μl of water, 0.5 unit Taq DNA Polymerase (New England Biolabs), 2 μl dNTPs (2.5 μM) and 2 μl of 10 x Standard Taq reaction buffer (New England Biolabs). The actin gene was used as a control. PCR primers were as follows: *HvActin*, 5′-GAGCACGGTATCGTAAGCAACTG-3′ and 5′-CCTGTTCATAATCAAGGGCAACG-3′; *HORVU.MOREX.r2.2HG0177270*, F1: 5′-CCAGCTCCAGAGGTGGCTGT-3′, R1: 5′- CAGCGCTCTAGCACGGAGGT-3′.

Fluorescent qRT-PCR was performed with three replications using the 7500 Fast Real-Time PCR system (Applied Biosystems, Grand Island, NY, USA). Amplification was conducted in a 10 μl reaction volume containing 2 μL cDNA (equivalent to 50 ng of total RNA), 1.5 μL of each gene-specific primer (2.5 μM), and 5 μL of SYBR Select Master Mix (Applied Biosystems). The actin gene was used as the internal control for real-time analysis and was amplified with primers 5′-AACTATGTTCCCAGGTATCGC-3′ and 5′-GACTCGTCGTACTCATCCTTG-3′. Primers 5′-GAAACTACCAGCTCCAGAGG-3′ and 5′-GCGTTTGACATGCCTTCATC-3′ were used to target *Gpa1* gene expression. Amplification conditions used were the same as described previously [51].

### Chlorophyll measurement

Chlorophyll contents were measured by spectrophotometric determination with 4 replications [52]. Briefly, 0.2 g fresh leaf tissue of wild type and mutant plants at booting stage was collected and soaked in 5 ml of 95% ethanol in the dark for 48 h. After 5 min of centrifugation at 3000 rpm, the residual plant debris was removed. Supernatant was measured with a spectrophotometer (Beckman DU 7400) at 663 nm for chlorophyll a and 645 nm for chlorophyll b.

### Fusion primer and nested integrated PCR (*FPNI-PCR*)

*FPNI-PCR* was performed for chromosome walking to identify the unknown genomic region [53]. Gene-specific primers used for primary, secondary and tertiary PCR respectively are, GSP1, 5′-CTGCACTCAATAGGCAGGGTGT-3′; GSP2, 5′-ACCGAGTCGCAACCAGCCTT-3′; and GSP3, 5′-TTGCCACCCAACGCCTGACA-3′. Nine fusion arbitrary degenerate primers (FP1–9) and FP-specific primers (FSP1 and FSP2) were designed according to Wang et al. (2011). LA Taq (Takara Bio USA Inc., Mountain View, CA) was used in the first round of PCR. The 20 μl reactions consisted of 10 μM FP primer and 2 μM GSP1 with all other reagent concentrations following the recommended LA Taq protocol. The primary round of PCR in the *FPNI-PCR* procedure includes high stringency PCR cycles (94 °C for 10 s, 62 °C for 30 s, 72 °C for 2 m, repeated two times), followed by one low stringency PCR cycle (94 °C for 15 s, 25 °C for 1 min, 28 °C ramping up 0.2C/sec for 3 min, and 72 °C 2:30 min). This high and low stringency cycle pattern was repeated six times. The primary round of PCR was finished with another two high stringency cycles followed by one cycle of 94 °C for 10 s, 44 °C for 30 s, 72 °C for 2 min, and a final extension with 72 °C for 5 min. The PCR product was diluted to one half and used as template for the next round of PCR. Phusion Hot Start Flex Polymerase (NEB, Ipswich, MA) was used for both secondary and tertiary rounds of PCR in 20 μl reactions containing 10 μM of FSP primer, 2 μM of GSP primer and all other reagents with the recommended concentrations by the manufacturer’s protocol. A 1/40 dilution of the secondary round PCR product was used as template for the tertiary round of PCR. Samples were visualized on 1% agarose gel stained with ethidium bromide under UV illumination, and amplified bands were extracted and purified using a QIAquick Gel Extraction Kit (Qiagen, Hilden, Germany) for sequencing.

### Transmission electron microscopy analysis

Leaf samples collected from Bowman and BW397 plants at the booting stage were fixed with 2.5% glutaraldehyde in 0.1 M sodium phosphate buffer, pH 7.35 (Tousimis Research Corporation, Rockville, MD) at 4 °C. Sample pretreatment and section preparation followed the protocol described in [54]. Stained with lead citrate for two minutes, dried sections on copper grids were visualized on a JEOL JEM-100CX II electron microscope (JEOL Inc., Peabody, MA).

## Supplementary Information

**Additional file 1: Figure 1** Phenotypic comparison between Bowman and BW397. The BW397 mutant produces white anthers (A), spike, awns and flag leaf (B). **Figure 2** Gene prediction with the genomic sequence of G11.One PPR protein and DNA gyrase were predicated in sense and antisense strand, respectively. Exons were indicated with rectangles, and straight lines for introns. E, exon; I, intron. The gene structures are drawn to scale. **Figure 3** The original gel image cropped for Fig. 4band c. Samples were arranged in the following order (from left to right): Bowman, BW397, Lyallpur and GSHO519. The full-length coding region (F2 + R1, shown in Fig. 4b) and the putative promoter (F4 + R2, shown in Fig. 4c) could not be amplified in mutants, but half of the coding sequence at the 3′ was successfully obtained with primers F3 and R1 (Fig. 4b) in all genotypes. A length polymorphism of 35 bp was detected between promoters (F4 + R2) of Bowman and Lyallpur, which was confirmed by sequencing. A genomic region at 2.5 kb upstream of the start codon (F5 and R3, shown in Fig. 4c) were not amplified in mutants, either. The cropped areas were indicated with white dashed rectangles. **Figure 4** The original gel image of RT-PCR cropped for Fig. 4d. RT-PCR analysis with 25, 28 and 30 PCR cycles failed to detect the expression of *Hvptox* in mutants. Actin was used as the internal control. Samples were arranged in the following order (from left to right): Bowman, BW397, Lyallpur and GSHO519. The cropped areas were indicated with white dashed rectangles. **Figure 5** Quantitative real-time PCR (qRT-PCR) analysis of *Gpa1* alleles.

**Additional file 2: Table 1** SNP genotyping of 31 F2s and their parental lines with the barley 50 k iSelect SNP Array. G-1 to G-16 are F2s with normal phenotype, and g-1 to g-15 are variagated F2s. Genotypes for called SNPs were converted to the codes of pink “A”, green “B”, yellow “H” and blank “-”. The WT paent Bowman confers the pink ‘A’ genotype, “B” for the mutant BW397, “H” for heterozygous, and missing data as “-”. The flankeding SNPs, JHI-Hv50k-2016-139,629 and JHI-Hv50k-2016-142,540, were highlighted in yellow. **Table 2.** Primers used for the analysis of HvPTOX alleles.

## Data Availability

All data generated or analyzed during this study are included in this published article and its supplementary information files. Sequence data from this article can be found at figshare with the digital object identifier (DOI) 10.6084/m9.figshare.14153465.v1.

## Abbreviations

*gpa1*: *grandpa 1*
*im*: *immutans*
*var2*: *variegated 2*
PTOX: Plastid terminal oxidase
AOX: Alternative oxidase
*FtsH2*: *Filamentous temperature-sensitive H2*
Crr2–2: Chlororespiratory Reduction 2–2
PGR5: Proton Gradient Regulation 5
PPR: Pentatricopeptide repeat
SVR: Suppressor of Variegation
FUG1: Fu-gaeri1
SCO1: Snow Cotyledon 1
TEM: Transmission electron microscopy
STARP: Semi-thermal asymmetric reverse PCR
*FPNI-PCR*: Fusion primer and nested integrated PCR
FP: Fusion arbitrary degenerate primer
FSP: FP-specific primers
NPGS: National Plant Germplasm System

## References

[1] Kretschmer M, Damoo D, Djamei A, Kronstad J. Chloroplasts and plant immunity: where are the fungal effectors? Pathogens. 2019;9(1):19. 10.3390/pathogens9010019 PMC7168614 31878153

[2] Serrano I, Audran C, Rivas S (2016). Chloroplasts at work during plant innate immunity. J Exp Bot.

[3] Toufexi A, Duggan C, Pandey P, Savage Z, Segretin ME, Yuen LH, Gaboriau DCA, Leary AY, Khandare V, Ward AD, et al. Chloroplasts navigate towards the pathogen interface to counteract infection by the Irish potato famine pathogen. bioRxiv. 2019;10:1101.

[4] Nott A, Jung HS, Koussevitzky S, Chory J (2006). Plastid-to-nucleus retrograde signaling. Annu Rev Plant Biol.

[5] Jung HS, Chory J (2010). Signaling between chloroplasts and the nucleus: can a systems biology approach bring clarity to a complex and highly regulated pathway?. Plant Physiol.

[6] Li HM, Chiu CC (2010). Protein transport into chloroplasts. Annu Rev Plant Biol.

[7] Yu F, Fu A, Aluru M, Park S, Xu Y, Liu H, Liu X, Foudree A, Nambogga M, Rodermel S (2007). Variegation mutants and mechanisms of chloroplast biogenesis. Plant Cell Environ.

[8] Wu D, Wright DA, Wetzel C, Voytas DF, Rodermel S (1999). The *IMMUTANS* variegation locus of *Arabidopsis* defines a mitochondrial alternative oxidase homolog that functions during early chloroplast biogenesis. Plant Cell.

[9] Carol P, Stevenson D, Bisanz C, Breitenbach J, Sandmann G, Mache R, Coupland G, Kuntz M (1999). Mutations in the *Arabidopsis* gene *IMMUTANS* cause a variegated phenotype by inactivating a chloroplast terminal oxidase associated with phytoene desaturation. Plant Cell.

[10] Foudree A, Putarjunan A, Kambakam S, Nolan T, Fussell J, Pogorelko G, Rodermel S (2012). The mechanism of variegation in immutans provides insight into chloroplast biogenesis. Front Plant Sci.

[11] Suzuki CK, Rep M, van Dijl JM, Suda K, Grivell LA, Schatz G (1997). ATP-dependent proteases that also chaperone protein biogenesis. Trends Biochem Sci.

[12] Takechi K, Sodmergen MM, Motoyoshi F, Sakamoto W (2000). The *YELLOW VARIEGATED* (*VAR2*) locus encodes a homologue of FtsH, an ATP-dependent protease in *Arabidopsis*. Plant Cell Physiol.

[13] Chen M, Choi Y, Voytas DF, Rodermel S (2000). Mutations in the *Arabidopsis VAR2* locus cause leaf variegation due to the loss of a chloroplast FtsH protease. Plant J.

[14] Zhang D, Kato Y, Zhang L, Fujimoto M, Tsutsumi N, Sodmergen SW (2010). The FtsH protease heterocomplex in *Arabidopsis*: dispensability of type-B protease activity for proper chloroplast development. Plant Cell.

[15] Kato Y, Miura E, Ido K, Ifuku K, Sakamoto W (2009). The variegated mutants lacking chloroplastic FtsHs are defective in D1 degradation and accumulate reactive oxygen species. Plant Physiol.

[16] Wang L, Kim C, Xu X, Piskurewicz U, Dogra V, Singh S, Mahler H, Apel K (2016). Singlet oxygen- and EXECUTER1-mediated signaling is initiated in grana margins and depends on the protease FtsH2. Proc Natl Acad Sci U S A.

[17] Sakamoto W, Zaltsman A, Adam Z, Takahashi Y (2003). Coordinated regulation and complex formation of *yellow variegated1* and *yellow variegated2*, chloroplastic FtsH metalloproteases involved in the repair cycle of photosystem II in *Arabidopsis* thylakoid membranes. Plant Cell.

[18] Okegawa Y, Kobayashi Y, Shikanai T (2010). Physiological links among alternative electron transport pathways that reduce and oxidize plastoquinone in *Arabidopsis*. Plant J.

[19] Liu X, Yu F, Rodermel S (2010). An *Arabidopsis* pentatricopeptide repeat protein, *SUPPRESSOR OF VARIEGATION7*, is required for FtsH-mediated chloroplast biogenesis. Plant Physiol.

[20] Druka A, Franckowiak J, Lundqvist U, Bonar N, Alexander J, Houston K, Radovic S, Shahinnia F, Vendramin V, Morgante M (2011). Genetic dissection of barley morphology and development. Plant Physiol.

[21] Li M, Hensel G, Mascher M, Melzer M, Budhagatapalli N, Rutten T, Himmelbach A, Beier S, Korzun V, Kumlehn J (2019). Leaf variegation and impaired chloroplast development caused by a truncated CCT domain gene in albostrians barley. Plant Cell.

[22] Ehrenberg L, Gustafsson A, Lundquist U (1961). Viable mutants induced in barley by ionizing radiations and chemical mutagens. Hereditas.

[23] Matchett RW, Pollock BM, Robertson DW (1968). The "*grandpa*" gene: a chlorophyll mutation in *Hordeum* species. J Hered.

[24] Immer FR, Henderson MT (1943). Linkage studies in barley. Genetics.

[25] Matchett RW, Nass HG, Robertson DW (1971). Inheritance and linkage studies with the grandpa gene in barley, *Hordeum vulgare* L. Can J Genet Cytol.

[26] Muñoz-Amatriaín M, Moscou MJ, Bhat PR, Svensson JT, Bartoš J, Suchánková P, Šimková H, Endo TR, Fenton RD, Lonardi S (2011). An improved consensus linkage map of barley based on flow-sorted chromosomes and single nucleotide polymorphism markers. The Plant Genome.

[27] Beier S, Himmelbach A, Colmsee C, Zhang XQ, Barrero RA, Zhang Q, Li L, Bayer M, Bolser D, Taudien S (2017). Construction of a map-based reference genome sequence for barley, ***Hordeum vulgare*** L. Sci Data.

[28] Hohner R, Pribil M, Herbstova M, Lopez LS, Kunz HH, Li M, Wood M, Svoboda V, Puthiyaveetil S, Leister D (2020). Plastocyanin is the long-range electron carrier between photosystem II and photosystem I in plants. Proc Natl Acad Sci U S A.

[29] Pfalz J, Pfannschmidt T (2013). Essential nucleoid proteins in early chloroplast development. Trends Plant Sci.

[30] Cera A, Holganza MK, Hardan AA, Gamarra I, Eldabagh RS, Deschaine M, Elkamhawy S, Sisso EM, Foley JJt, Arnone JT. Functionally related genes cluster into genomic regions that coordinate transcription at a distance in *Saccharomyces cerevisiae*. mSphere. 2019;4(2):e00063–19. 10.1128/mSphere.00063-19 PMC6416364 30867326

[31] Hen-Avivi S, Savin O, Racovita RC, Lee WS, Adamski NM, Malitsky S, Almekias-Siegl E, Levy M, Vautrin S, Berges H (2016). A metabolic gene cluster in the wheat *W1* and the barley *Cer-cqu* loci determines beta-diketone biosynthesis and glaucousness. Plant Cell.

[32] Nutzmann HW, Scazzocchio C, Osbourn A (2018). Metabolic gene clusters in eukaryotes. Annu Rev Genet.

[33] Ben-Shahar Y, Nannapaneni K, Casavant TL, Scheetz TE, Welsh MJ (2007). Eukaryotic operon-like transcription of functionally related genes in *Drosophila*. Proc Natl Acad Sci U S A.

[34] Blumenthal T (2004). Operons in eukaryotes. Brief Funct Genomic Proteomic.

[35] Kominek J, Doering DT, Opulente DA, Shen XX, Zhou X, DeVirgilio J, Hulfachor AB, Groenewald M, McGee MA, Karlen SD (2019). Eukaryotic acquisition of a bacterial operon. Cell.

[36] Josse EM, Simkin AJ, Gaffe J, Laboure AM, Kuntz M, Carol P (2000). A plastid terminal oxidase associated with carotenoid desaturation during chromoplast differentiation. Plant Physiol.

[37] Kambakam S, Bhattacharjee U, Petrich J, Rodermel S (2016). PTOX mediates novel pathways of electron transport in etioplasts of *Arabidopsis*. Mol Plant.

[38] Peng L, Shimizu H, Shikanai T (2008). The chloroplast NAD(P) H dehydrogenase complex interacts with photosystem I in *Arabidopsis*. J Biol Chem.

[39] Peltier G, Cournac L (2002). Chlororespiration. Annu Rev Plant Biol.

[40] Rumeau D, Peltier G, Cournac L (2007). Chlororespiration and cyclic electron flow around PSI during photosynthesis and plant stress response. Plant Cell Environ.

[41] Pfannschmidt T, Brautigam K, Wagner R, Dietzel L, Schroter Y, Steiner S, Nykytenko A (2009). Potential regulation of gene expression in photosynthetic cells by redox and energy state: approaches towards better understanding. Ann Bot.

[42] Murray MG, Thompson WF (1980). Rapid isolation of high molecular weight plant DNA. Nucleic Acids Res.

[43] Bayer MM, Rapazote-Flores P, Ganal M, Hedley PE, Macaulay M, Plieske J, Ramsay L, Russell J, Shaw PD, Thomas W (2017). Development and evaluation of a barley 50k iSelect SNP array. Front Plant Sci.

[44] Mascher M, Gundlach H, Himmelbach A, Beier S, Twardziok SO, Wicker T, Radchuk V, Dockter C, Hedley PE, Russell J (2017). A chromosome conformation capture ordered sequence of the barley genome. Nature.

[45] Long YM, Chao WS, Ma GJ, Xu SS, Qi LL (2017). An innovative SNP genotyping method adapting to multiple platforms and throughputs. Theor Appl Genet.

[46] Faris JD, Overlander ME, Kariyawasam GK, Carter A, Xu SS, Liu Z (2020). Identification of a major dominant gene for race-nonspecific tan spot resistance in wild emmer wheat. Theor Appl Genet.

[47] Mascher M (2019). Pseudomolecules and annotation of the second version of the reference genome sequence assembly of barley cv. Morex [Morex V2].

[48] Schreiber M, Mascher M, Wright J, Padmarasu S, Himmelbach A, Heavens D, Milne L, Clavijo BJ, Stein N, Waugh R (2020). A genome assembly of the barley 'transformation reference' cultivar golden promise. G3 (Bethesda).

[49] Bateman A, Coin L, Durbin R, Finn RD, Hollich V, Griffiths-Jones S, Khanna A, Marshall M, Moxon S, Sonnhammer EL (2004). The Pfam protein families database. Nucleic Acids Res.

[50] Solovyev V, Salamov A (1997). The gene-finder computer tools for analysis of human and model organisms genome sequences. Proc Int Conf Intell Syst Mol Biol.

[51] Yu X, Qin Q, Wu X, Li D, Yang S (2020). Genetic and physical localization of the gene controlling leaf pigmentation pattern in *Medicago truncatula*. G3 (Bethesda).

[52] Arnon DI (1949). Copper enzymes in isolated chloroplasts. Polyphenoloxidase in *Beta Vulgaris*. Plant Physiol.

[53] Wang Z, Ye S, Li J, Zheng B, Bao M, Ning G (2011). Fusion primer and nested integrated PCR (FPNI-PCR): a new high-efficiency strategy for rapid chromosome walking or flanking sequence cloning. BMC Biotechnol.

[54] Rajamohan A, Rinehart JP, Foster SP, Leopold RA (2013). Permeability barriers to embryo cryopreservation of *Pectinophora gossypiella* (Lepidoptera: Gelechiidae). J Econ Entomol.

