# Supplementary material for: Genetic analysis of the barley variegation mutant, grandpa1.a
Source: BMC Plant Biol. 2021 Mar 13;21:134. doi: 10.1186/s12870-021-02915-9 (PMC7955646; doi:10.1186/s12870-021-02915-9)
Supplement: Supplementary file 1 — Additional file 1: Figure 1 Phenotypic comparison between Bowman and BW397. The BW397 mutant produces white anthers (A), spike, awns and flag leaf (B). Figure 2 Gene prediction with the genomic sequence of G11.One PPR protein and DNA gyrase were predicated in sense and antisense strand, respectively. Exons were indicated with rectangles, and straight lines for introns. E, exon; I, intron. The gene structures are drawn to scale. Figure 3 The original gel image cropped for Fig. 4band c. Samples were arranged in the following order (from left to right): Bowman, BW397, Lyallpur and GSHO519. The full-length coding region (F2 + R1, shown in Fig. 4b) and the putative promoter (F4 + R2, shown in Fig. 4c) could not be amplified in mutants, but half of the coding sequence at the 3′ was successfully obtained with primers F3 and R1 (Fig. 4b) in all genotypes. A length polymorphism of 35 bp was detected between promoters (F4 + R2) of Bowman and Lyallpur, which was confirmed by sequencing. A genomic region at 2.5 kb upstream of the start codon (F5 and R3, shown in Fig. 4c) were not amplified in mutants, either. The cropped areas were indicated with white dashed rectangles. Figure 4 The original gel image of RT-PCR cropped for Fig. 4d. RT-PCR analysis with 25, 28 and 30 PCR cycles failed to detect the expression of Hvptox in mutants. Actin was used as the internal control. Samples were arranged in the following order (from left to right): Bowman, BW397, Lyallpur and GSHO519. The cropped areas were indicated with white dashed rectangles. Figure 5 Quantitative real-time PCR (qRT-PCR) analysis of Gpa1 alleles. [file 12870_2021_2915_MOESM1_ESM.pptx]

## Slide 1
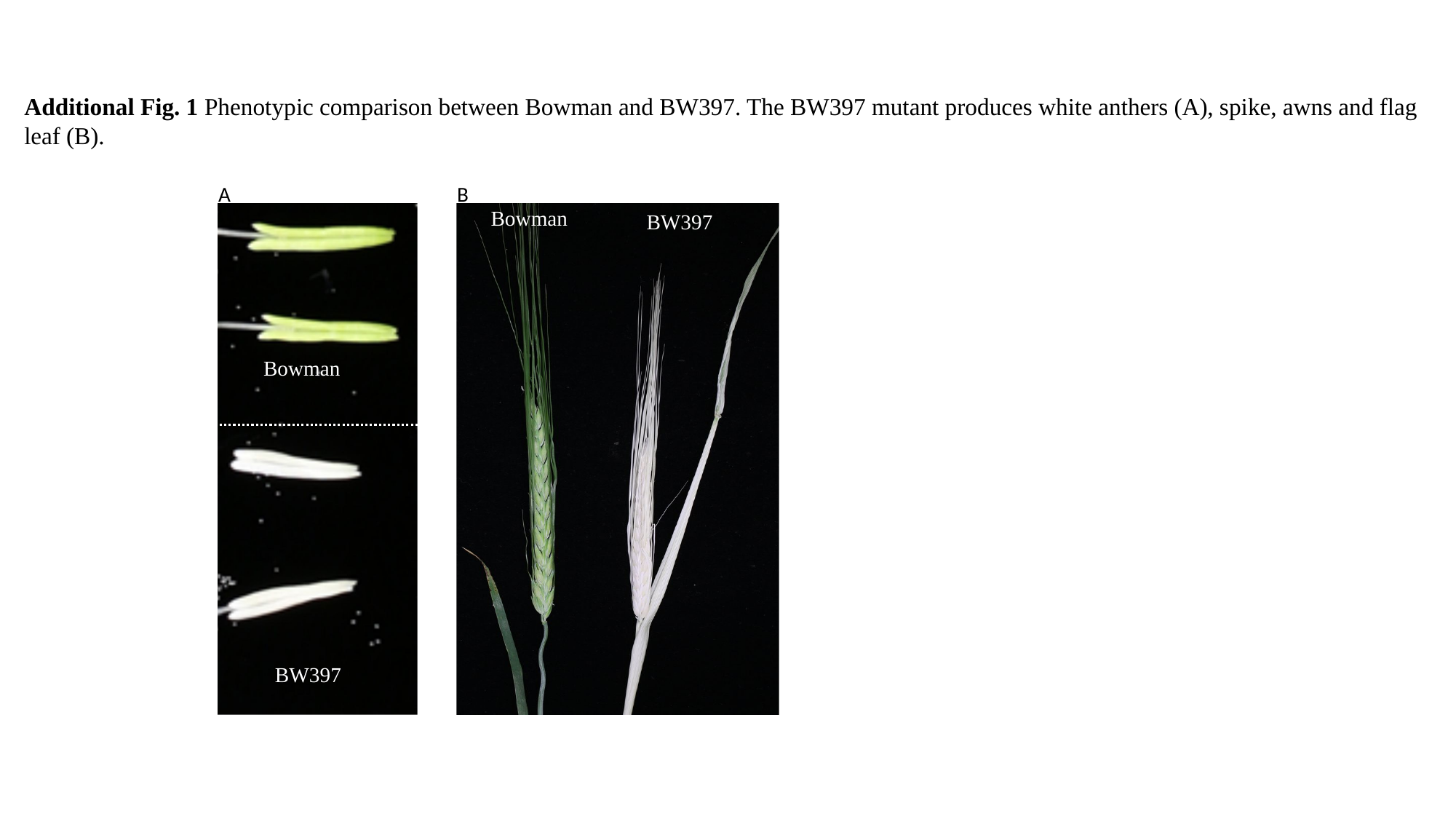

Additional Fig. 1 Phenotypic comparison between Bowman and BW397. The BW397 mutant produces white anthers (A), spike, awns and flag leaf (B).
A
B
Bowman
BW397
Bowman
BW397

## Slide 2
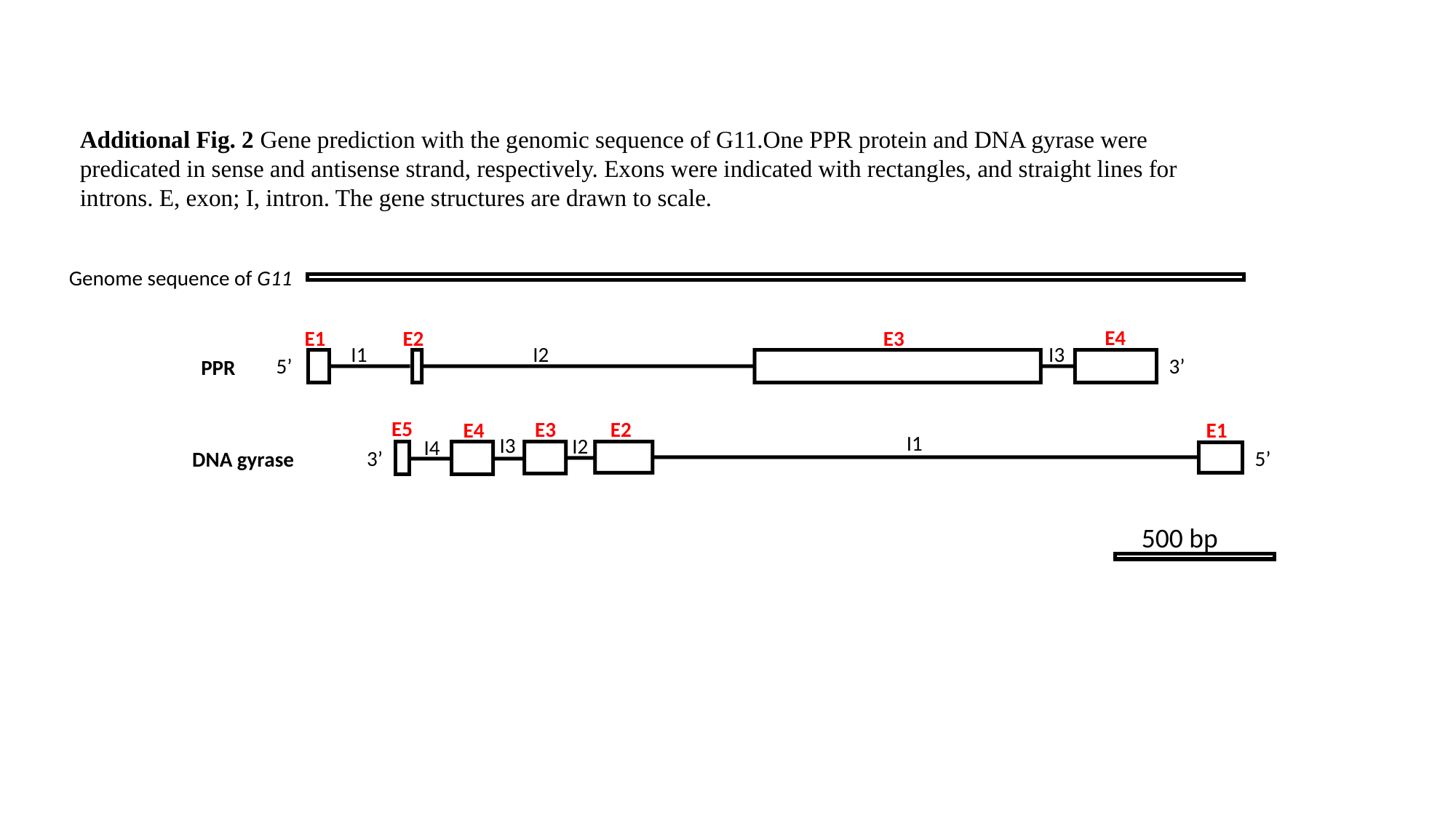

Additional Fig. 2 Gene prediction with the genomic sequence of G11.One PPR protein and DNA gyrase were predicated in sense and antisense strand, respectively. Exons were indicated with rectangles, and straight lines for introns. E, exon; I, intron. The gene structures are drawn to scale.
Genome sequence of G11
E4
E2
E1
E3
I3
I1
I2
5’
3’
PPR
E5
E3
E2
E4
E1
I1
I3
I2
I4
3’
5’
DNA gyrase
500 bp

## Slide 3
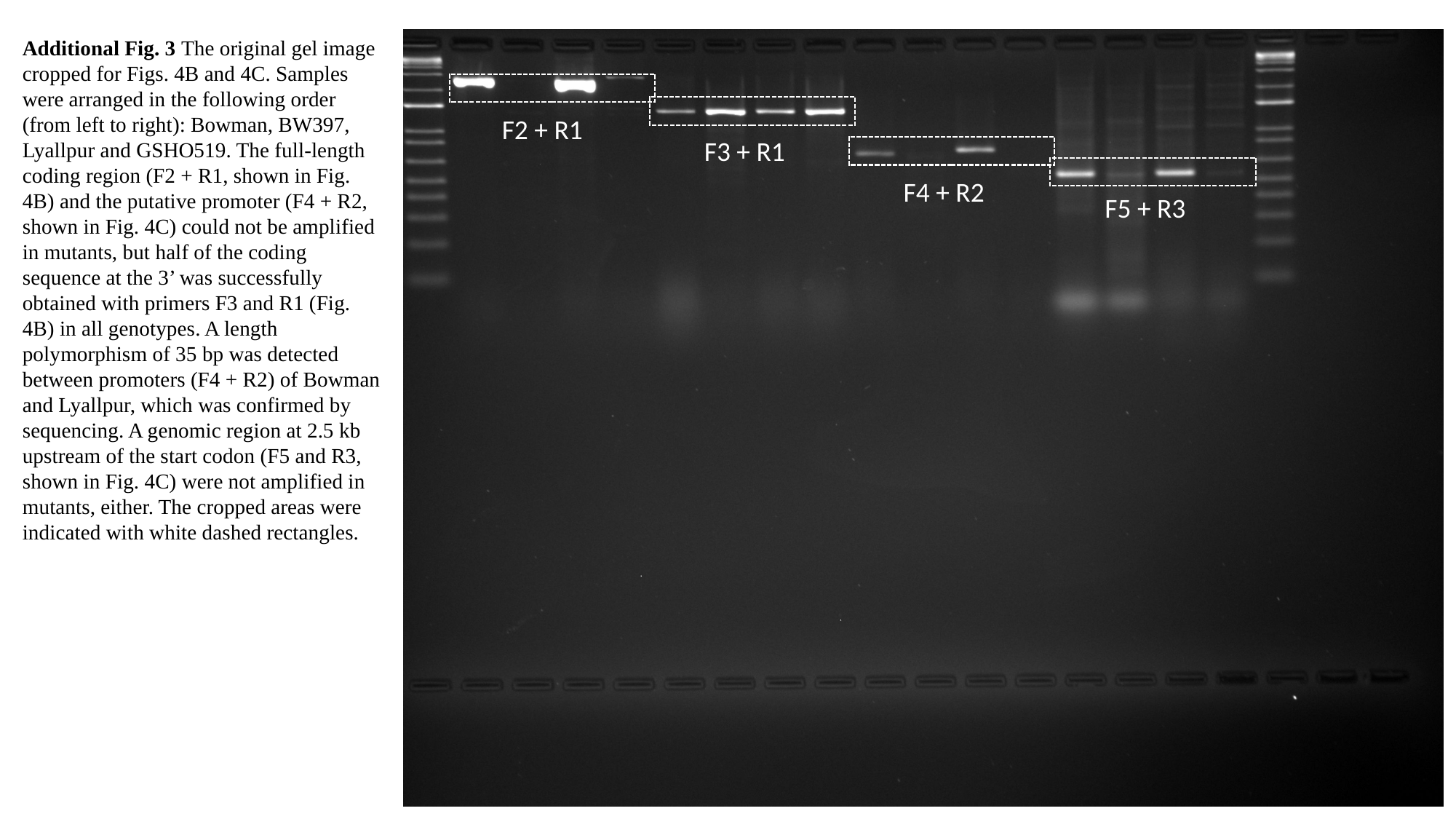

Additional Fig. 3 The original gel image cropped for Figs. 4B and 4C. Samples were arranged in the following order (from left to right): Bowman, BW397, Lyallpur and GSHO519. The full-length coding region (F2 + R1, shown in Fig. 4B) and the putative promoter (F4 + R2, shown in Fig. 4C) could not be amplified in mutants, but half of the coding sequence at the 3’ was successfully obtained with primers F3 and R1 (Fig. 4B) in all genotypes. A length polymorphism of 35 bp was detected between promoters (F4 + R2) of Bowman and Lyallpur, which was confirmed by sequencing. A genomic region at 2.5 kb upstream of the start codon (F5 and R3, shown in Fig. 4C) were not amplified in mutants, either. The cropped areas were indicated with white dashed rectangles.
F2 + R1
F3 + R1
F4 + R2
F5 + R3

## Slide 4
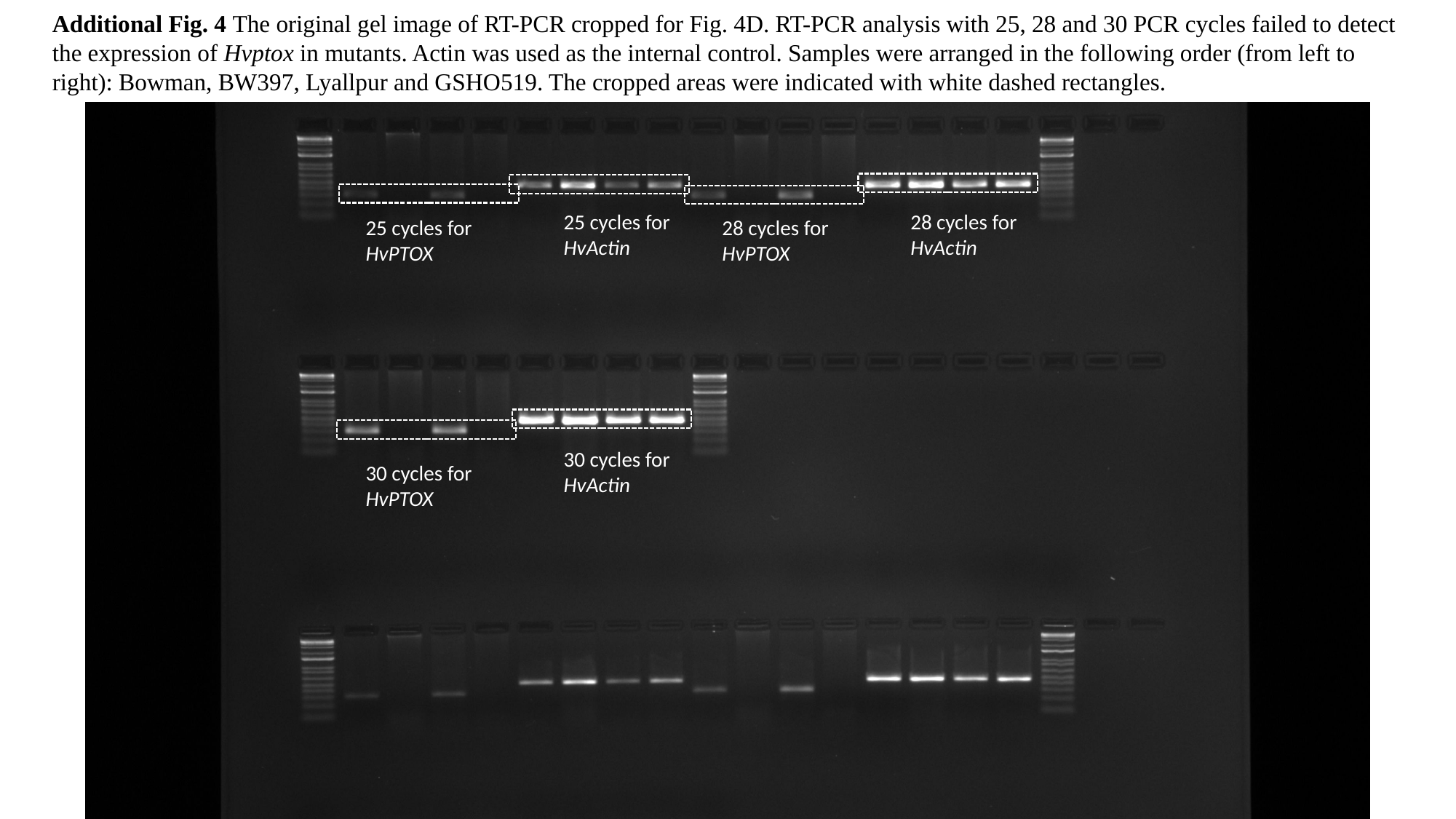

Additional Fig. 4 The original gel image of RT-PCR cropped for Fig. 4D. RT-PCR analysis with 25, 28 and 30 PCR cycles failed to detect the expression of Hvptox in mutants. Actin was used as the internal control. Samples were arranged in the following order (from left to right): Bowman, BW397, Lyallpur and GSHO519. The cropped areas were indicated with white dashed rectangles.
25 cycles for HvActin
28 cycles for HvActin
25 cycles for HvPTOX
28 cycles for HvPTOX
30 cycles for HvActin
30 cycles for HvPTOX

## Slide 5
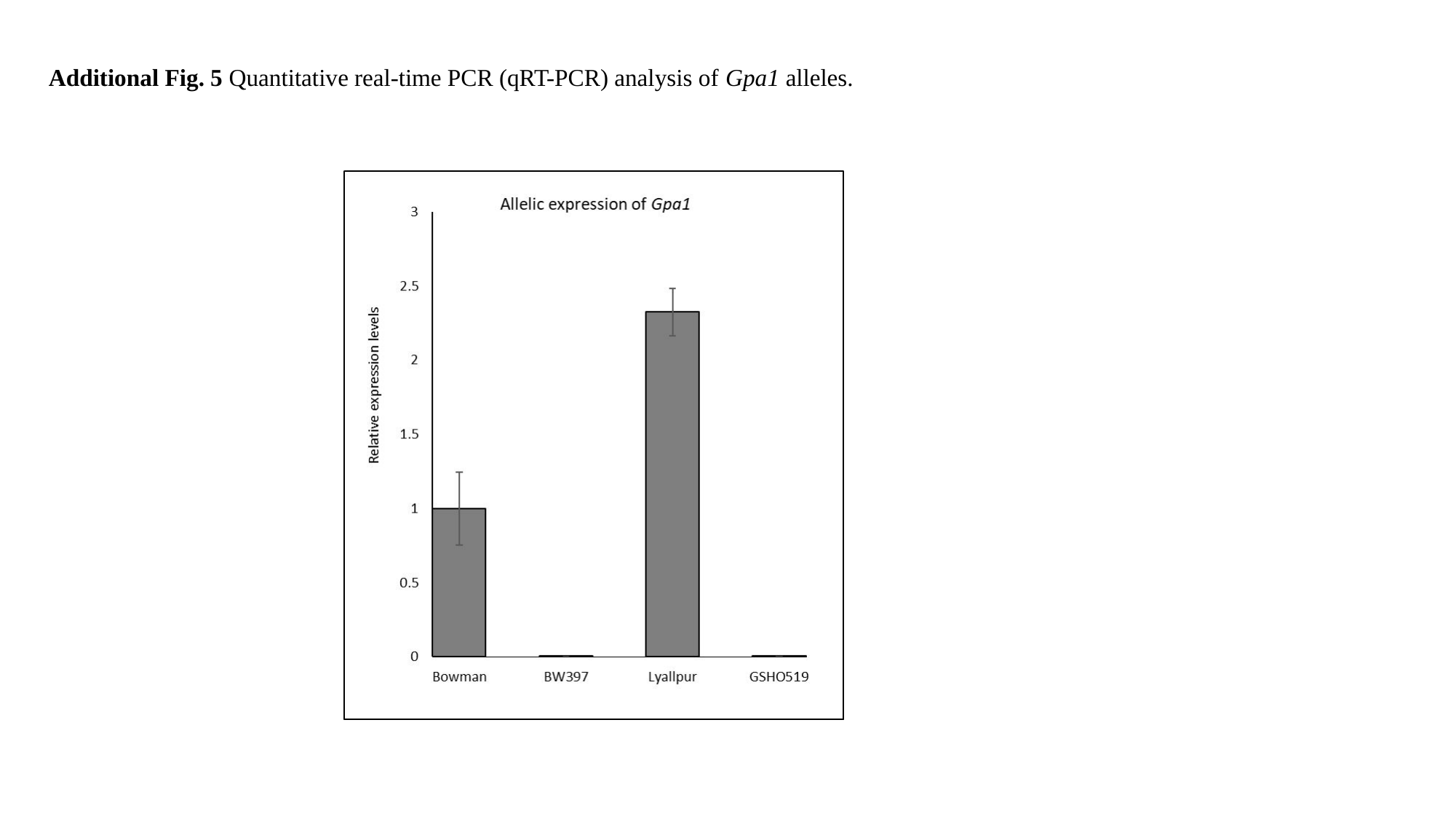

Additional Fig. 5 Quantitative real-time PCR (qRT-PCR) analysis of Gpa1 alleles.
