# Supplementary material for: Genetic analysis of the barley variegation mutant, grandpa1.a
Source: BMC Plant Biol. 2021 Mar 13;21:134. doi: 10.1186/s12870-021-02915-9 (PMC7955646; doi:10.1186/s12870-021-02915-9)
Supplement: Supplementary file 2 — Additional file 2: Table 1 SNP genotyping of 31 F2s and their parental lines with the barley 50 k iSelect SNP Array. G-1 to G-16 are F2s with normal phenotype, and g-1 to g-15 are variagated F2s. Genotypes for called SNPs were converted to the codes of pink “A”, green “B”, yellow “H” and blank “-”. The WT paent Bowman confers the pink ‘A’ genotype, “B” for the mutant BW397, “H” for heterozygous, and missing data as “-”. The flankeding SNPs, JHI-Hv50k-2016-139,629 and JHI-Hv50k-2016-142,540, were highlighted in yellow. Table 2. Primers used for the analysis of HvPTOX alleles. [file 12870_2021_2915_MOESM2_ESM.pdf]

Additional Table 1 SNP genotyping of 31 F_2s and their parental lines with the barley 50k iSelect SNP Array. G-1 to G-16 are F_2s with normal phenotype, and g-1 to g-15 are variagated F_2s. Genotypes for called SNPs were converted to the codes of pink "A", green "B", yellow "H" and blank "-". The WT paent Bowman confers the pink 'A' genotype, "B" for the mutant BW397, "H" for heterozygous, and missing data as "-". The flankeding SNPs, JHI-Hv50k-2016-139629 and JHI-Hv50k-2016-142540, were highlighted in yellow.

Index	Name	Chr	Position	Bowman	BW397	G-1	G-2	G-3	G-4	G-5	G-6	G-7	G-8	G-9	G-10	G-11	G-12	G-13	G-14	G-15	G-16	g-1	g-2	g-3	g-4	g-5	g-6	g-7	g-8	g-9	g-10	g-11	g-12	g-13	g-14	g-15
37557	JHI-Hv50k-2016-60688	2H	3273755	A	B	B	B	B	B	B	B	B	B	B	B	B	B	B	B	B	B	B	B	B	B	B	B	B	B	B	B	B	B	B	B	B
37558	JHI-Hv50k-2016-60689	2H	3273787	A	B	B	B	B	B	B	B	B	B	B	B	B	B	B	B	B	B	B	B	B	B	B	B	B	B	B	B	B	B	B	B	B
37561	JHI-Hv50k-2016-60696	2H	3275068	A	B	B	B	B	B	B	B	B	B	B	B	B	B	B	B	B	B	B	B	B	B	B	B	B	B	B	B	B	B	B	B	B
37568	JHI-Hv50k-2016-60707	2H	3275983	A	B	B	B	B	B	B	B	B	B	B	B	B	B	B	B	B	B	B	B	B	B	B	B	B	B	B	B	B	B	B	B	B
37570	JHI-Hv50k-2016-60709	2H	3276264	A	B	B	B	B	B	B	B	B	B	B	B	B	B	B	B	B	B	B	B	B	B	B	B	B	B	B	B	B	B	B	B	B
37573	JHI-Hv50k-2016-60734	2H	3325549	A	B	B	B	B	B	B	B	B	B	B	B	B	B	B	B	B	B	B	B	B	B	B	B	B	B	B	B	B	B	B	B	B
37574	JHI-Hv50k-2016-60736	2H	3325612	A	B	B	B	B	B	B	B	B	B	B	B	B	B	B	B	B	B	B	B	B	B	B	B	B	B	B	B	B	B	B	B	B
37580	JHI-Hv50k-2016-60873	2H	3581344	A	B	H	H	B	B	B	B	B	H	B	B	B	B	B	B	B	B	B	B	B	B	B	B	B	B	B	B	B	B	B	B	B
37586	JHI-Hv50k-2016-60955	2H	3728932	A	B	B	B	B	B	B	B	B	B	B	B	B	B	B	B	B	B	B	B	B	B	B	B	B	B	B	B	B	B	B	B	B
37588	JHI-Hv50k-2016-61011	2H	3737532	A	B	B	B	B	B	B	B	B	B	B	B	B	B	B	B	B	B	B	B	B	B	B	B	B	B	B	B	B	B	B	B	B
37592	JHI-Hv50k-2016-61028	2H	3740397	A	B	B	B	B	B	B	B	B	B	B	B	B	B	B	B	B	B	B	B	B	B	B	B	B	B	B	B	B	B	B	B	B
37595	JHI-Hv50k-2016-61072	2H	3746744	A	B	B	B	B	B	B	B	B	B	B	B	B	B	B	B	B	B	B	B	B	B	B	B	B	B	B	B	B	B	B	B	B
37599	JHI-Hv50k-2016-61157	2H	3990325	A	B	B	B	B	B	B	B	B	B	B	B	B	B	B	B	B	B	B	B	B	B	B	B	B	B	B	B	B	B	B	B	B
37601	JHI-Hv50k-2016-61178	2H	3994132	A	B	B	B	B	B	B	B	B	B	B	B	B	B	B	B	B	B	B	B	B	B	B	B	B	B	B	B	B	B	B	B	B
37605	JHI-Hv50k-2016-61316	2H	4240228	A	B	-	B	B	B	B	B	B	B	B	B	B	B	B	B	B	B	B	B	B	B	B	B	B	B	B	B	B	B	B	B	B
37612	JHI-Hv50k-2016-61515	2H	4427872	A	B	B	B	B	B	B	B	B	B	B	B	B	B	B	B	B	B	B	B	B	B	B	B	B	B	B	B	B	B	B	B	B
37616	JHI-Hv50k-2016-61614	2H	4547534	A	B	B	B	B	B	B	B	B	B	B	B	B	B	B	B	B	B	B	B	B	B	B	B	B	B	B	B	B	B	B	B	B
37637	JHI-Hv50k-2016-62049	2H	6191183	A	B	B	B	B	B	B	B	B	B	B	B	B	B	B	B	B	B	B	B	B	B	B	B	B	B	B	B	B	B	B	B	B
37638	JHI-Hv50k-2016-62050	2H	6191297	A	B	B	B	B	B	B	B	B	B	B	B	B	B	B	B	B	B	B	B	B	B	B	B	B	B	B	B	B	B	B	B	B
37641	JHI-Hv50k-2016-62061	2H	6192657	A	B	B	B	B	B	B	B	B	B	B	B	B	B	B	B	B	B	B	B	B	B	B	B	B	B	B	B	B	B	B	B	B
37643	JHI-Hv50k-2016-62139	2H	6223904	A	B	B	B	B	B	B	B	B	B	B	B	B	B	B	B	B	B	B	B	B	B	B	B	B	B	B	B	B	B	B	B	B
37644	JHI-Hv50k-2016-62142	2H	6224431	A	B	B	B	B	B	B	B	B	B	B	B	B	B	B	B	B	B	B	B	B	B	B	B	B	B	B	B	B	B	B	B	B
37645	JHI-Hv50k-2016-62144	2H	6224776	A	B	B	B	B	B	B	B	B	B	B	B	B	B	B	B	B	B	B	B	B	B	B	B	B	B	B	B	B	B	B	B	B
37646	JHI-Hv50k-2016-62154	2H	6225259	A	B	B	B	B	B	B	B	B	B	B	B	B	B	B	B	B	B	B	B	B	B	B	B	B	B	B	B	B	B	B	B	B
37647	JHI-Hv50k-2016-62218	2H	6247438	A	B	B	B	B	B	B	B	B	B	B	B	B	B	B	B	B	B	B	B	B	B	B	B	B	B	B	B	B	B	B	B	B
37651	JHI-Hv50k-2016-62306	2H	6427001	A	B	B	B	B	B	B	B	B	B	B	B	B	B	B	B	B	B	B	B	B	B	B	B	B	B	B	B	B	B	B	B	B
37654	JHI-Hv50k-2016-62318	2H	6428257	A	B	B	B	B	B	B	B	B	B	B	B	B	B	B	B	B	B	B	B	B	B	B	B	B	B	B	B	B	B	B	B	B
37655	JHI-Hv50k-2016-62324	2H	6428637	A	B	B	B	B	B	B	B	B	B	B	B	B	B	B	B	B	B	B	B	B	B	B	B	B	B	B	B	B	B	B	B	B
37658	JHI-Hv50k-2016-62362	2H	6432544	A	B	B	B	B	B	B	B	B	B	B	B	B	B	B	B	B	B	B	B	B	B	B	B	B	B	B	B	B	B	B	B	B
37661	JHI-Hv50k-2016-62455	2H	6753815	A	B	B	B	B	B	B	B	B	B	B	B	B	B	B	B	B	B	B	B	B	B	B	B	B	B	B	B	B	B	B	B	B
37665	JHI-Hv50k-2016-62542	2H	6957877	A	B	B	-	B	B	B	B	B	B	B	B	B	B	B	B	B	B	B	B	B	B	B	B	B	B	B	B	B	B	B	B	B
37667	JHI-Hv50k-2016-62546	2H	6958355	A	B	B	B	B	B	B	B	B	B	B	B	B	B	B	B	B	B	B	B	B	B	B	B	B	B	B	B	B	B	B	B	B
37668	JHI-Hv50k-2016-62567	2H	6961963	A	B	-	-	B	B	B	B	B	B	B	B	B	B	B	B	B	B	B	B	B	B	B	B	B	B	B	B	B	B	B	B	B
37670	JHI-Hv50k-2016-62667	2H	7267152	A	B	B	B	B	B	B	B	B	B	B	B	B	B	B	B	B	B	B	B	B	B	B	B	B	B	B	B	B	B	B	B	B
37674	JHI-Hv50k-2016-62679	2H	7269720	A	B	B	B	B	B	B	B	B	B	B	B	B	B	B	B	B	B	B	B	B	B	B	B	B	B	B	B	B	B	B	B	B
37675	JHI-Hv50k-2016-62694	2H	7273231	A	B	B	B	B	B	B	B	B	B	B	B	B	B	B	B	B	B	B	B	B	B	B	B	B	B	B	B	B	B	B	B	B
37676	JHI-Hv50k-2016-62697	2H	7273288	A	B	B	B	B	B	B	B	B	B	B	B	B	B	B	B	B	B	B	B	B	B	B	B	B	B	B	B	B	B	B	B	B
37679	JHI-Hv50k-2016-62776	2H	7351931	A	B	-	-	B	B	B	B	B	B	B	B	B	B	B	B	B	B	B	B	B	B	B	B	B	B	B	B	B	B	B	B	B
37680	JHI-Hv50k-2016-62785	2H	7352365	A	B	B	B	B	B	B	B	B	B	B	B	B	B	B	B	B	B	B	B	B	B	B	B	B	B	B	B	B	B	B	B	B
37683	JHI-Hv50k-2016-62835	2H	7489621	A	B	B	B	B	B	B	B	B	B	B	B	B	B	B	B	B	B	B	B	B	B	B	B	B	B	B	B	B	B	B	B	B
37684	JHI-Hv50k-2016-62852	2H	7490280	A	B	B	B	B	B	B	B	B	B	B	B	B	B	B	B	B	B	B	B	B	B	B	B	B	B	B	B	B	B	B	B	B
37687	JHI-Hv50k-2016-62935	2H	8035960	A	B	B	B	B	B	B	B	B	B	B	B	B	B	B	B	B	B	B	B	B	B	B	B	B	B	B	B	B	B	B	B	B
37688	JHI-Hv50k-2016-62965	2H	8042343	A	B	B	B	B	B	B	B	B	B	B	B	B	B	B	B	B	B	B	B	B	B	B	B	B	B	B	B	B	B	B	B	B
37695	JHI-Hv50k-2016-63046	2H	8304253	A	B	-	B	B	B	B	B	B	B	B	B	B	B	B	B	B	B	B	B	B	B	B	B	B	B	B	B	B	B	B	B	B
37697	JHI-Hv50k-2016-63101	2H	8470764	A	B	B	B	B	B	B	B	B	B	B	B	B	B	B	B	B	B	B	B	B	B	B	B	B	B	B	B	B	B	B	B	B
37702	JHI-Hv50k-2016-63121	2H	8536572	A	B	-	-	-	-	-	-	-	-	-	-	-	-	-	-	-	-	-	-	-	-	-	-	-	-	-	-	-	-	-	-	-
37704	JHI-Hv50k-2016-63210	2H	8696621	A	B	B	B	B	B	B	B	B	B	B	B	B	B	B	B	B	B	B	B	B	B	B	B	B	B	B	B	B	B	B	B	B
37705	JHI-Hv50k-2016-63218	2H	8697665	A	B	B	B	B	B	B	B	B	B	B	B	B	B	B	B	B	B	B	B	B	B	B	B	B	B	B	B	B	B	B	B	B
37708	JHI-Hv50k-2016-63322	2H	8953609	A	B	B	B	B	B	B	B	B	B	B	B	B	B	B	B	B	B	B	B	B	B	B	B	B	B	B	B	B	B	B	B	B
37711	JHI-Hv50k-2016-63334	2H	8955426	A	B	B	B	B	B	B	B	B	B	B	B	B	B	B	B	B	B	B	B	B	B	B	B	B	B	B	B	B	B	B	B	B
37713	JHI-Hv50k-2016-63369	2H	8996628	A	B	B	B	B	B	B	B	B	B	B	B	B	B	B	B	B	B	B	B	B	B	B	B	B	B	B	B	B	B	B	B	B
37721	JHI-Hv50k-2016-63724	2H	9344943	A	B	B	-	B	B	B	B	B	B	B	B	B	B	B	B	B	B	B	B	B	B	B	B	B	B	B	B	B	B	B	B	B
19900	JHI-Hv50k-2016-306139	5H	467839614	A	B	H	H	B	B	A	B	B	H	B	H	A	H	B	H	B	H	H	A	H	H	H	A	B	H	H	H	B	H	B	B	H
20020	JHI-Hv50k-2016-306936	5H	479117002	A	B	H	H	H	H	H	H	H	A	A	H	H	A	H	A	A	A	B	B	B	B	B	B	B	B	B	B	B	B	B	B	B
15361	JHI-Hv50k-2016-250588	4H	524036601	A	B	A	A	A	A	B	A	A	A	A	A	A	A	B	B	A	A	B	A	B	A	B	B	A	A	B	B	B	B	A	A	B
29307	JHI-Hv50k-2016-420532	6H	551826538	A	B	H	A	H	H	A	A	B	H	B	H	A	H	B	H	H	H	H	A	H	H	A	H	H	A	A	H	B	H	B	H	H
29314	JHI-Hv50k-2016-420656	6H	552076607	A	B	H	A	H	H	A	A	B	H	B	H	A	H	B	H	H	H	H	A	H	H	A	H	H	A	A	H	B	H	B	H	H
29315	JHI-Hv50k-2016-420660	6H	552076876	A	B	H	A	H	H	A	A	B	H	B	H	A	H	B	H	H	H	H	A	H	H	A	H	H	A	A	H	B	H	B	H	H
29317	JHI-Hv50k-2016-420673	6H	552078306	A	B	H	A	H	H	A	A	B	H	B	H	A	H	B	H	H	H	H	A	H	H	A	H	H	A	A	H	B	H	B	H	H
29319	JHI-Hv50k-2016-420685	6H	552078952	A	B	H	A	H	H	A	A	B	H	B	H	A	H	B	H	H	H	H	A	H	H	A	H	H	A	A	H	B	H	B	H	H
29320	JHI-Hv50k-2016-420704	6H	552081446	A	B	H	A	H	H	A	A	B	H	B	H	A	H	B	H	H	H	H	A	H	H	A	H	H	A	A	H	B	H	B	H	H
29324	JHI-Hv50k-2016-420761	6H	552246828	A	B	-	A	H	H	A	A	B	-	B	H	A	H	B	H	H	H	H	A	H	H	A	H	H	A	A	H	B	H	B	H	H
29325	JHI-Hv50k-2016-420762	6H	552246842	A	B	H	A	H	H	A	A	B	H	B	H	A	H	B	H	H	H	H	A	H	H	A	H	H	A	A	H	B	H	B	H	H
29327	JHI-Hv50k-2016-420764	6H	552247660	A	B	H	A	H	H	A	A	B	H	B	H	A	H	B	H	H	H	H	A	H	H	A	H	H	A	A	H	B	H	B	H	H
29329	JHI-Hv50k-2016-420798	6H	552354766	A	B	H	A	H	H	A	A	B	H	B	H	A	H	B	H	H	H	H	A	H	H	A	H	H	A	A	H	B	H	B	H	H
29340	JHI-Hv50k-2016-420889	6H	552734497	A	B	H	A	H	H	A	A	B	H	B	H	A	H	B	H	H	H	H	A	H	H	A	H	H	A	A	H	B	H	B	H	H
29343	JHI-Hv50k-2016-420898	6H	552736695	A	B	H	A	H	H	A	A	B	H	B	H	A	H	B	H	H	H	H	A	H	H	A	H	H	A	A	H	B	H	B	H	H
29346	JHI-Hv50k-2016-420909	6H	552764051	A	B	H	A	H	H	A	A	B	H	B	H	A	H	B	H	H	H	H	A	H	H	A	H	H	A	A	H	B	H	B	H	H
29347	JHI-Hv50k-2016-420918	6H	552765430	A	B	H	A	H	H	A	A	B	H	B	H	A	H	B	H	H	H	H	A	H	H	A	H	H	A	A	H	B	H	B	H	H
29348	JHI-Hv50k-2016-420919	6H	552765620	A	B	H	A	H	H	A	A	B	H	B	H	A	H	B	H	H	H	H	A	H	H	A	H	H	A	A	H	B	H	B	H	H
29349	JHI-Hv50k-2016-420947	6H	552805191	A	B	H	A	H	H	A	A	B	H	B	H	A	H	B	H	H	H	H	A	H	H	A	H	H	A	A	H	B	H	B	H	H
29350	JHI-Hv50k-2016-420950	6H	552805829	A	B	-	A	H	H	A	A	B	H	B	H	A	H	B	H	H	H	H	A	H	H	A	H	H	A	A	H	B	H	B	H	H
29354	JHI-Hv50k-2016-421045	6H	552899867	A	B	H	A	H	H	A	A	B	H	B	H	A	H	B	H	H	H	H	A	H	H	A	H	H	A	A	H	B	H	B	H	H
29355	JHI-Hv50k-2016-421046	6H	552899876	A	B	H	A	H	H	A	A	B	H	B	H	A	H	B	H	H	H	H	A	H	H	A	H	H	A	A	H	B	H	B	H	H
29357	JHI-Hv50k-2016-421054	6H	552901600	A	B	-	A	H	H	A	A	-	-	B	H	A	H	B	H	H	H	H	A	H	H	A	H	H	A	A	H	B	H	B	H	H
29358	JHI-Hv50k-2016-421057	6H	553002599	A	B	H	A	H	H	A	A	B	H	B	H	A	H	B	H	H	H	H	A	H	H	A	H	H	A	A	H	B	H	B	H	H
29359	JHI-Hv50k-2016-421075	6H	553018024	A	B	A	A	H	H	A	A	H	A	B	H	A	H	B	H	H	H	H	A	H	H	A	H	H	A	A	H	B	H	B	H	H
29360	JHI-Hv50k-2016-421076	6H	553018087	A	B	H	A	H	H	A	A	B	H	B	H	A	H	B	H	H	H	H	A	H	H	A	H	H	A	A	H	B	H	B	H	H
29364	JHI-Hv50k-2016-421146	6H	553264724	A	B	H	A	H	H	A	A	B	H	B	H	A	H	B	H	H	H	H	A	H	H	A	H	H	A	A	H	B	H	B	H	H
21307	JHI-Hv50k-2016-318569	5H	554665720	A	B	H	B	A	A	B	H	H	H	H	H	H	H	B	B	H	H	B	H	B	H	B	B	H	H	B	B	B	B	H	H	B
29401	JHI-Hv50k-2016-421691	6H	555704707	A	B	H	H	B	B	A	A	B	H	B	H	A	H	B	H	B	H	H	A	H	H	A	H	H	A	A	H	B	H	B	H	H
29402	JHI-Hv50k-2016-421703	6H	555717271	A	B	H	A	B	B	A	A	B	H	B	H	A	H	B	H	B	H	H	A	H	H	A	H	H	A	A	H	B	H	B	H	H
29405	JHI-Hv50k-2016-421768	6H	555819967	A	B	H	A	B	B	A	A	B	H	B	H	A	H	B	H	B	H	H	A	H	H	A	H	H	A	A	H	B	H	B	H	H
29410	JHI-Hv50k-2016-421800	6H	555830790	A	B	H	A	B	B	A	A	B	H	B	H	A	H	B	H	B	H	H	A	H	H	A	H	H	A	A	H	B	H	B	H	H
29411	JHI-Hv50k-2016-421802	6H	555831111	A	B	H	A	B	B	A	A	B	H	B	H	A	H	B	H	B	H	H	A	H	H	A	H	H	A	A	H	B	H	B	H	H
29412	JHI-Hv50k-2016-421859	6H	556041486	A	B	H	A	B	B	A	A	B	H	B	H	A	H	B	H	B	H	H	A	H	H	A	H	H	A	A	H	B	H	B	H	H
29417	JHI-Hv50k-2016-421921	6H	556303808	A	B	H	A	B	B	A	A	B	H	B	H	A	H	B	H	B	H	H	A	H	H	A	H	H	A	A	H	B	H	B	H	H
29419	JHI-Hv50k-2016-421972	6H	556373783	A	B	H	A	B	B	A	A	B	H	B	H	A	H	B	H	B	H	H	A	H	H	A	H	H	A	A	H	B	H	B	H	H
29420	JHI-Hv50k-2016-421976	6H	556374220	A	B	H	A	B	B	A	A	B	H	B	H	A	H	B	H	B	H	H	A	H	H	A	H	H	A	A	H	B	H	B	H	H
29422	JHI-Hv50k-2016-422001	6H	556379433	A	B	H	-	B	B	A	A	B	H	B	H	A	H	B	H	B	H	H	A	H	H	A	H	H	A	A	H	B	H	B	H	H
29425	JHI-Hv50k-2016-422114	6H	556680001	A	B	H	A	B	B	A	A	B	H	B	H	A	H	B	H	B	H	H	A	H	H	A	H	H	A	A	H	B	H	B	H	H
29430	JHI-Hv50k-2016-422370	6H	557445207	A	B	H	-	B	B	A	A	B	H	B	H	A	H	B	H	B	H	H	A	H	H	A	H	H	A	A	H	B	H	B	H	H
29435	JHI-Hv50k-2016-422458	6H	557711185	A	B	H	A	B	B	A	A	B	H	B	H	A	H	B	H	B	H	H	A	H	H	A	H	B	A	A	H	B	H	B	H	H
29436	JHI-Hv50k-2016-422459	6H	557711276	A	B	H	A	B	B	A	A	B	H	B	H	A	H	B	H	B	H	H	A	H	H	A	H	B	A	A	H	B	H	B	H	H
29438	JHI-Hv50k-2016-422475	6H	557776248	A	B	H	A	B	B	A	A	B	H	B	H	A	H	B	H	B	H	H	A	H	H	A	H	B	A	A	H	B	H	B	H	H
29440	JHI-Hv50k-2016-422493	6H	557854612	A	B	H	A	B	B	A	A	B	H	B	H	A	H	B	H	B	H	H	A	H	H	A	H	B	A	A	H	B	H	B	H	H
29441	JHI-Hv50k-2016-422527	6H	557892687	A	B	H	A	B	B	A	A	B	H	B	H	A	H	B	H	B	H	H	A	H	H	A	H	B	A	A	H	B	H	B	H	H
29442	JHI-Hv50k-2016-422528	6H	557892692	A	B	B	H	B	B	A	A	B	B	B	H	A	H	B	H	B	H	H	A	H	H	A	H	B	A	A	H	B	H	B	H	H

29443	JHI-Hv50k-2016-422535	6H	557893162	A	B	H	A	B	B	A	A	B	H	B	H	A	H	B	H	B	H	H	A	H	H	A	H	B	A	A	H	B	H	B	H	H
29447	JHI-Hv50k-2016-422591	6H	557943249	A	B	H	A	B	B	A	A	B	H	B	H	A	H	B	H	B	H	H	A	H	H	A	H	B	A	A	H	B	H	B	H	H
29457	JHI-Hv50k-2016-422808	6H	558862422	A	B	H	A	B	B	A	A	B	H	B	H	A	H	B	H	B	H	H	A	H	H	A	H	B	A	A	H	B	H	B	H	H
29459	JHI-Hv50k-2016-422846	6H	558879662	A	B	H	A	B	B	A	A	B	H	B	H	A	H	B	H	B	H	H	A	H	H	A	H	B	A	A	H	B	H	B	H	H
29461	JHI-Hv50k-2016-422872	6H	558898803	A	B	H	A	B	B	A	A	B	H	B	H	A	H	B	H	B	H	H	A	H	H	A	H	B	A	A	H	B	H	B	H	H
29465	JHI-Hv50k-2016-422880	6H	558900127	A	B	H	A	B	B	A	A	B	H	B	H	A	H	B	H	B	H	H	A	H	H	A	H	B	A	A	H	B	H	B	H	H
29468	JHI-Hv50k-2016-422903	6H	558902973	A	B	-	A	B	B	A	A	B	-	B	H	A	H	B	H	B	H	H	A	H	H	A	H	B	A	A	H	B	H	B	H	H
29469	JHI-Hv50k-2016-422908	6H	558904232	A	B	H	A	B	B	A	A	B	H	B	H	A	H	B	H	B	H	H	A	H	H	A	H	B	A	A	H	B	H	B	H	H
29473	JHI-Hv50k-2016-422951	6H	559351030	A	B	H	A	B	B	A	A	B	H	B	H	A	H	B	H	B	H	H	A	H	H	A	H	B	A	A	H	B	H	B	H	H
29487	JHI-Hv50k-2016-423202	6H	559840153	A	B	H	-	B	B	A	A	B	H	B	H	A	H	B	H	B	H	H	A	H	H	A	H	B	A	A	H	B	H	B	H	H
29501	JHI-Hv50k-2016-423487	6H	560055085	A	B	H	A	B	B	A	A	B	H	B	H	A	H	B	H	B	H	H	A	H	H	A	H	B	A	A	H	B	H	B	H	H
29503	JHI-Hv50k-2016-423501	6H	560159376	A	B	H	A	B	B	A	A	B	H	B	H	A	H	B	H	B	H	H	A	H	H	A	H	B	A	A	H	B	H	B	H	H
29505	JHI-Hv50k-2016-423519	6H	560435971	A	B	H	A	B	B	A	A	B	H	B	H	A	H	B	H	B	H	H	A	H	H	A	H	B	A	A	H	B	H	B	H	H
29507	JHI-Hv50k-2016-423538	6H	560439822	A	B	-	A	B	B	A	A	B	-	B	H	A	H	B	H	B	H	H	A	H	H	A	H	B	A	A	H	B	H	B	H	H
29508	JHI-Hv50k-2016-423565	6H	560541926	A	B	H	A	B	B	A	A	B	H	B	H	A	H	B	H	B	H	H	A	H	H	A	H	B	A	A	H	B	H	B	H	H
29550	JHI-Hv50k-2016-423779	6H	561583542	A	B	H	H	B	B	A	A	B	H	B	H	A	H	B	H	B	H	H	A	H	H	A	A	B	A	A	H	B	H	B	H	H
29551	JHI-Hv50k-2016-423785	6H	561584301	A	B	H	H	B	B	A	A	B	H	B	H	A	H	B	H	B	H	H	A	H	H	A	A	B	A	A	H	B	H	B	H	H
29582	JHI-Hv50k-2016-423905	6H	561742405	A	B	H	H	B	B	A	A	B	H	B	H	A	H	B	H	B	H	H	A	H	H	A	A	B	A	H	H	B	H	B	H	H
29583	JHI-Hv50k-2016-423909	6H	561742550	A	B	H	H	B	B	A	A	B	H	B	H	A	H	B	H	B	H	H	A	H	H	A	A	B	A	H	H	B	H	B	H	H
29586	JHI-Hv50k-2016-423940	6H	561761777	A	B	-	-	B	B	A	A	B	-	B	H	A	H	B	H	B	H	H	A	H	H	A	A	B	A	A	H	B	H	B	H	H
29587	JHI-Hv50k-2016-423941	6H	561761833	A	B	-	-	B	B	A	A	B	-	B	H	A	H	B	H	B	H	H	A	H	H	A	A	B	A	A	H	B	H	B	H	H
29593	JHI-Hv50k-2016-423994	6H	561807689	A	B	H	H	B	B	A	A	B	H	B	H	A	H	B	H	B	H	H	A	H	H	A	A	B	A	H	H	B	H	B	H	H
29595	JHI-Hv50k-2016-424025	6H	561832544	A	B	H	H	B	B	A	A	B	H	B	H	A	H	B	H	B	H	H	A	H	H	A	A	B	A	H	H	B	H	B	H	H
29596	JHI-Hv50k-2016-424039	6H	561880058	A	B	H	H	B	B	A	A	B	H	B	H	A	H	B	H	B	H	H	A	H	H	A	A	B	A	H	H	B	H	B	H	H
29608	JHI-Hv50k-2016-424128	6H	562088044	A	B	H	H	B	B	A	A	B	H	B	H	A	H	B	H	B	H	H	A	H	H	A	A	B	A	H	H	B	H	B	H	H
29610	JHI-Hv50k-2016-424167	6H	562232518	A	B	H	H	B	B	A	A	B	H	B	H	A	H	B	H	B	H	H	A	H	H	A	A	B	A	H	H	B	H	B	H	H
29611	JHI-Hv50k-2016-424169	6H	562232774	A	B	H	H	B	B	A	A	B	H	B	H	A	H	B	H	B	H	H	A	H	H	A	A	B	A	H	H	B	H	B	H	H
29614	JHI-Hv50k-2016-424195	6H	562349621	A	B	H	H	B	B	A	A	B	H	B	H	A	H	B	H	B	H	H	A	H	H	A	A	B	A	H	H	B	H	B	H	H
29616	JHI-Hv50k-2016-424200	6H	562681018	A	B	B	B	B	B	H	A	B	B	B	B	H	B	B	B	B	B	B	A	B	B	H	H	B	H	B	B	B	B	B	B	B
29617	JHI-Hv50k-2016-424223	6H	562713602	A	B	A	A	B	B	A	A	B	A	B	H	A	H	B	-	B	H	H	A	H	H	A	A	B	A	H	H	B	H	B	H	H
29623	JHI-Hv50k-2016-424281	6H	562812551	A	B	H	H	B	B	A	A	B	H	B	H	A	H	B	H	B	H	H	A	H	H	A	A	B	H	H	H	B	H	B	H	H
29625	JHI-Hv50k-2016-424286	6H	562812780	A	B	H	H	B	B	A	A	B	H	B	H	A	H	B	H	B	H	H	A	H	H	A	A	B	H	H	H	B	H	B	H	H
29626	JHI-Hv50k-2016-424294	6H	562813492	A	B	H	H	B	B	A	A	B	H	B	H	A	H	B	H	B	H	H	A	H	H	A	A	B	H	H	H	B	H	B	H	H
29629	JHI-Hv50k-2016-424307	6H	562815874	A	B	H	H	B	B	A	A	B	H	B	H	A	H	B	H	B	H	H	A	H	H	A	A	B	H	H	H	B	H	B	H	H
29633	JHI-Hv50k-2016-424341	6H	562861599	A	B	A	A	B	-	A	A	-	A	-	A	A	A	A	A	-	A	A	A	A	A	A	A	-	A	A	A	B	A	-	A	A
29637	JHI-Hv50k-2016-424367	6H	562864810	A	B	H	H	B	B	A	A	B	H	B	H	A	H	B	H	B	H	H	A	H	H	A	A	B	H	H	H	B	H	B	H	H
29832	JHI-Hv50k-2016-426124	6H	566296188	A	B	H	H	B	B	A	H	B	H	B	H	A	H	B	H	B	H	H	A	H	H	A	A	B	H	H	H	B	H	B	B	H
29833	JHI-Hv50k-2016-426130	6H	566296566	A	B	H	H	B	B	A	H	B	H	B	H	A	H	B	H	B	H	H	A	H	H	A	A	B	H	H	H	B	H	B	B	H
29834	JHI-Hv50k-2016-426132	6H	566296744	A	B	H	H	B	B	A	H	B	H	B	H	A	H	B	H	B	H	H	A	H	H	A	A	B	H	H	H	B	H	B	B	H
29836	JHI-Hv50k-2016-426157	6H	566410003	A	B	H	H	B	B	A	H	B	H	B	H	A	H	B	H	B	H	H	A	H	H	A	A	B	H	H	H	B	H	B	B	H
29846	JHI-Hv50k-2016-426220	6H	566446968	A	B	H	H	B	B	A	H	B	H	B	H	A	H	B	H	B	H	H	A	H	H	A	A	B	H	H	H	B	H	B	B	H
29848	JHI-Hv50k-2016-426232	6H	566449245	A	B	H	H	B	B	A	H	B	H	B	H	A	H	B	H	B	H	H	A	H	H	A	A	B	H	H	H	B	H	B	B	H
29849	JHI-Hv50k-2016-426238	6H	566450183	A	B	H	H	B	B	A	H	B	H	B	H	A	H	B	H	B	H	H	A	H	H	A	A	B	H	H	H	B	H	B	B	H
29851	JHI-Hv50k-2016-426248	6H	566452504	A	B	-	H	B	B	A	H	B	-	B	H	A	H	B	H	B	H	H	A	H	H	A	A	B	H	H	H	B	H	B	B	H
29852	JHI-Hv50k-2016-426271	6H	566540056	A	B	H	H	B	B	A	H	B	H	B	H	A	H	B	H	B	H	H	A	H	H	A	A	B	H	H	H	B	H	B	B	H
29853	JHI-Hv50k-2016-426273	6H	566540122	A	B	-	B	B	B	A	A	B	-	B	A	A	A	B	A	B	A	A	A	A	A	A	A	B	A	A	A	B	A	B	B	A
29855	JHI-Hv50k-2016-426290	6H	566731846	A	B	-	H	B	B	A	H	B	H	B	H	A	H	B	H	B	H	H	A	H	H	A	A	B	H	H	H	B	H	B	B	H
29857	JHI-Hv50k-2016-426294	6H	566738505	A	B	H	H	B	B	A	H	B	H	B	H	A	H	B	H	B	H	H	A	H	H	A	A	B	H	H	H	B	H	B	B	H
29858	JHI-Hv50k-2016-426297	6H	566738797	A	B	H	H	B	B	A	H	B	H	B	H	A	H	B	H	B	H	H	A	H	H	A	A	B	H	H	H	B	H	B	B	H
29861	JHI-Hv50k-2016-426315	6H	566837370	A	B	H	H	B	B	A	H	B	H	B	H	A	H	B	H	B	H	H	A	H	H	A	A	B	H	H	H	B	H	B	B	H
29874	JHI-Hv50k-2016-426443	6H	567207922	A	B	H	H	B	B	A	H	B	H	B	H	A	H	B	H	B	H	H	A	H	H	A	A	B	H	H	H	B	H	B	B	H
29875	JHI-Hv50k-2016-426445	6H	567207933	A	B	H	H	B	B	A	H	-	H	B	H	A	H	B	H	B	H	H	A	H	H	A	A	B	H	H	H	B	H	B	B	H
29876	JHI-Hv50k-2016-426446	6H	567208083	A	B	H	H	B	B	A	H	B	H	B	H	A	H	B	H	B	H	H	A	H	H	A	A	B	H	H	H	B	H	B	B	H
29891	JHI-Hv50k-2016-426538	6H	567505877	A	B	H	H	B	B	A	H	B	H	B	H	A	H	B	H	B	H	H	A	H	H	H	A	B	H	H	H	B	H	B	B	H
29892	JHI-Hv50k-2016-426561	6H	567509445	A	B	H	H	B	B	A	H	B	H	B	H	A	H	B	H	B	H	H	A	H	H	H	A	B	H	H	H	B	H	B	B	H
29893	JHI-Hv50k-2016-426562	6H	567509944	A	B	H	H	B	B	A	H	B	H	B	H	A	H	B	H	B	H	H	A	H	H	H	A	B	H	H	H	B	H	B	B	H
29903	JHI-Hv50k-2016-426676	6H	567652551	A	B	H	H	B	B	A	H	B	H	B	H	A	H	B	H	B	H	H	A	H	H	A	A	B	H	H	H	B	H	B	B	H
29906	JHI-Hv50k-2016-426692	6H	567911525	A	B	H	H	B	B	A	H	B	H	B	H	A	H	B	H	B	H	H	A	H	H	H	A	B	H	H	H	B	H	B	B	H
29907	JHI-Hv50k-2016-426693	6H	567911596	A	B	H	H	B	B	A	H	B	H	B	H	A	H	B	H	B	H	H	A	H	H	H	A	B	H	H	H	B	H	B	B	H
29908	JHI-Hv50k-2016-426699	6H	567912340	A	B	H	H	B	B	A	H	B	H	B	H	A	H	B	H	B	H	H	A	H	H	H	A	B	H	H	H	B	H	B	B	H
29909	JHI-Hv50k-2016-426700	6H	567912412	A	B	H	H	B	B	A	H	B	H	B	H	A	H	B	H	B	H	H	A	H	H	H	A	B	H	H	H	B	H	B	B	H
29910	JHI-Hv50k-2016-426701	6H	567912487	A	B	H	H	B	B	A	H	B	H	B	H	A	H	B	H	B	H	H	A	H	H	H	A	B	H	H	H	B	H	B	B	H
29912	JHI-Hv50k-2016-426707	6H	567912968	A	B	H	H	B	B	A	H	B	H	B	H	A	H	B	H	B	H	H	A	H	H	H	A	B	H	H	H	B	H	B	B	H
29913	JHI-Hv50k-2016-426708	6H	567913196	A	B	H	H	B	B	A	H	B	H	B	H	A	H	B	H	B	H	H	A	H	H	H	A	B	H	H	H	B	H	B	B	H
29914	JHI-Hv50k-2016-426709	6H	567913264	A	B	H	H	B	B	A	H	B	H	B	H	A	H	B	H	B	H	H	A	H	H	H	A	B	H	H	H	B	H	B	B	H
29915	JHI-Hv50k-2016-426723	6H	567927929	A	B	H	H	B	B	A	H	B	H	B	H	A	H	B	H	B	H	H	A	H	H	H	A	B	H	H	H	B	H	B	B	H
29916	JHI-Hv50k-2016-426736	6H	568038705	A	B	H	H	B	B	A	H	B	H	B	H	A	H	B	H	B	H	H	A	H	H	H	A	B	H	H	H	B	H	B	B	H
29917	JHI-Hv50k-2016-426767	6H	568179937	A	B	B	B	B	B	A	B	B	B	B	H	A	H	B	H	B	H	H	A	H	H	H	A	B	H	H	H	B	H	B	B	H
29918	JHI-Hv50k-2016-426773	6H	568224978	A	B	H	H	B	B	A	B	B	H	B	H	A	H	B	H	B	H	H	A	H	H	H	A	B	H	H	H	B	H	B	B	H
29920	JHI-Hv50k-2016-426780	6H	568228904	A	B	H	H	B	B	A	B	-	H	B	H	A	H	B	H	B	H	H	A	H	H	H	A	B	H	H	H	B	H	B	B	H
29921	JHI-Hv50k-2016-426796	6H	568231599	A	B	-	-	B	B	A	B	B	-	B	H	A	H	B	H	B	H	H	A	H	H	H	A	B	H	H	H	B	H	B	B	H
29924	JHI-Hv50k-2016-426813	6H	568247416	A	B	A	A	B	B	BB	B	B	A	B	A	BB	A	B	A	B	A	A	BB	A	A	A	BB	B	A	A	A	B	A	B	B	A
29926	JHI-Hv50k-2016-426821	6H	568277693	A	B	H	H	B	B	A	B	B	H	B	H	A	H	B	H	B	H	H	A	H	H	H	A	B	H	H	H	B	H	B	B	H
29927	JHI-Hv50k-2016-426822	6H	568278035	A	B	H	H	B	B	A	B	B	H	B	H	A	H	B	H	B	H	H	A	H	H	H	A	B	H	H	H	B	H	B	B	H
29928	JHI-Hv50k-2016-426823	6H	568280437	A	B	H	H	B	B	A	B	B	H	B	H	A	H	B	H	B	H	H	A	H	H	H	A	B	H	H	H	B	H	B	B	H
29929	JHI-Hv50k-2016-426825	6H	568281282	A	B	H	H	B	B	A	B	B	H	B	H	A	H	B	H	B	H	H	A	H	H	H	A	B	H	H	H	B	H	B	B	H
29930	JHI-Hv50k-2016-426826	6H	568281432	A	B	H	H	B	B	A	B	B	H	B	H	A	H	B	H	B	H	H	A	H	H	H	A	B	H	H	H	B	H	B	B	H
29932	JHI-Hv50k-2016-426839	6H	568302735	A	B	-	-	B	B	A	-	B	B	B	B	A	B	B	B	B	B	B	A	B	B	B	A	B	B	B	B	B	B	B	B	B
29934	JHI-Hv50k-2016-426840	6H	568302969	A	B	H	H	B	B	A	B	B	H	B	H	A	H	B	H	B	H	H	A	H	H	H	A	B	H	H	H	B	H	B	B	H
29935	JHI-Hv50k-2016-426841	6H	568303007	A	B	H	H	B	B	A	B	B	H	B	H	A	H	B	H	B	H	H	A	H	H	H	A	B	H	H	H	B	H	B	B	H
29936	JHI-Hv50k-2016-426842	6H	568303103	A	B	H	H	B	B	A	B	B	H	B	H	A	H	B	H	B	H	H	A	H	H	H	A	B	H	H	H	B	H	B	B	H
29937	JHI-Hv50k-2016-426843	6H	568303144	A	B	B	B	B	B	A	B	B	-	B	H	A	H	B	H	B	H	H	A	H	H	H	A	B	H	H	H	B	H	B	B	H
29940	JHI-Hv50k-2016-426925	6H	568605924	A	B	H	H	B	B	A	B	B	H	B	H	A	H	B	H	B	H	H	A	H	H	H	A	B	H	H	H	B	H	B	B	H
29941	JHI-Hv50k-2016-426990	6H	569738896	A	B	H	H	B	B	A	B	B	H	B	H	A	H	B	H	B	H	H	A	H	H	H	A	B	H	H	H	B	H	B	B	H
29942	JHI-Hv50k-2016-426998	6H	569743557	A	B	-	-	B	B	A	B	B	-	B	H	A	H	B	H	B	H	H	A	H	H	H	A	B	H	H	H	B	H	B	B	H
29944	JHI-Hv50k-2016-427000	6H	569743967	A	B	B	B	B	B	A	B	B	B	B	H	A	H	B	H	B	H	H	A	H	H	H	A	B	H	H	H	B	H	B	B	H
29947	JHI-Hv50k-2016-427058	6H	570019081	A	B	H	H	B	B	A	B	B	H	B	H	A	H	B	H	B	H	H	A	H	H	H	A	B	H	H	H	B	H	B	B	H
29948	JHI-Hv50k-2016-427063	6H	570020515	A	B	H	H	B	B	A	B	B	H	B	H	A	H	B	H	B	H	H	A	H	H	H	A	B	H	H	H	B	H	B	B	H
29949	JHI-Hv50k-2016-427065	6H	570020825	A	B	H	H	B	B	A	B	B	H	B	H	A	H	B	H	B	H	H	A	H	H	H	A	B	H	H	H	B	H	B	B	H
29950	JHI-Hv50k-2016-427071	6H	570053771	A	B	H	H	B	B	A	B	B	H	B	H	A	H	B	H	B	H	H	A	H	H	H	A	B	H	H	H	B	H	B	B	H
29951	JHI-Hv50k-2016-427112	6H	570137247	A	B	H	H	B	B	A	B	B	H	B	H	A	H	B	H	B	H	H	A	H	H	H	A	B	H	H	H	B	H	B	B	H
29952	JHI-Hv50k-2016-427115	6H	570137601	A	B	H	H	B	B	A	B	B	H	B	H	A	H	B	H	B	H	H	A	H	H	H	A	B	H	H	H	B	H	B	B	H
29953	JHI-Hv50k-2016-427116	6H	570137780	A	B	H	H	B	B	A	B	B	H	B	H	A	H	B	H	B	H	H	A	H	H	H	A	B	H	H	H	B	H	B	B	H
29954	JHI-Hv50k-2016-427124	6H	570170935	A	B	H	H	B	B	A	B	B	H	B	H	A	H	B	H	B	H	H	A	H	H	H	A	B	H	H	H	B	H	B	B	H
29955	JHI-Hv50k-2016-427137	6H	570201406	A	B	-	H	B	B	A	B	-	-	B	H	A	H	B	H	B	H	H	A	H	-	H	A	B	H	H	H	B	H	B	B	H
29956	JHI-Hv50k-2016-427158	6H	570261682	A	B	H	H	B	B	A	B	B	H	B	-	A	H	B	H	B	-	H	A	H	H	H	A	B	H	-	H	B	H	B	B	H
29957	JHI-Hv50k-2016-427159	6H	570261984	A	B	H	H	B	B	A	B	B	H	B	H	A	H	B	H	B	H	H	A	H	H	H	A	B	H	H	H	B	H	B	B	H
29958	JHI-Hv50k-2016-427178	6H	570406533	A	B	H	H	B	B	A	B	B	H	B	H	A	H	B	H	B	H	H	A	H	H	H	A	B	H	H	H	B	H	B	B	H
29959	JHI-Hv50k-2016-427181	6H	570408670	A	B	H	H	B	B	A	B	B	H	B	H	A	H	B	H	B	H	H	A	H	H	H	A	B	H	H	H	B	H	B	B	H
29960	JHI-Hv50k-2016-427186	6H	570411331	A	B	H	H	B	B	A	B	B	H	B	H	A	H	B	H	B	H	H	A	H	H	H	A	B	H	H	H	B	H	B	B	H
29978	JHI-Hv50k-2016-427380	6H	571356071	A	B	H	-	B	B	A	B	B	H	B	H	A	H	B	-	B	H	B	A	H	H	H	A	B	H	H	H	B	H	B	B	H
29979	JHI-Hv50k-2016-427461	6H	571366593	A	B	H	H	B	B	A	B	B	H	B	H	A	H	B	H	B	H	B	A	H	H	H	A	B	H	H	H	B	H	B	B	H
29980	JHI-Hv50k-2016-427474	6H	571367259	A	B	H	H	B	B	A	B	B	H	B	H	A	H	B	H	B	H	B	A	H	H	H	A	B	H	H	H	B	H	B	B	H
29989	JHI-Hv50k-2016-427576	6H	571419116	A	B	H	H	B	B	A	B	B	H	B	H	A	H	B	H	B	H	H	A	H	H	H	A	B	H	H	H	B	H	B	B	H
29992	JHI-Hv50k-2016-427707	6H	571458570	A	B	H	H	B	B	A	B	B	H	B	H	A	H	B	H	B	H	B	A	H	H	H	A	B	H	H	H	B	H	B	B	H
29993	JHI-Hv50k-2016-427708	6H	571458596	A	B	H	H	B	B	A	B	B	H	B	H	A	H	B	H	B	H	B	A	H	H	H	A	B	H	H	H	B	H	B	B	H
29995	JHI-Hv50k-2016-427710	6H	571458748	A	B	H	H	B	B	A	B	B	H	B	H	A	H	B	H	B	H	B	A	H	H	H	A	B	H	H	H	B	H	B	B	H

29996	JHI-Hv50k-2016-427711	6H	571458964	A	B	H	H	B	B	A	B	B	H	B	H	A	H	B	H	B	H	B	A	H	H	H	A	B	H	H	H	B	H	B	B	H
29997	JHI-Hv50k-2016-427714	6H	571459044	A	B	H	H	B	B	A	B	B	H	B	H	A	H	B	H	B	H	B	A	H	H	H	A	B	H	H	H	B	H	B	B	H
30002	JHI-Hv50k-2016-427749	6H	571737763	A	B	-	-	B	B	A	B	B	-	B	H	A	H	B	H	B	H	B	A	H	H	H	A	B	H	H	H	B	H	B	B	H
30003	JHI-Hv50k-2016-427750	6H	571737853	A	B	H	H	B	B	A	B	B	H	B	H	A	H	B	H	B	H	B	A	H	H	H	A	B	H	H	H	B	H	B	B	H
30009	JHI-Hv50k-2016-427793	6H	571848152	A	B	H	B	B	B	A	B	B	-	B	H	A	H	B	-	B	H	B	A	H	H	H	A	B	H	H	H	B	H	B	B	H
30011	JHI-Hv50k-2016-427840	6H	571851645	A	B	H	H	B	B	A	B	B	H	B	H	A	H	B	H	B	H	B	A	H	H	H	A	B	H	H	H	B	H	B	B	H
30013	JHI-Hv50k-2016-427860	6H	571852785	A	B	H	H	B	B	A	B	B	H	B	H	A	H	B	H	B	H	B	A	H	H	H	A	B	H	H	H	B	H	B	B	H
30014	JHI-Hv50k-2016-427866	6H	571853081	A	B	H	H	B	B	A	B	B	H	B	H	A	H	B	H	B	H	B	A	H	H	H	A	B	H	H	H	B	H	B	B	H
30015	JHI-Hv50k-2016-427882	6H	571854072	A	B	H	H	B	B	A	B	B	H	B	H	A	H	B	H	B	H	B	A	H	H	H	A	B	H	H	H	B	H	B	B	H
30020	JHI-Hv50k-2016-427996	6H	571876866	A	B	H	H	B	B	A	B	B	H	B	H	A	H	B	H	B	H	B	A	H	H	H	A	B	H	H	H	B	H	B	B	H
30067	JHI-Hv50k-2016-428483	6H	573491584	A	B	B	-	B	B	A	B	B	-	B	H	A	H	B	H	B	H	B	A	H	H	H	A	B	H	H	H	B	H	B	B	H
30069	JHI-Hv50k-2016-428502	6H	573494500	A	B	B	H	B	B	A	B	B	H	B	H	A	H	B	H	B	H	B	A	H	H	H	A	B	H	H	H	B	H	B	B	H
30075	JHI-Hv50k-2016-428596	6H	573554947	A	B	B	-	B	B	A	B	B	-	B	H	A	H	B	H	B	H	B	A	H	H	H	A	B	H	H	H	B	H	B	B	H
30077	JHI-Hv50k-2016-428609	6H	573557430	A	B	-	H	B	B	A	B	B	H	B	-	A	H	B	H	B	H	B	A	H	H	H	A	B	H	H	H	B	H	B	B	H
30081	JHI-Hv50k-2016-428664	6H	573640547	A	B	B	H	B	B	A	B	B	H	B	H	A	H	B	H	B	H	B	A	H	H	H	A	B	H	H	H	B	H	B	B	H
30082	JHI-Hv50k-2016-428706	6H	573659868	A	B	B	-	B	B	A	B	B	-	B	H	A	H	B	H	B	H	B	A	H	H	H	A	B	H	H	H	B	H	B	B	H
30085	JHI-Hv50k-2016-428726	6H	573681477	A	B	B	H	B	B	A	B	B	H	B	H	A	H	B	H	B	H	B	A	H	H	H	A	B	H	H	H	B	H	B	B	H
30086	JHI-Hv50k-2016-428741	6H	574032439	A	B	B	H	B	B	A	B	B	H	B	H	A	H	B	H	B	H	B	A	H	H	H	A	B	H	H	H	B	H	B	B	H
30089	JHI-Hv50k-2016-428780	6H	574041580	A	B	B	H	B	B	A	B	B	H	B	H	A	H	B	H	B	H	B	A	H	H	H	A	B	H	H	H	B	H	B	B	H
30094	JHI-Hv50k-2016-428852	6H	574297567	A	B	B	H	B	B	A	B	B	H	B	H	A	H	B	H	B	H	B	A	H	H	H	A	B	H	H	H	B	H	B	B	H
30095	JHI-Hv50k-2016-428860	6H	574298460	A	B	B	H	B	B	A	B	B	H	B	H	A	H	B	H	B	H	B	A	H	H	H	A	B	H	H	H	B	H	B	B	H
30100	JHI-Hv50k-2016-428906	6H	574783056	A	B	-	H	B	B	A	B	B	H	B	H	A	H	B	H	B	H	B	A	H	H	H	A	B	H	H	H	B	H	B	B	H
30101	JHI-Hv50k-2016-428953	6H	574797785	A	B	B	H	B	B	A	B	B	H	B	H	A	H	B	H	B	H	B	A	H	H	H	A	B	H	H	H	B	H	B	B	H
30102	JHI-Hv50k-2016-428954	6H	574797868	A	B	B	H	B	B	A	B	B	H	B	H	A	H	B	H	B	H	B	A	H	H	H	A	B	H	H	H	B	H	B	B	H
30103	JHI-Hv50k-2016-428967	6H	574798924	A	B	B	H	B	B	A	B	B	H	B	H	A	H	B	H	B	H	B	A	H	H	H	A	B	H	H	H	B	H	B	B	H
30104	JHI-Hv50k-2016-428969	6H	574798999	A	B	B	H	B	B	A	B	B	H	B	H	A	H	B	H	B	H	B	A	H	H	H	A	B	H	H	H	B	H	B	B	H
30105	JHI-Hv50k-2016-428970	6H	574799060	A	B	B	H	B	B	A	B	B	H	B	H	A	H	B	H	B	H	B	A	H	H	H	A	B	H	H	H	B	H	B	B	H
30107	JHI-Hv50k-2016-429028	6H	575038639	A	B	B	H	B	B	A	B	B	B	B	H	A	H	B	H	B	H	B	A	H	H	H	A	B	H	H	H	B	H	B	B	H
30111	JHI-Hv50k-2016-429064	6H	575043042	A	B	B	H	B	B	A	B	B	B	B	H	A	H	B	H	B	H	B	A	H	H	H	A	B	H	H	H	B	H	B	B	H
30112	JHI-Hv50k-2016-429066	6H	575043363	A	B	B	H	B	B	A	B	B	B	B	H	A	H	B	H	B	H	B	A	H	H	H	A	B	H	H	H	B	H	B	B	H
30113	JHI-Hv50k-2016-429073	6H	575043870	A	B	B	H	B	B	A	B	B	B	B	H	A	H	B	H	B	H	B	A	H	H	H	A	B	H	H	H	B	H	B	B	H
30119	JHI-Hv50k-2016-429111	6H	575071173	A	B	B	H	B	B	A	B	B	H	B	H	A	H	B	H	B	H	B	A	H	H	H	A	B	H	H	H	B	H	B	B	H
30145	JHI-Hv50k-2016-429246	6H	575694713	A	B	B	H	B	B	A	B	B	B	B	H	A	H	H	H	B	H	B	A	H	H	H	A	B	H	H	H	B	H	B	B	H
30146	JHI-Hv50k-2016-429248	6H	575694982	A	B	B	H	B	B	A	B	B	B	B	H	A	H	H	H	B	H	B	A	H	H	H	A	B	H	H	H	B	H	B	B	H
30148	JHI-Hv50k-2016-429263	6H	575725411	A	B	B	H	B	B	A	B	B	B	B	H	A	H	H	H	B	H	B	A	H	H	H	A	B	H	H	H	B	H	B	B	H
30150	JHI-Hv50k-2016-429267	6H	575727478	A	B	B	H	B	B	A	B	B	B	B	H	A	H	H	H	B	H	B	A	H	H	H	A	B	H	H	H	B	H	B	B	H
30153	JHI-Hv50k-2016-429283	6H	575771599	A	B	B	H	B	B	A	B	B	B	B	H	A	H	H	H	B	H	B	A	H	H	H	A	B	H	H	H	B	H	B	B	H
30164	JHI-Hv50k-2016-429420	6H	575956265	A	B	B	H	B	B	A	B	B	B	B	H	A	H	H	H	B	H	B	A	H	H	H	A	B	H	H	H	B	H	B	B	H
30171	JHI-Hv50k-2016-429466	6H	576405934	A	B	B	H	B	B	A	B	B	B	B	H	A	H	H	H	B	H	B	A	H	H	H	A	B	H	H	H	B	H	B	B	H
30181	JHI-Hv50k-2016-429588	6H	576456100	A	B	B	H	B	B	A	B	B	B	B	H	A	H	H	H	B	H	B	A	H	H	H	A	B	H	H	H	B	H	B	B	H
30191	JHI-Hv50k-2016-429731	6H	576572882	A	B	B	H	B	B	A	B	B	B	B	H	A	H	H	H	B	H	B	A	H	H	H	A	B	H	H	H	B	H	B	B	H
30193	JHI-Hv50k-2016-429778	6H	576590864	A	B	B	H	B	B	A	B	B	B	B	H	A	H	H	H	B	H	B	A	H	H	H	A	B	H	H	H	B	H	B	B	H
30204	JHI-Hv50k-2016-430017	6H	576878902	A	B	B	H	B	B	A	B	B	B	B	H	A	H	H	H	B	H	B	A	H	H	H	A	B	H	H	H	B	H	B	B	H
30205	JHI-Hv50k-2016-430051	6H	576920616	A	B	B	H	B	B	A	B	B	B	B	H	A	H	H	H	B	H	B	A	H	H	H	A	B	H	H	H	B	H	B	B	H
30206	JHI-Hv50k-2016-430056	6H	576922058	A	B	B	H	B	B	A	B	B	B	B	H	A	H	H	H	B	H	B	A	H	H	H	A	B	H	H	H	B	H	B	B	H
30207	JHI-Hv50k-2016-430061	6H	576928027	A	B	B	H	B	B	A	B	B	B	B	H	A	H	H	H	B	H	B	A	H	H	H	A	B	H	H	H	B	H	B	B	H
30208	JHI-Hv50k-2016-430063	6H	576951197	A	B	-	-	B	B	A	B	B	B	B	H	A	H	H	H	B	H	B	A	H	H	H	A	B	H	H	H	B	H	B	B	H
30211	JHI-Hv50k-2016-430098	6H	576953024	A	B	B	H	B	B	A	B	B	B	B	H	A	H	H	H	B	H	B	A	H	H	H	A	B	H	H	H	B	H	B	B	H
30213	JHI-Hv50k-2016-430149	6H	577100969	A	B	B	H	B	B	A	B	B	B	B	H	A	H	H	H	B	H	B	A	H	H	H	A	B	H	H	H	B	H	B	B	H
30220	JHI-Hv50k-2016-430267	6H	577459725	A	B	-	H	B	B	A	B	B	B	B	H	A	H	H	H	B	H	B	A	H	H	H	A	B	H	H	H	B	H	B	B	H
30221	JHI-Hv50k-2016-430284	6H	577460714	A	B	B	H	B	B	A	B	B	B	B	H	A	H	H	H	B	H	B	A	H	H	H	A	B	H	H	H	B	H	B	B	H
30222	JHI-Hv50k-2016-430310	6H	577493246	A	B	B	B	B	B	A	B	B	B	B	H	A	H	H	H	B	H	B	A	H	H	H	A	B	H	H	H	B	H	B	B	H
30223	JHI-Hv50k-2016-430383	6H	577508068	A	B	B	H	B	B	A	B	B	B	B	H	A	H	H	H	B	H	B	A	H	H	H	A	B	H	H	H	B	H	B	B	H
30224	JHI-Hv50k-2016-430387	6H	577508163	A	B	B	H	B	B	A	B	B	B	B	H	A	H	H	H	B	H	B	A	H	H	H	A	B	H	H	H	B	H	B	B	H
30228	JHI-Hv50k-2016-430508	6H	577602295	A	B	B	H	B	B	A	B	B	B	B	H	A	H	H	H	B	H	B	A	H	H	H	A	B	H	H	H	B	H	B	B	H
30236	JHI-Hv50k-2016-430641	6H	577769942	A	B	B	-	B	B	A	B	B	B	B	H	A	H	H	H	B	H	B	A	H	H	H	A	B	H	H	H	B	H	B	B	H
30237	JHI-Hv50k-2016-430648	6H	577774107	A	B	B	H	B	B	A	B	B	B	B	H	A	H	H	H	B	H	B	A	H	H	H	A	B	H	H	H	B	H	B	B	H
30238	JHI-Hv50k-2016-430650	6H	577774340	A	B	B	H	B	B	A	B	B	B	B	H	A	H	H	H	B	H	B	A	H	H	H	A	B	H	H	H	B	H	B	B	H
30240	JHI-Hv50k-2016-430663	6H	577775294	A	B	B	H	B	B	A	B	B	B	B	H	A	H	H	H	B	H	B	A	H	H	H	A	B	H	H	H	B	H	B	B	H
30242	JHI-Hv50k-2016-430676	6H	577775917	A	B	B	H	B	B	A	B	B	B	B	H	A	H	H	H	B	H	B	A	H	H	H	A	B	H	H	H	B	H	B	B	H
30244	JHI-Hv50k-2016-430690	6H	577788291	A	B	B	B	B	B	A	B	B	B	B	B	A	B	B	B	B	B	B	A	B	B	B	A	B	B	B	B	B	B	B	B	B
30245	JHI-Hv50k-2016-430705	6H	577828241	A	B	B	H	B	B	A	B	B	B	B	H	A	H	H	H	B	H	B	A	H	H	H	A	B	H	H	H	B	H	B	B	H
30246	JHI-Hv50k-2016-430712	6H	577828701	A	B	B	H	B	B	A	B	B	B	B	H	A	H	H	H	B	H	B	A	H	H	H	A	B	H	H	H	B	H	B	B	H
30247	JHI-Hv50k-2016-430713	6H	577828728	A	B	B	H	B	B	A	B	B	B	B	H	A	H	H	H	B	H	B	A	H	H	H	A	B	H	H	H	B	H	B	B	H
30278	JHI-Hv50k-2016-431078	6H	578692631	A	B	B	H	B	B	A	B	B	B	B	B	A	H	H	H	B	H	B	A	H	H	H	A	B	H	H	H	B	H	B	B	H
30280	JHI-Hv50k-2016-431095	6H	578720326	A	B	B	H	B	B	A	B	B	B	B	B	A	H	H	H	B	H	B	A	H	H	H	A	B	H	H	H	B	H	B	B	H
30281	JHI-Hv50k-2016-431100	6H	578720777	A	B	B	H	B	B	A	B	B	B	B	B	A	H	H	H	B	H	B	A	H	H	H	A	B	H	H	H	B	H	B	B	H
30282	JHI-Hv50k-2016-431101	6H	578720838	A	B	B	H	B	B	A	B	B	B	B	B	A	H	H	H	B	H	B	A	H	H	H	A	B	H	H	H	B	H	B	B	H
30286	JHI-Hv50k-2016-431166	6H	578732781	A	B	-	H	B	B	A	B	B	-	B	B	A	H	H	H	B	H	B	A	H	H	H	A	B	H	H	H	B	H	B	B	H
30490	JHI-Hv50k-2016-433668	6H	582585600	A	B	-	-	B	B	B	A	B	-	A	B	B	A	B	A	A	A	B	B	B	B	B	B	B	B	B	B	B	B	B	B	B
11345	JHI-Hv50k-2016-207612	3H	646976304	A	B	H	H	H	A	B	H	H	A	A	H	H	A	B	A	A	A	B	H	B	B	B	B	B	H	B	B	B	B	H	B	B
3978	JHI-Hv50k-2016-115889	2H	694597369	A	B	H	B	A	A	B	H	A	H	H	H	H	A	B	B	H	H	B	H	B	H	B	B	A	A	B	B	B	B	A	H	B
3991	JHI-Hv50k-2016-116068	2H	695090057	A	B	H	B	A	A	B	H	A	H	H	H	H	A	B	B	H	H	B	H	B	H	B	B	A	A	B	B	B	B	A	H	B
3992	JHI-Hv50k-2016-116071	2H	695091177	A	B	H	B	A	A	B	H	A	H	H	H	H	A	B	B	H	H	B	H	B	H	B	B	A	A	B	B	B	B	A	H	B
3993	JHI-Hv50k-2016-116086	2H	695303861	A	B	H	B	A	A	B	H	A	H	H	H	H	A	B	B	H	H	B	H	B	H	B	B	A	A	B	B	B	B	A	H	B
3994	JHI-Hv50k-2016-116088	2H	695304821	A	B	H	B	A	A	B	H	A	H	H	H	H	A	B	B	H	H	B	H	B	H	B	B	A	A	B	B	B	B	A	H	B
4013	JHI-Hv50k-2016-116258	2H	695896645	A	B	H	B	A	A	B	H	A	H	H	H	H	A	B	B	H	H	B	H	B	H	B	B	A	A	B	B	B	B	A	H	B
4014	JHI-Hv50k-2016-116259	2H	695896766	A	B	H	B	A	A	B	H	A	H	H	H	H	A	B	B	H	H	B	H	B	H	B	B	A	A	B	B	B	B	A	H	B
4016	JHI-Hv50k-2016-116295	2H	695906298	A	B	H	B	A	A	B	H	A	H	H	H	H	A	B	B	H	H	B	H	B	H	B	B	A	A	B	B	B	B	A	H	B
4017	JHI-Hv50k-2016-116296	2H	695906370	A	B	H	B	A	A	B	H	A	H	H	H	H	A	B	B	H	H	B	H	B	H	B	B	A	A	B	B	B	B	A	H	B
4018	JHI-Hv50k-2016-116302	2H	695906556	A	B	H	H	A	A	B	H	A	H	H	H	H	A	B	B	H	H	B	H	B	H	B	B	A	A	B	B	B	B	A	H	B
4019	JHI-Hv50k-2016-116307	2H	695906922	A	B	H	B	A	A	B	H	A	H	H	H	H	A	B	B	H	H	B	H	B	H	B	B	A	A	B	B	B	B	A	H	B
4020	JHI-Hv50k-2016-116308	2H	695906991	A	B	-	B	A	A	B	H	A	-	H	H	H	A	B	B	H	H	B	H	B	H	B	B	A	A	B	B	B	B	A	H	B
4021	JHI-Hv50k-2016-116322	2H	695909395	A	B	H	B	A	A	B	H	A	H	H	H	H	A	B	B	H	H	B	H	B	H	B	B	A	A	B	B	B	B	A	H	B
4022	JHI-Hv50k-2016-116323	2H	695909483	A	B	H	B	A	A	B	H	A	H	H	H	H	A	B	B	H	H	B	H	B	H	B	B	A	A	B	B	B	B	A	H	B
4023	JHI-Hv50k-2016-116324	2H	695909530	A	B	H	B	A	A	B	H	A	H	H	H	H	A	B	B	H	H	B	H	B	H	B	B	A	A	B	B	B	B	A	H	B
4024	JHI-Hv50k-2016-116326	2H	695909657	A	B	H	B	A	A	B	H	A	H	H	H	H	A	B	B	H	H	B	H	B	H	B	B	A	A	B	B	B	B	A	H	B
4026	JHI-Hv50k-2016-116330	2H	695909889	A	B	H	B	A	A	B	H	A	H	H	H	H	A	B	B	H	H	B	H	B	H	B	B	A	A	B	B	B	B	A	H	B
4027	JHI-Hv50k-2016-116331	2H	695909913	A	B	H	B	A	A	B	H	A	H	H	H	H	A	B	B	H	H	B	H	B	H	B	B	A	A	B	B	B	B	A	H	B
4028	JHI-Hv50k-2016-116337	2H	695911197	A	B	H	-	A	A	B	H	A	H	H	H	H	A	B	B	H	H	B	H	B	H	B	B	A	A	B	B	B	B	A	H	B
4029	JHI-Hv50k-2016-116338	2H	695911256	A	B	H	B	A	A	B	H	A	H	H	H	H	A	B	B	H	H	B	H	B	H	B	B	A	A	B	B	B	B	A	H	B
4030	JHI-Hv50k-2016-116339	2H	695911507	A	B	H	B	A	A	B	H	A	H	H	H	H	A	B	B	H	H	B	H	B	H	B	B	A	A	B	B	B	B	A	H	B
4031	JHI-Hv50k-2016-116340	2H	695911898	A	B	H	B	A	A	B	H	A	H	H	H	H	A	B	B	H	H	B	H	B	H	B	B	A	A	B	B	B	B	A	H	B
4032	JHI-Hv50k-2016-116341	2H	695911951	A	B	H	B	A	A	B	H	A	H	H	H	H	A	B	B	H	H	B	H	B	H	B	B	A	A	B	B	B	B	A	H	B
4033	JHI-Hv50k-2016-116342	2H	695912059	A	B	H	B	A	A	B	H	A	H	H	H	H	A	B	B	H	H	B	H	B	H	B	B	A	A	B	B	B	B	A	H	B
4034	JHI-Hv50k-2016-116345	2H	695912246	A	B	H	B	A	A	B	H	A	H	H	H	H	A	B	B	H	H	B	H	B	H	B	B	A	A	B	B	B	B	A	H	B
4035	JHI-Hv50k-2016-116348	2H	695912340	A	B	H	B	A	A	B	H	A	H	H	H	H	A	B	B	H	H	B	H	B	H	B	B	A	A	B	B	B	B	A	H	B
4037	JHI-Hv50k-2016-116351	2H	695912560	A	B	H	B	A	A	B	H	A	H	H	H	H	A	B	B	H	H	B	H	B	H	B	B	A	A	B	B	B	B	A	H	B
4038	JHI-Hv50k-2016-116354	2H	695912990	A	B	H	B	A	A	B	H	A	H	H	H	H	A	B	B	H	H	B	H	B	H	B	B	A	A	B	B	B	B	A	H	B
4040	JHI-Hv50k-2016-116357	2H	695913301	A	B	H	B	A	A	B	H	A	H	H	H	H	A	B	B	H	H	B	H	B	H	B	B	A	A	B	B	B	B	A	H	B
4041	JHI-Hv50k-2016-116358	2H	695913603	A	B	H	B	A	A	B	H	A	H	H	H	H	A	B	B	H	H	B	H	B	H	B	B	A	A	B	B	B	B	A	H	B
4042	JHI-Hv50k-2016-116360	2H	695913687	A	B	H	B	A	A	B	H	A	H	H	H	H	A	B	B	H	H	B	H	B	H	B	B	A	A	B	B	B	B	A	H	B
4043	JHI-Hv50k-2016-116361	2H	695913775	A	B	H	B	A	A	B	H	A	H	H	H	H	A	B	B	H	H	B	H	B	H	B	B	A	A	B	B	B	B	A	H	B
4044	JHI-Hv50k-2016-116362	2H	695913850	A	B	H	B	A	A	B	H	A	H	H	H	H	A	B	B	H	H	B	H	B	H	B	B	A	A	B	B	B	B	A	H	B
4045	JHI-Hv50k-2016-116364	2H	695913958	A	B	H	B	A	A	B	H	A	H	H	H	H	A	B	B	H	H	B	H	B	H	B	B	A	A	B	B	B	B	A	H	B
4046	JHI-Hv50k-2016-116365	2H	695914048	A	B	H	B	A	A	B	H	A	H	H	H	H	A	B	B	H	H	B	H	B	H	B	B	A	A	B	B	B	B	A	H	B
4047	JHI-Hv50k-2016-116366	2H	695914147	A	B	H	B	A	A	B	H	A	H	H	H	H	A	B	B	H	H	B	H	B	H	B	B	A	A	B	B	B	B	A	H	B

4048	JHI-Hv50k-2016-116368	2H	695914177	A	B	H	B	A	A	B	H	A	H	H	H	H	A	B	B	H	H	B	H	B	H	B	B	A	A	B	B	B	B	A	H	B
4049	JHI-Hv50k-2016-116370	2H	695914492	A	B	H	-	A	A	B	H	A	H	H	H	H	A	B	B	H	H	B	H	B	H	B	B	A	A	B	B	B	B	A	H	B
4050	JHI-Hv50k-2016-116372	2H	695914546	A	B	H	B	A	A	B	H	A	H	H	H	H	A	B	B	H	H	B	H	B	H	B	B	A	A	B	B	B	B	A	H	B
4051	JHI-Hv50k-2016-116382	2H	695915385	A	B	H	B	A	A	B	H	A	H	H	H	H	A	B	B	H	H	B	H	B	H	B	B	A	A	B	B	B	B	A	H	B
4052	JHI-Hv50k-2016-116384	2H	695915844	A	B	H	B	A	A	B	H	A	H	H	H	H	A	B	B	H	H	B	H	B	H	B	B	A	A	B	B	B	B	A	H	B
4053	JHI-Hv50k-2016-116385	2H	695915927	A	B	H	B	A	A	B	H	A	H	H	H	H	A	B	B	H	H	B	H	B	H	B	B	A	A	B	B	B	B	A	H	B
4054	JHI-Hv50k-2016-116386	2H	695916029	A	B	H	B	A	A	B	H	A	H	H	H	H	A	B	B	H	H	B	H	B	H	B	B	A	A	B	B	B	B	A	H	B
4056	JHI-Hv50k-2016-116398	2H	695916885	A	B	H	B	A	A	B	H	A	H	H	H	H	A	B	B	H	H	B	H	B	H	B	B	A	A	B	B	B	B	A	H	B
4058	JHI-Hv50k-2016-116420	2H	696110823	A	B	H	B	A	A	B	H	A	H	H	H	H	A	B	B	H	H	B	H	B	H	B	B	A	A	B	B	B	B	A	H	B
4062	JHI-Hv50k-2016-116440	2H	696117835	A	B	H	B	A	A	B	H	A	H	H	H	H	A	B	B	H	H	B	H	B	H	B	B	A	A	B	B	B	B	A	H	B
4064	JHI-Hv50k-2016-116466	2H	696239880	A	B	H	B	A	A	B	H	A	H	H	H	H	A	B	B	H	H	B	H	B	H	B	B	A	A	B	B	B	B	A	H	B
4065	JHI-Hv50k-2016-116473	2H	696240323	A	B	-	B	A	A	B	H	A	H	H	H	H	A	B	B	H	H	B	H	B	H	B	B	A	A	B	B	B	B	A	H	B
4068	JHI-Hv50k-2016-116509	2H	696276851	A	B	H	B	A	A	B	H	A	H	H	H	H	A	B	B	H	H	B	H	B	H	B	B	A	A	B	B	B	B	A	H	B
4070	JHI-Hv50k-2016-116511	2H	696276957	A	B	H	B	A	A	B	-	A	H	H	H	-	A	B	B	H	H	B	-	B	H	B	B	A	A	B	B	B	B	A	-	B
4073	JHI-Hv50k-2016-116577	2H	696377440	A	B	H	B	A	A	B	H	A	H	H	H	H	A	B	B	H	H	B	H	B	H	B	B	A	A	B	B	B	B	A	H	B
4075	JHI-Hv50k-2016-116581	2H	696377994	A	B	A	B	A	A	B	H	A	A	H	H	H	A	B	B	H	H	B	H	B	H	B	B	A	A	B	B	B	B	A	H	B
4083	JHI-Hv50k-2016-116726	2H	696711104	A	B	H	B	A	A	B	H	A	H	H	H	H	A	B	B	H	H	B	H	B	H	B	B	A	A	B	B	B	B	A	H	B
4086	JHI-Hv50k-2016-116742	2H	696714683	A	B	H	B	A	A	B	H	A	H	H	H	H	A	B	B	H	H	B	H	B	H	B	B	A	A	B	B	B	B	A	H	B
4115	JHI-Hv50k-2016-116997	2H	697332068	A	B	-	B	A	A	B	H	A	-	H	H	H	A	B	B	H	H	B	H	B	H	B	B	A	A	B	B	B	B	A	H	B
4118	JHI-Hv50k-2016-117034	2H	697738222	A	B	H	B	A	A	B	H	A	H	H	H	H	A	B	B	H	H	B	H	B	H	B	B	A	A	B	B	B	B	A	H	B
4121	JHI-Hv50k-2016-117042	2H	697863936	A	B	H	B	A	A	B	H	A	H	H	H	H	A	B	B	H	H	B	H	B	H	B	B	A	A	B	B	B	B	A	H	B
4122	JHI-Hv50k-2016-117060	2H	698145743	A	B	H	B	A	A	B	H	A	-	H	H	H	A	B	B	H	H	B	H	B	H	B	B	A	A	B	B	B	B	A	H	B
4123	JHI-Hv50k-2016-117081	2H	698149037	A	B	H	B	A	A	B	H	A	H	H	H	H	A	B	B	H	H	B	H	B	H	B	B	A	A	B	B	B	B	A	H	B
4124	JHI-Hv50k-2016-117083	2H	698149081	A	B	H	B	A	A	B	H	A	H	H	H	H	A	B	B	H	H	B	H	B	H	B	B	A	A	B	B	B	B	A	H	B
4125	JHI-Hv50k-2016-117094	2H	698165680	A	B	H	B	A	A	B	H	A	H	H	H	H	A	B	B	H	H	B	H	B	H	B	B	A	A	B	B	B	B	A	H	B
4126	JHI-Hv50k-2016-117109	2H	698220223	A	B	H	B	A	A	B	H	A	H	H	H	H	A	B	B	H	H	B	H	B	H	B	B	A	A	B	B	B	B	A	H	B
4127	JHI-Hv50k-2016-117112	2H	698220684	A	B	H	B	A	A	B	H	A	H	H	H	H	A	B	B	H	H	B	H	B	H	B	B	A	A	B	B	B	B	A	H	B
4128	JHI-Hv50k-2016-117115	2H	698221586	A	B	H	B	A	A	B	H	A	H	H	H	H	A	B	B	H	H	B	H	B	H	B	B	A	A	B	B	B	B	A	H	B
4130	JHI-Hv50k-2016-117161	2H	698231071	A	B	H	B	A	A	B	H	A	H	H	H	H	A	B	B	H	H	B	H	B	H	B	B	A	A	B	B	B	B	A	H	B
4131	JHI-Hv50k-2016-117163	2H	698231321	A	B	-	-	A	A	B	H	A	H	H	H	H	A	B	B	H	H	B	H	B	H	B	B	A	A	B	B	B	B	A	H	B
4132	JHI-Hv50k-2016-117164	2H	698231808	A	B	H	B	A	A	B	H	A	H	H	H	H	A	B	B	H	H	B	H	B	H	B	B	A	A	B	B	B	B	A	H	B
4133	JHI-Hv50k-2016-117169	2H	698314408	A	B	H	B	A	A	B	H	A	H	H	H	H	A	B	B	H	H	B	H	B	H	B	B	A	A	B	B	B	B	A	H	B
4134	JHI-Hv50k-2016-117172	2H	698314685	A	B	H	B	A	A	B	H	A	H	H	H	H	A	B	B	H	H	B	H	B	H	B	B	A	A	B	B	B	B	A	H	B
4135	JHI-Hv50k-2016-117173	2H	698314819	A	B	H	B	A	A	B	H	A	H	H	H	H	A	B	B	H	H	B	H	B	H	B	B	A	A	B	B	B	B	A	H	B
4136	JHI-Hv50k-2016-117180	2H	698441945	A	B	H	-	A	A	B	H	A	H	H	H	H	A	B	B	H	H	B	H	B	H	B	B	A	A	B	B	B	B	A	H	B
4137	JHI-Hv50k-2016-117191	2H	698446413	A	B	H	B	A	A	B	H	A	H	H	H	H	A	B	B	H	H	B	H	B	H	B	B	A	A	B	B	B	B	A	H	B
4138	JHI-Hv50k-2016-117193	2H	698446596	A	B	H	B	A	A	B	H	A	H	H	H	H	A	B	B	H	H	B	H	B	H	B	B	A	A	B	B	B	B	A	H	B
4140	JHI-Hv50k-2016-117198	2H	698617643	A	B	B	-	A	A	-	B	A	B	B	B	B	A	-	-	B	B	-	B	-	B	-	-	A	A	-	-	-	-	A	B	-
4141	JHI-Hv50k-2016-117210	2H	698668368	A	B	H	B	A	A	B	H	A	H	H	H	H	A	B	B	H	H	B	H	B	H	B	B	A	A	B	B	B	B	A	H	B
4142	JHI-Hv50k-2016-117213	2H	698668470	A	B	H	B	A	A	B	H	A	H	H	H	H	A	B	B	H	H	B	H	B	H	B	B	A	A	B	B	B	B	A	H	B
4143	JHI-Hv50k-2016-117256	2H	698732703	A	B	H	B	A	A	B	H	A	H	H	H	H	A	B	B	H	H	B	H	B	H	B	B	A	A	B	B	B	B	A	H	B
4144	JHI-Hv50k-2016-117277	2H	698876917	A	B	H	B	A	A	B	H	A	H	H	H	H	A	B	B	H	H	B	H	B	H	B	B	A	A	B	B	B	B	A	H	B
4145	JHI-Hv50k-2016-117291	2H	698901314	A	B	H	B	A	A	B	H	A	H	H	H	H	A	B	B	H	H	B	H	B	H	B	B	A	A	B	B	B	B	A	H	B
4147	JHI-Hv50k-2016-117324	2H	698913966	A	B	H	B	A	A	B	H	A	H	H	H	H	A	B	B	H	H	B	H	B	H	B	B	A	A	B	B	B	B	A	H	B
4148	JHI-Hv50k-2016-117333	2H	698915052	A	B	H	B	A	A	B	H	A	H	H	H	H	A	B	B	H	H	B	H	B	H	B	B	A	A	B	B	B	B	A	H	B
4149	JHI-Hv50k-2016-117357	2H	699045160	A	B	H	B	A	A	B	H	A	H	H	H	H	A	B	B	H	H	B	H	B	H	B	B	A	A	B	B	B	B	A	H	B
4150	JHI-Hv50k-2016-117358	2H	699045290	A	B	H	-	A	A	B	H	A	H	H	H	H	A	B	B	H	H	B	H	B	H	B	B	A	A	B	B	B	B	A	H	B
4152	JHI-Hv50k-2016-117361	2H	699045973	A	B	H	B	A	A	B	H	A	H	H	H	H	A	B	B	H	H	B	H	B	H	B	B	A	A	B	B	B	B	A	H	B
4157	JHI-Hv50k-2016-117430	2H	699224738	A	B	H	B	A	A	B	H	A	H	H	H	H	A	B	B	H	H	B	H	B	H	B	B	A	A	B	B	B	B	A	H	B
4159	JHI-Hv50k-2016-117432	2H	699225013	A	B	H	B	A	A	B	H	A	H	H	H	H	A	B	B	H	H	B	H	B	H	B	B	A	A	B	B	B	B	A	H	B
4163	JHI-Hv50k-2016-117483	2H	699323828	A	B	H	B	A	A	B	H	A	H	H	H	H	A	B	B	H	H	B	H	B	H	B	B	A	A	B	B	B	B	A	H	B
4164	JHI-Hv50k-2016-117487	2H	699323903	A	B	H	B	A	A	B	H	-	H	H	H	H	A	B	B	H	H	B	H	B	H	B	B	A	A	B	B	B	B	A	H	B
4165	JHI-Hv50k-2016-117491	2H	699324125	A	B	H	B	A	A	B	H	A	H	H	H	H	A	B	B	H	H	B	H	B	H	B	B	A	A	B	B	B	B	A	H	B
4170	JHI-Hv50k-2016-117510	2H	699438244	A	B	H	B	A	A	B	H	A	H	H	H	H	A	B	B	H	H	B	H	B	H	B	B	A	A	B	B	B	B	A	H	B
4171	JHI-Hv50k-2016-117516	2H	699438832	A	B	-	B	A	A	B	H	A	H	H	H	H	A	B	B	H	H	B	H	B	H	B	B	A	A	B	B	B	B	A	H	B
4172	JHI-Hv50k-2016-117529	2H	699445006	A	B	-	H	A	A	B	-	A	H	H	H	H	A	B	B	-	H	B	H	B	H	B	B	A	A	B	B	B	B	A	H	B
4176	JHI-Hv50k-2016-117560	2H	699753872	A	B	H	B	A	A	B	H	H	H	H	H	H	A	B	B	H	H	B	H	B	H	B	B	A	A	B	B	B	B	A	H	B
4177	JHI-Hv50k-2016-117563	2H	699755971	A	B	H	B	A	A	B	H	A	H	H	H	H	A	B	B	H	H	B	H	B	H	B	B	A	A	B	B	B	B	A	H	B
4181	JHI-Hv50k-2016-117660	2H	700117718	A	B	H	B	A	A	B	H	A	H	H	H	H	A	B	B	H	H	B	H	B	H	B	B	A	A	B	B	B	B	A	H	B
4183	JHI-Hv50k-2016-117666	2H	700118916	A	B	H	B	A	A	B	H	A	H	H	H	H	A	B	B	H	H	B	H	B	H	B	B	A	A	B	B	B	B	A	H	B
4184	JHI-Hv50k-2016-117667	2H	700119062	A	B	-	B	A	A	B	H	A	-	H	H	H	A	B	B	H	H	B	H	B	H	B	B	A	A	B	B	B	B	A	H	B
4186	JHI-Hv50k-2016-117672	2H	700119577	A	B	H	B	A	A	B	H	A	H	H	H	H	A	B	B	H	H	B	H	B	H	B	B	A	A	B	B	B	B	A	H	B
4188	JHI-Hv50k-2016-117674	2H	700119764	A	B	-	B	A	A	B	H	A	-	H	H	H	A	B	B	H	H	B	H	B	H	B	B	A	A	B	B	B	B	A	H	B
4189	JHI-Hv50k-2016-117675	2H	700119938	A	B	H	B	A	A	B	H	A	H	H	H	H	A	B	B	H	H	B	H	B	H	B	B	A	A	B	B	B	B	A	H	B
4190	JHI-Hv50k-2016-117676	2H	700120038	A	B	H	B	A	A	B	H	A	H	H	H	H	A	B	B	H	H	B	H	B	H	B	B	A	A	B	B	B	B	A	H	B
4191	JHI-Hv50k-2016-117678	2H	700120814	A	B	H	-	A	A	B	H	A	H	H	H	H	A	B	B	H	H	B	H	B	H	B	B	A	A	B	B	B	B	A	H	B
4192	JHI-Hv50k-2016-117679	2H	700120815	A	B	H	-	A	A	B	H	A	H	H	H	H	A	B	B	H	H	B	H	B	H	B	B	A	A	B	B	B	B	A	H	B
4194	JHI-Hv50k-2016-117680	2H	700120890	A	B	H	B	A	A	B	H	A	H	H	H	H	A	B	B	H	H	B	H	B	H	B	B	A	A	B	B	B	B	A	H	B
4198	JHI-Hv50k-2016-117686	2H	700121605	A	B	H	B	A	A	B	H	A	H	H	H	H	A	B	B	H	H	B	H	B	H	B	B	A	A	B	B	B	B	A	H	B
4209	JHI-Hv50k-2016-117790	2H	700214504	A	B	H	B	A	A	B	H	A	H	H	H	H	A	B	B	H	H	B	H	B	H	B	B	A	A	B	B	B	B	A	H	B
4210	JHI-Hv50k-2016-117807	2H	700216231	A	B	H	B	A	A	B	H	A	H	H	H	H	A	B	B	H	H	B	H	B	H	B	B	A	A	B	B	B	B	A	H	B
4211	JHI-Hv50k-2016-117823	2H	700217554	A	B	-	B	A	A	B	H	A	-	H	H	H	A	B	B	H	H	B	H	B	H	B	B	A	A	B	B	B	B	A	H	B
4223	JHI-Hv50k-2016-117974	2H	700397105	A	B	H	B	A	A	B	H	A	H	H	H	H	A	B	B	H	H	B	H	B	H	B	B	A	A	B	B	B	B	A	H	B
4224	JHI-Hv50k-2016-117992	2H	700558605	A	B	H	B	A	A	B	H	A	H	H	H	H	A	B	B	H	H	B	H	B	H	B	B	A	A	B	B	B	B	A	H	B
4229	JHI-Hv50k-2016-118030	2H	700563865	A	B	H	-	A	A	B	H	A	H	H	H	H	A	B	B	H	H	B	H	B	H	B	B	A	A	B	B	B	B	A	H	B
4231	JHI-Hv50k-2016-118048	2H	700567168	A	B	H	B	A	A	B	H	A	H	H	H	H	A	B	B	H	H	B	H	B	H	B	B	A	A	B	B	B	B	A	H	B
4234	JHI-Hv50k-2016-118093	2H	700725091	A	B	H	B	A	A	B	H	A	H	H	H	H	A	B	B	H	H	B	H	B	H	B	B	A	A	B	B	B	B	A	H	B
4235	JHI-Hv50k-2016-118097	2H	700725816	A	B	-	B	A	A	B	H	A	H	H	H	H	A	B	B	H	H	B	H	B	H	B	B	A	A	B	B	B	B	A	H	B
4237	JHI-Hv50k-2016-118118	2H	700739919	A	B	H	B	A	A	B	H	A	H	H	H	H	A	B	B	H	H	B	H	B	H	B	B	A	A	B	B	B	B	A	H	B
4238	JHI-Hv50k-2016-118160	2H	700870451	A	B	H	B	A	A	B	H	A	H	H	H	H	A	B	B	H	H	B	H	B	H	B	B	A	A	B	B	B	B	A	H	B
4239	JHI-Hv50k-2016-118163	2H	700872051	A	B	H	B	A	A	B	H	A	H	H	H	H	A	B	B	H	H	B	H	B	H	B	B	A	A	B	B	B	B	A	H	B
4264	JHI-Hv50k-2016-118534	2H	702365786	A	B	H	B	A	A	B	H	A	H	H	H	H	A	B	B	H	H	B	H	B	H	B	B	A	A	B	B	B	B	A	H	B
4271	JHI-Hv50k-2016-118682	2H	702956187	A	B	-	B	A	A	B	A	A	-	A	A	A	A	B	B	A	-	B	A	B	A	B	B	A	A	B	B	B	B	A	A	B
4273	JHI-Hv50k-2016-118692	2H	702956693	A	B	H	B	A	A	B	H	A	H	H	H	H	A	B	B	H	H	B	H	B	H	B	B	A	A	B	B	B	B	A	H	B
4279	JHI-Hv50k-2016-118741	2H	703221428	A	B	H	-	A	A	B	H	A	H	H	H	H	A	B	B	H	H	B	H	B	H	B	B	A	A	B	B	B	B	A	H	B
4282	JHI-Hv50k-2016-118777	2H	703319863	A	B	H	B	A	A	B	H	A	H	H	H	H	A	B	B	H	H	B	H	B	H	B	B	A	A	B	B	B	B	A	H	B
4283	JHI-Hv50k-2016-118779	2H	703319885	A	B	H	A	A	A	B	H	A	H	H	H	H	A	B	B	H	H	B	H	B	H	B	B	A	A	B	B	B	B	A	H	B
4285	JHI-Hv50k-2016-118781	2H	703320056	A	B	H	B	A	A	B	H	A	H	H	H	H	A	B	B	H	H	B	H	B	H	B	B	A	A	B	B	B	B	A	H	B
4287	JHI-Hv50k-2016-118807	2H	703356750	A	B	H	B	A	A	B	H	A	H	H	H	H	A	B	B	H	H	B	H	B	H	B	B	A	A	B	B	B	B	A	H	B
4288	JHI-Hv50k-2016-118808	2H	703356825	A	B	H	B	A	A	B	H	A	H	H	H	H	A	B	B	H	H	B	H	B	H	B	B	A	A	B	B	B	B	A	H	B
4291	JHI-Hv50k-2016-118852	2H	703645495	A	B	H	B	A	A	B	H	A	H	H	H	H	A	B	B	H	H	B	H	B	H	B	B	A	A	B	B	B	B	A	H	B
4293	JHI-Hv50k-2016-118854	2H	703645690	A	B	-	B	A	A	B	H	-	-	H	H	H	A	B	B	H	H	B	H	B	H	B	B	A	A	B	B	B	B	A	H	B
4294	JHI-Hv50k-2016-118894	2H	703710471	A	B	H	-	A	A	B	H	A	H	H	H	H	A	B	B	H	H	B	H	B	H	B	B	A	A	B	B	B	B	A	H	B
4295	JHI-Hv50k-2016-118898	2H	703739359	A	B	H	B	A	A	B	H	A	H	H	H	H	A	B	B	H	H	B	H	B	H	B	B	A	A	B	B	B	B	A	H	B
4297	JHI-Hv50k-2016-118903	2H	703925092	A	B	H	-	A	A	B	H	A	H	H	H	H	A	B	B	H	H	B	H	B	H	B	B	A	A	B	B	B	B	A	H	B
4298	JHI-Hv50k-2016-118919	2H	703931774	A	B	H	B	A	A	B	H	A	H	H	H	H	A	B	B	H	H	B	H	B	H	B	B	A	A	B	B	B	B	A	H	B
4299	JHI-Hv50k-2016-118926	2H	703932296	A	B	H	B	A	A	B	H	A	H	H	H	H	A	B	B	H	H	B	H	B	H	B	B	A	A	B	B	B	B	A	H	B
4303	JHI-Hv50k-2016-118964	2H	703950887	A	B	H	B	A	A	B	H	A	H	H	H	H	A	B	B	H	H	B	H	B	H	B	B	A	A	B	B	B	B	A	H	B
4304	JHI-Hv50k-2016-118969	2H	703952527	A	B	-	B	A	A	B	H	A	H	H	H	H	A	B	B	H	H	B	H	B	H	B	B	A	A	B	B	B	B	A	H	-
4368	JHI-Hv50k-2016-119505	2H	705263599	A	B	H	B	A	A	B	H	A	H	H	H	H	A	B	B	H	H	B	H	B	H	B	B	A	A	B	B	B	B	A	H	B
4370	JHI-Hv50k-2016-119518	2H	705364182	A	B	H	B	A	A	B	H	A	H	H	H	H	A	B	B	H	H	B	H	B	H	B	B	A	A	B	B	B	B	A	H	B
4371	JHI-Hv50k-2016-119520	2H	705364318	A	B	H	B	A	A	B	H	A	H	H	H	H	A	B	B	H	H	B	H	B	H	B	B	A	A	B	B	B	B	A	H	B
4372	JHI-Hv50k-2016-119531	2H	705593048	A	B	H	B	A	A	B	H	A	H	H	H	H	A	B	B	H	H	B	H	B	H	B	B	A	A	B	B	B	B	A	H	B
4373	JHI-Hv50k-2016-119586	2H	705628685	A	B	H	-	A	A	B	H	A	H	H	H	H	A	B	B	H	H	B	H	B	H	B	B	A	A	B	B	B	B	A	H	B
4374	JHI-Hv50k-2016-119653	2H	705704758	A	B	H	B	A	A	B	H	A	H	H	H	H	A	B	B	H	H	B	H	B	H	B	B	A	A	B	B	B	B	A	H	B
4376	JHI-Hv50k-2016-119656	2H	705705103	A	B	H	B	A	A	B	H	A	H	H	H	H	A	B	B	H	H	B	H	B	H	B	B	A	A	B	B	B	B	A	H	B
4377	JHI-Hv50k-2016-119667	2H	705791020	A	B	H	B	A	A	B	H	A	H	H	H	H	A	B	B	H	H	B	H	B	H	B	B	A	A	B	B	B	B	A	H	B

4378	JHI-Hv50k-2016-119675	2H	705821670	A	B	H	-	H	A	B	H	A	H	H	H	H	A	B	B	H	H	B	H	B	H	B	B	A	A	B	B	B	B	A	H	B
4380	JHI-Hv50k-2016-119679	2H	705822095	A	B	H	B	H	A	B	H	A	H	H	H	H	A	B	B	H	H	B	H	B	H	B	B	A	A	B	B	B	B	A	H	B
4385	JHI-Hv50k-2016-119762	2H	706344848	A	B	H	B	H	A	B	H	A	H	H	H	H	A	B	B	H	H	B	H	B	H	B	B	A	A	B	B	B	B	A	H	B
4386	JHI-Hv50k-2016-119765	2H	706345003	A	B	H	B	H	A	B	H	A	H	H	H	H	A	B	B	H	H	B	H	B	H	B	B	A	A	B	B	B	B	A	H	B
4390	JHI-Hv50k-2016-119806	2H	706349667	A	B	H	H	H	A	B	H	A	H	H	H	H	A	B	B	H	H	B	H	B	H	B	B	A	A	B	B	B	B	A	H	B
4391	JHI-Hv50k-2016-119817	2H	706350794	A	B	H	H	H	A	B	H	A	H	H	H	H	A	B	B	H	H	B	H	B	H	B	B	A	A	B	B	B	B	A	H	B
4394	JHI-Hv50k-2016-119870	2H	706465401	A	B	H	H	H	A	B	H	A	H	H	H	H	A	B	B	H	H	B	H	B	H	B	B	A	A	B	B	B	B	A	H	B
4395	JHI-Hv50k-2016-119891	2H	706466669	A	B	H	H	H	A	B	H	A	H	H	H	H	A	B	B	H	H	B	H	B	H	B	B	A	A	B	B	B	B	A	H	B
4396	JHI-Hv50k-2016-119963	2H	706471431	A	B	H	H	H	A	B	H	A	H	H	H	H	A	B	B	H	H	B	H	B	H	B	B	A	A	B	B	B	B	A	H	B
4397	JHI-Hv50k-2016-120039	2H	706577697	A	B	H	H	H	A	B	H	A	H	H	H	H	A	B	B	H	H	B	H	B	H	B	B	A	A	B	B	B	B	A	H	B
4402	JHI-Hv50k-2016-120091	2H	706918860	A	B	H	H	H	A	B	H	A	H	H	H	-	A	B	B	H	H	B	H	B	H	B	B	A	A	B	B	B	B	A	H	B
4403	JHI-Hv50k-2016-120111	2H	706924133	A	B	H	H	H	A	B	H	A	H	H	H	H	A	B	B	H	H	B	H	B	H	B	B	A	A	B	B	B	B	A	H	B
4404	JHI-Hv50k-2016-120115	2H	706965887	A	B	H	H	H	A	B	H	A	H	H	H	H	A	B	B	H	H	B	H	B	H	B	B	A	A	B	B	B	B	A	H	B
4406	JHI-Hv50k-2016-120125	2H	706966793	A	B	H	H	H	A	B	H	A	H	H	H	H	A	B	B	H	H	B	H	B	H	B	B	A	A	B	B	B	B	A	H	B
4407	JHI-Hv50k-2016-120127	2H	706966891	A	B	H	H	H	A	B	H	A	H	H	H	H	A	B	B	H	H	B	H	B	H	B	B	A	A	B	B	B	B	A	H	B
4408	JHI-Hv50k-2016-120140	2H	707024719	A	B	H	H	H	A	B	H	A	H	H	H	H	A	B	B	H	H	B	H	B	H	B	B	A	A	B	B	B	B	A	H	B
4410	JHI-Hv50k-2016-120147	2H	707025337	A	B	H	H	H	A	B	H	A	H	H	H	H	A	B	B	H	H	B	H	B	H	B	B	A	A	B	B	B	B	A	H	B
4413	JHI-Hv50k-2016-120195	2H	707266950	A	B	H	H	H	A	B	H	A	H	H	H	H	A	B	B	H	H	B	H	B	H	B	B	A	A	B	B	B	B	A	H	B
4414	JHI-Hv50k-2016-120204	2H	707313282	A	B	H	H	H	A	B	H	A	H	H	H	H	A	B	B	H	H	B	H	B	H	B	B	A	A	B	B	B	B	A	H	B
4415	JHI-Hv50k-2016-120234	2H	707380076	A	B	H	H	H	A	B	H	A	H	H	H	H	A	B	B	H	H	B	H	B	H	B	B	A	A	B	B	B	B	A	H	B
4416	JHI-Hv50k-2016-120235	2H	707380176	A	B	H	H	H	A	B	H	A	H	H	H	H	A	B	B	H	H	B	H	B	H	B	B	A	A	B	B	B	B	A	H	B
4417	JHI-Hv50k-2016-120240	2H	707380483	A	B	H	H	H	A	B	H	A	H	H	H	H	A	B	B	H	H	B	H	B	H	B	B	A	A	B	B	B	B	A	H	B
4418	JHI-Hv50k-2016-120244	2H	707380769	A	B	H	H	H	A	B	H	A	H	H	H	H	A	B	B	H	H	B	H	B	H	B	B	A	A	B	B	B	B	A	H	B
4424	JHI-Hv50k-2016-120297	2H	707543879	A	B	H	H	H	A	B	H	A	H	H	H	H	A	B	B	H	H	B	H	B	H	B	B	H	A	B	B	B	B	A	H	B
4426	JHI-Hv50k-2016-120326	2H	707561944	A	B	H	H	H	A	B	H	A	H	H	H	H	A	B	B	H	H	B	H	B	H	B	B	H	A	B	B	B	B	A	H	B
4427	JHI-Hv50k-2016-120328	2H	707562068	A	B	H	H	H	A	B	H	A	H	H	H	H	A	B	B	H	H	B	H	B	H	B	B	H	A	B	B	B	B	A	H	B
4428	JHI-Hv50k-2016-120332	2H	707563895	A	B	H	H	H	A	B	H	A	H	H	H	H	A	B	B	H	H	B	H	B	H	B	B	H	A	B	B	B	B	A	H	B
4430	JHI-Hv50k-2016-120355	2H	707586303	A	B	H	H	H	A	B	H	A	H	H	H	H	A	B	B	H	H	B	H	B	H	B	B	H	A	B	B	B	B	A	H	B
4431	JHI-Hv50k-2016-120359	2H	707587149	A	B	H	H	H	A	B	H	A	H	H	H	H	A	B	B	H	H	B	H	B	H	B	B	H	A	B	B	B	B	A	H	B
4432	JHI-Hv50k-2016-120415	2H	707593145	A	B	H	H	H	A	B	H	A	H	H	H	H	A	B	B	H	H	B	H	B	H	B	B	H	A	B	B	B	B	A	H	B
4433	JHI-Hv50k-2016-120481	2H	707642558	A	B	H	H	H	A	B	H	A	H	H	H	H	A	B	B	H	H	B	H	B	H	B	B	H	A	B	B	B	B	A	H	B
4434	JHI-Hv50k-2016-120483	2H	707642824	A	B	H	H	H	A	B	H	A	H	H	H	H	A	B	B	H	H	B	H	B	H	B	B	H	A	B	B	B	B	A	H	B
4435	JHI-Hv50k-2016-120485	2H	707643181	A	B	H	H	H	A	B	H	A	H	H	H	H	A	B	B	H	H	B	H	B	H	B	B	H	A	B	B	B	B	A	H	B
4439	JHI-Hv50k-2016-120517	2H	707900151	A	B	H	H	H	A	B	H	A	H	H	H	H	A	B	B	H	H	B	H	B	H	B	B	H	A	B	B	B	B	A	H	B
4440	JHI-Hv50k-2016-120518	2H	707901009	A	B	H	H	H	A	B	H	A	H	H	H	H	A	B	B	H	H	B	H	B	H	B	B	H	A	B	B	B	B	A	H	B
4445	JHI-Hv50k-2016-120547	2H	708177094	A	B	H	H	H	A	B	H	A	H	H	H	H	A	B	B	H	H	B	H	B	H	B	B	H	A	B	B	B	B	A	H	B
4446	JHI-Hv50k-2016-120557	2H	708178074	A	B	H	H	H	A	B	H	A	H	H	H	H	A	B	B	H	H	B	H	B	H	B	B	H	A	B	B	B	B	A	H	B
4447	JHI-Hv50k-2016-120563	2H	708178916	A	B	H	H	H	A	B	H	A	H	H	H	H	A	B	B	H	H	B	H	B	H	B	B	H	A	B	B	B	B	A	H	B
4448	JHI-Hv50k-2016-120601	2H	708242441	A	B	H	H	H	A	B	H	A	H	H	H	H	A	B	B	H	H	B	H	B	H	B	B	H	A	B	B	B	B	A	H	B
4449	JHI-Hv50k-2016-120638	2H	708253708	A	B	H	H	H	A	B	H	A	H	H	H	H	A	B	B	H	H	B	H	B	H	B	B	H	A	B	B	B	B	A	H	B
4452	JHI-Hv50k-2016-120646	2H	708254136	A	B	-	-	H	A	B	H	A	-	H	-	H	A	B	B	H	H	B	H	B	H	B	B	H	A	B	B	B	B	A	H	B
4454	JHI-Hv50k-2016-120657	2H	708264860	A	B	H	H	H	A	B	H	A	H	H	H	H	A	B	B	H	H	B	H	B	H	B	B	H	A	B	B	B	B	A	H	B
4455	JHI-Hv50k-2016-120660	2H	708265201	A	B	H	H	H	A	B	H	A	H	H	H	H	A	B	B	H	H	B	H	B	H	B	B	H	A	B	B	B	B	A	H	B
4456	JHI-Hv50k-2016-120665	2H	708297320	A	B	H	H	H	A	B	H	A	H	H	H	H	A	B	B	H	H	B	H	B	H	B	B	H	A	B	B	B	B	A	H	B
4457	JHI-Hv50k-2016-120666	2H	708297390	A	B	H	H	H	A	B	H	A	H	H	H	H	A	B	B	H	H	B	H	B	H	B	B	H	A	B	B	B	B	A	H	B
4459	JHI-Hv50k-2016-120710	2H	708562737	A	B	H	H	H	A	B	H	A	H	H	H	H	A	B	B	H	H	B	H	B	H	B	B	H	A	B	B	B	B	A	H	B
4460	JHI-Hv50k-2016-120714	2H	708562893	A	B	H	H	H	A	B	H	A	H	H	H	H	A	B	B	H	H	B	H	B	H	B	B	H	A	B	B	B	B	A	H	B
4461	JHI-Hv50k-2016-120715	2H	708562971	A	B	H	H	H	A	B	H	A	H	H	H	H	A	B	B	H	H	B	H	B	H	B	B	H	A	B	B	B	B	A	H	B
4462	JHI-Hv50k-2016-120716	2H	708563063	A	B	H	H	H	A	B	H	A	H	H	H	H	A	B	B	H	H	B	H	B	H	B	B	H	A	B	B	B	B	A	H	B
4465	JHI-Hv50k-2016-120721	2H	708564863	A	B	H	H	H	A	B	H	A	H	H	H	H	A	B	B	H	H	B	H	B	H	B	B	H	A	B	B	B	B	A	H	B
4466	JHI-Hv50k-2016-120736	2H	708568324	A	B	B	B	H	A	B	H	B	B	H	H	H	A	B	B	H	H	B	H	B	H	B	B	H	A	B	B	B	B	A	H	B
4467	JHI-Hv50k-2016-120766	2H	708637249	A	B	H	H	H	A	B	H	A	H	H	H	H	A	B	B	H	H	B	H	B	H	B	B	H	A	B	B	B	B	A	H	B
4468	JHI-Hv50k-2016-120770	2H	708638025	A	B	H	H	H	A	B	H	A	H	H	H	H	A	B	B	H	H	B	H	B	H	B	B	H	A	B	B	B	B	A	H	B
4472	JHI-Hv50k-2016-120806	2H	708849240	A	B	H	H	H	A	B	H	A	H	H	H	H	A	B	B	H	H	B	H	B	H	B	B	H	A	B	B	B	B	A	H	B
4473	JHI-Hv50k-2016-120816	2H	708860425	A	B	H	H	H	A	B	H	A	H	H	H	H	A	B	B	H	H	B	H	B	H	B	B	H	A	B	B	B	B	A	H	B
4474	JHI-Hv50k-2016-120824	2H	708863067	A	B	H	H	H	A	B	H	A	H	H	H	H	A	B	B	H	H	B	H	B	H	B	B	H	A	B	B	B	B	A	H	B
4475	JHI-Hv50k-2016-120825	2H	708863235	A	B	H	H	H	A	B	H	A	H	H	H	H	A	B	B	H	H	B	H	B	H	B	B	H	A	B	B	B	B	A	H	B
4476	JHI-Hv50k-2016-120873	2H	708968651	A	B	H	H	H	A	B	H	A	H	H	H	H	A	B	B	H	H	B	H	B	H	B	B	H	A	B	B	B	B	A	H	B
4478	JHI-Hv50k-2016-120901	2H	708993635	A	B	H	H	H	A	B	H	A	H	H	H	H	A	B	B	H	H	B	H	B	H	B	B	H	A	B	B	B	B	A	H	B
4479	JHI-Hv50k-2016-120904	2H	708993983	A	B	H	H	H	A	B	H	A	H	H	H	H	A	B	B	H	H	B	H	B	H	B	B	H	A	B	B	B	B	A	H	B
4480	JHI-Hv50k-2016-120907	2H	708994100	A	B	H	H	H	A	B	H	A	H	H	H	H	A	B	B	H	H	B	H	B	H	B	B	H	A	B	B	B	B	A	H	B
4481	JHI-Hv50k-2016-120910	2H	708994365	A	B	H	H	H	A	B	H	A	H	H	H	H	A	B	B	H	H	B	H	B	H	B	B	H	A	B	B	B	B	A	H	B
4482	JHI-Hv50k-2016-120911	2H	708994395	A	B	H	H	H	A	B	H	A	H	H	H	H	A	B	B	H	H	B	H	B	H	B	B	H	A	B	B	B	B	A	H	B
4483	JHI-Hv50k-2016-120912	2H	708994486	A	B	H	H	H	A	B	H	A	H	H	H	H	A	B	B	H	H	B	H	B	H	B	B	H	A	B	B	B	B	A	H	B
4485	JHI-Hv50k-2016-120916	2H	708994779	A	B	H	H	H	A	B	H	A	H	H	H	H	A	B	B	H	H	B	H	B	H	B	B	H	A	B	B	B	B	A	H	B
4488	JHI-Hv50k-2016-120980	2H	709120960	A	B	H	H	H	A	B	H	A	H	H	H	H	A	B	B	H	H	B	H	B	H	B	B	H	A	B	B	B	B	A	H	B
4489	JHI-Hv50k-2016-120987	2H	709122025	A	B	H	H	H	A	B	H	A	H	H	H	H	A	B	B	H	H	B	H	B	H	B	B	H	A	B	B	B	B	A	H	B
4497	JHI-Hv50k-2016-121126	2H	709326556	A	B	H	H	H	A	B	H	A	H	H	H	H	A	B	B	H	H	B	H	B	H	B	B	H	A	B	B	B	B	A	H	B
4498	JHI-Hv50k-2016-121128	2H	709326820	A	B	H	H	H	A	B	H	A	H	H	H	H	A	B	B	H	H	B	H	B	H	B	B	H	A	B	B	B	B	A	H	B
4502	JHI-Hv50k-2016-121151	2H	709349078	A	B	H	H	H	A	B	H	A	H	H	H	H	A	B	B	H	H	B	H	B	H	B	B	H	A	B	B	B	B	A	H	B
4509	JHI-Hv50k-2016-121225	2H	709535715	A	B	H	H	H	A	B	H	A	H	H	H	H	A	B	B	H	H	B	H	B	H	B	B	H	A	B	B	B	B	A	H	B
4519	JHI-Hv50k-2016-121313	2H	709631661	A	B	H	H	H	A	B	H	A	H	H	H	H	A	B	B	H	H	B	H	B	H	B	B	H	A	B	B	B	B	A	H	B
4520	JHI-Hv50k-2016-121350	2H	709856011	A	B	H	H	H	A	B	H	A	H	H	H	H	A	B	B	H	H	B	H	B	H	B	B	H	A	B	B	B	B	A	H	B
4522	JHI-Hv50k-2016-121419	2H	710066544	A	B	H	H	H	A	B	H	A	H	H	H	H	A	B	B	H	H	B	H	B	H	B	B	H	A	B	B	B	B	A	H	B
4523	JHI-Hv50k-2016-121422	2H	710084331	A	B	-	-	H	A	B	H	A	-	H	H	H	A	B	B	H	H	B	H	B	H	B	B	H	A	B	B	B	B	A	H	B
4525	JHI-Hv50k-2016-121441	2H	710148532	A	B	H	H	H	A	B	H	A	H	H	H	H	A	B	B	H	H	B	H	B	H	B	B	H	A	B	B	B	B	A	H	B
4526	JHI-Hv50k-2016-121454	2H	710162911	A	B	H	-	H	A	B	H	A	H	H	H	H	A	B	B	H	H	B	H	B	H	B	B	H	A	B	B	B	B	A	H	B
4529	JHI-Hv50k-2016-121459	2H	710163569	A	B	A	A	H	A	B	H	A	H	H	H	H	A	B	B	H	H	B	H	B	H	B	B	H	A	B	B	B	B	A	H	B
4531	JHI-Hv50k-2016-121497	2H	710182495	A	B	H	H	H	A	B	H	A	H	H	H	H	A	B	B	H	H	B	H	B	H	B	B	H	A	B	B	B	B	A	H	B
4534	JHI-Hv50k-2016-121627	2H	710600982	A	B	H	H	H	A	B	H	A	H	H	H	H	A	B	B	H	H	B	H	B	H	B	B	H	A	B	B	B	B	A	H	B
4539	JHI-Hv50k-2016-121771	2H	710747737	A	B	H	H	H	A	B	H	A	H	H	H	H	A	B	B	H	H	B	H	B	H	B	B	H	A	B	B	B	B	A	H	B
4544	JHI-Hv50k-2016-121804	2H	710842672	A	B	H	H	H	A	B	H	A	H	H	H	H	A	B	B	H	H	B	H	B	H	B	B	H	A	B	B	B	B	A	H	B
4545	JHI-Hv50k-2016-121813	2H	710843638	A	B	H	H	H	A	B	H	A	H	H	H	H	A	B	B	H	H	B	H	B	H	B	B	H	A	B	B	B	B	A	H	B
4564	JHI-Hv50k-2016-122220	2H	711116734	A	B	H	H	H	A	B	H	A	H	A	H	H	A	B	B	H	H	B	H	B	H	B	B	H	H	B	B	B	B	A	H	B
4566	JHI-Hv50k-2016-122227	2H	711118084	A	B	H	H	H	A	B	H	A	H	A	H	H	A	B	B	H	H	B	H	B	H	B	B	H	H	B	B	B	B	A	H	B
4569	JHI-Hv50k-2016-122275	2H	711164880	A	B	H	H	H	A	B	H	A	H	A	H	H	A	B	B	H	H	B	H	B	H	B	B	H	H	B	B	B	B	A	H	B
4572	JHI-Hv50k-2016-122288	2H	711226119	A	B	H	H	H	A	B	H	A	H	A	H	H	A	B	B	H	H	B	H	B	H	B	B	H	H	B	B	B	B	A	H	B
4573	JHI-Hv50k-2016-122309	2H	711230175	A	B	H	H	H	A	B	H	A	H	A	H	H	A	B	B	H	H	B	H	B	H	B	B	H	H	B	B	B	B	A	H	B
4581	JHI-Hv50k-2016-122449	2H	711656287	A	B	H	H	H	A	B	H	A	H	A	H	H	A	B	B	H	H	B	H	B	H	B	B	H	H	B	B	B	B	A	H	B
4583	JHI-Hv50k-2016-122477	2H	711748716	A	B	H	H	H	A	B	H	A	H	A	H	H	A	B	B	H	H	B	H	B	H	B	B	H	H	B	B	B	B	A	H	B
4585	JHI-Hv50k-2016-122506	2H	711778831	A	B	H	H	H	A	B	H	A	H	A	H	H	A	B	B	H	H	B	H	B	H	B	B	H	H	B	B	B	B	A	H	B
4586	JHI-Hv50k-2016-122516	2H	711779434	A	B	H	H	H	A	B	H	A	H	A	H	H	A	B	B	H	H	B	H	B	H	B	B	H	H	B	B	B	B	A	H	B
4587	JHI-Hv50k-2016-122517	2H	711779519	A	B	H	H	H	A	B	H	A	H	A	H	H	A	B	B	H	H	B	H	B	H	B	B	H	H	B	B	B	B	A	H	B
4588	JHI-Hv50k-2016-122520	2H	711779586	A	B	H	H	H	A	B	H	A	H	A	H	H	A	B	B	H	H	B	H	B	H	B	B	H	H	B	B	B	B	A	H	B
4589	JHI-Hv50k-2016-122533	2H	711786020	A	B	H	H	H	A	B	H	A	H	A	H	H	A	B	B	H	H	B	H	B	H	B	B	H	H	B	B	B	B	A	H	B
4590	JHI-Hv50k-2016-122549	2H	712178173	A	B	H	H	H	A	B	H	A	H	A	H	H	A	B	B	H	H	B	H	B	H	B	B	H	H	B	B	B	B	A	H	B
4591	JHI-Hv50k-2016-122550	2H	712178275	A	B	H	H	H	A	B	H	A	H	A	H	H	A	B	B	H	H	B	H	B	H	B	B	H	H	B	B	B	B	A	H	B
4592	JHI-Hv50k-2016-122563	2H	712194673	A	B	A	A	H	A	B	H	A	A	A	H	H	A	B	B	H	H	B	H	B	H	B	B	H	H	B	B	B	B	A	H	B
4594	JHI-Hv50k-2016-122570	2H	712195435	A	B	H	H	H	A	B	H	A	H	A	H	H	A	B	B	H	H	B	H	B	H	B	B	H	H	B	B	B	B	A	H	B
4598	JHI-Hv50k-2016-122691	2H	712312893	A	B	H	H	H	A	B	H	A	-	A	H	H	A	B	B	H	H	B	H	B	H	B	B	H	H	B	B	B	B	A	H	B
4599	JHI-Hv50k-2016-122699	2H	712330524	A	B	-	-	H	A	B	H	A	-	A	H	H	A	B	B	H	H	B	H	B	H	B	B	H	H	B	B	B	B	A	H	B
4600	JHI-Hv50k-2016-122707	2H	712331427	A	B	H	H	H	A	B	H	A	H	A	H	H	A	B	B	H	H	B	H	B	H	B	B	H	H	B	B	B	B	A	H	B
4603	JHI-Hv50k-2016-122729	2H	712501396	A	B	-	-	H	A	B	H	A	-	A	H	H	A	B	B	H	H	B	H	B	H	B	B	H	H	B	B	B	B	A	H	B
4608	JHI-Hv50k-2016-122806	2H	712676153	A	B	H	H	H	A	B	H	A	H	A	H	H	A	B	B	H	H	B	H	B	H	B	B	H	H	B	B	B	B	A	H	B
4610	JHI-Hv50k-2016-122831	2H	712754147	A	B	H	H	H	A	B	H	A	H	A	H	H	A	B	B	H	H	B	H	B	H	B	B	H	H	B	B	B	B	A	H	B
4611	JHI-Hv50k-2016-122843	2H	712797520	A	B	H	H	H	A	B	H	A	H	A	H	H	A	B	H	H	H	B	H	B	H	B	B	H	H	B	B	B	B	A	B	B
4615	JHI-Hv50k-2016-122885	2H	712830796	A	B	H	H	H	A	B	H	A	H	A	H	H	A	B	H	H	H	B	H	B	H	B	B	H	H	B	B	B	B	A	B	B

4618	JHI-Hv50k-2016-122998	2H	713067811	A	B	H	H	H	A	B	H	A	H	A	H	H	A	B	H	H	H	B	H	B	H	B	B	H	H	B	B	B	B	A	B	B
4619	JHI-Hv50k-2016-123005	2H	713068606	A	B	H	H	H	A	B	H	A	H	A	H	H	A	B	H	H	H	B	H	B	H	B	B	H	H	B	B	B	B	A	B	B
4623	JHI-Hv50k-2016-123148	2H	713112775	A	B	H	H	H	A	B	H	A	H	A	H	H	A	B	H	H	H	B	H	B	H	B	B	H	H	B	B	B	B	A	B	B
4626	JHI-Hv50k-2016-123263	2H	713241243	A	B	H	H	H	A	B	H	A	H	A	H	H	A	B	H	H	H	B	H	B	H	B	B	H	H	B	B	B	B	A	B	B
4627	JHI-Hv50k-2016-123277	2H	713242767	A	B	H	-	H	A	B	H	A	H	A	H	H	A	B	H	H	H	B	H	B	H	B	B	H	H	B	B	B	B	A	B	B
4628	JHI-Hv50k-2016-123289	2H	713244177	A	B	H	H	H	A	B	H	-	H	A	H	H	A	B	H	H	H	B	H	B	H	B	B	H	H	B	B	B	B	A	B	B
4632	JHI-Hv50k-2016-123547	2H	713704109	A	B	H	H	H	A	B	H	A	H	A	H	H	A	B	H	H	H	B	H	B	H	B	B	H	H	B	B	B	B	A	B	B
4634	JHI-Hv50k-2016-123572	2H	713705537	A	B	H	H	H	A	B	H	A	H	A	H	H	A	B	H	H	H	B	H	B	H	B	B	H	H	B	B	B	B	A	B	B
4635	JHI-Hv50k-2016-123575	2H	713705899	A	B	H	H	H	A	B	H	A	H	A	H	H	A	B	H	H	H	B	H	B	H	B	B	H	H	B	B	B	B	A	B	B
4636	JHI-Hv50k-2016-123576	2H	713706115	A	B	H	H	H	A	B	H	A	H	A	H	H	A	B	H	H	H	B	H	B	H	B	B	H	H	B	B	B	B	A	B	B
4638	JHI-Hv50k-2016-123652	2H	713744262	A	B	H	H	H	A	B	H	A	H	A	H	H	A	B	H	H	H	B	H	B	H	B	B	H	H	B	B	B	B	A	B	B
4640	JHI-Hv50k-2016-123656	2H	713744449	A	B	H	H	H	A	B	H	A	H	A	H	H	A	B	H	H	H	B	H	B	H	B	B	H	H	B	B	B	B	A	B	B
4642	JHI-Hv50k-2016-123719	2H	713774679	A	B	H	H	H	A	B	H	A	H	A	H	H	A	B	H	H	H	B	H	B	H	B	B	H	H	B	B	B	B	A	B	B
4644	JHI-Hv50k-2016-123739	2H	713775521	A	B	H	H	H	A	B	H	A	H	A	H	H	A	B	H	H	H	B	H	B	H	B	B	H	H	B	B	B	B	A	B	B
4645	JHI-Hv50k-2016-123752	2H	713776257	A	B	H	H	H	A	B	H	A	H	A	H	H	A	B	H	H	H	B	H	B	H	B	B	H	H	B	B	B	B	A	B	B
4646	JHI-Hv50k-2016-123834	2H	713820081	A	B	H	H	H	A	B	H	A	H	A	H	H	A	B	H	H	H	B	H	B	H	B	B	H	H	B	B	B	B	A	B	B
4648	JHI-Hv50k-2016-123879	2H	713827336	A	B	H	H	H	A	B	H	A	H	A	H	H	A	B	H	H	H	B	H	B	H	B	B	H	H	B	B	B	B	A	B	B
4649	JHI-Hv50k-2016-123897	2H	713834634	A	B	H	H	H	A	B	H	A	H	A	H	H	A	B	H	H	H	B	H	B	H	B	B	H	H	B	B	B	B	A	B	B
4651	JHI-Hv50k-2016-123949	2H	713936402	A	B	H	H	H	A	B	H	A	H	A	H	H	A	B	H	H	H	B	H	B	H	B	B	H	H	B	B	B	B	A	B	B
4653	JHI-Hv50k-2016-124029	2H	714019955	A	B	-	A	H	A	B	H	H	H	A	H	H	A	B	H	H	H	B	H	B	H	B	B	H	H	B	B	B	B	A	B	B
4654	JHI-Hv50k-2016-124035	2H	714020215	A	B	H	H	H	A	B	H	H	H	A	H	H	A	B	H	H	H	B	H	B	H	B	B	H	H	B	B	B	B	A	B	B
4676	JHI-Hv50k-2016-124316	2H	715074726	A	B	H	H	H	A	B	H	H	H	A	H	H	A	B	H	H	H	B	H	B	H	B	B	H	H	B	B	B	B	A	B	B
4680	JHI-Hv50k-2016-124342	2H	715157930	A	B	H	H	H	A	B	H	H	H	A	H	H	A	B	H	H	H	B	H	B	H	B	B	H	H	B	B	B	B	A	B	B
4681	JHI-Hv50k-2016-124347	2H	715160155	A	B	H	H	H	A	B	H	H	H	A	H	H	A	B	H	H	H	B	H	B	H	B	B	H	H	B	B	B	B	A	B	B
4682	JHI-Hv50k-2016-124349	2H	715160995	A	B	-	-	H	A	B	H	H	H	A	H	H	A	B	H	H	H	B	H	B	H	B	B	H	H	B	B	B	B	A	B	B
4696	JHI-Hv50k-2016-124483	2H	715795276	A	B	H	H	H	A	B	H	H	H	A	H	H	A	B	H	H	H	B	H	B	H	B	B	H	H	B	B	B	B	A	B	B
4697	JHI-Hv50k-2016-124484	2H	715795335	A	B	H	H	H	A	B	H	H	H	A	H	H	A	B	H	H	H	B	H	B	H	B	B	H	H	B	B	B	B	A	B	B
4698	JHI-Hv50k-2016-124491	2H	715917424	A	B	H	H	H	A	B	H	H	H	A	H	H	A	B	H	H	H	B	H	B	H	B	B	H	H	B	B	B	B	A	B	B
4699	JHI-Hv50k-2016-124521	2H	715920425	A	B	H	H	H	A	B	H	H	H	A	H	H	A	B	H	H	H	B	H	B	H	B	B	H	H	B	B	B	B	A	B	B
4700	JHI-Hv50k-2016-124532	2H	715920707	A	B	H	H	H	A	B	H	H	H	A	H	H	A	B	H	H	H	B	H	B	H	B	B	H	H	B	B	B	B	A	B	B
4702	JHI-Hv50k-2016-124551	2H	715925315	A	B	H	H	H	A	B	H	H	H	A	H	H	A	B	H	H	H	B	H	B	H	B	B	H	H	B	B	B	B	A	B	B
4703	JHI-Hv50k-2016-124562	2H	715933490	A	B	H	H	H	A	B	H	H	H	A	H	H	A	B	H	H	H	B	H	B	H	B	B	H	H	B	B	B	B	A	B	B
4704	JHI-Hv50k-2016-124571	2H	715935074	A	B	H	H	H	A	B	H	H	H	A	H	H	A	B	H	H	H	B	H	B	H	B	B	H	H	B	B	B	B	A	B	B
4705	JHI-Hv50k-2016-124572	2H	715935412	A	B	H	H	H	A	B	H	H	H	A	H	H	A	B	H	H	H	B	H	B	H	B	B	H	H	B	B	B	B	A	B	B
4706	JHI-Hv50k-2016-124604	2H	715950439	A	B	H	H	H	A	B	H	H	H	A	H	H	A	B	H	H	H	B	H	B	H	B	B	H	H	B	B	B	B	A	B	B
4707	JHI-Hv50k-2016-124612	2H	715951234	A	B	H	H	H	A	B	H	H	H	A	H	H	A	B	H	H	H	B	H	B	H	B	B	H	H	B	B	B	B	A	B	B
4710	JHI-Hv50k-2016-124640	2H	716128315	A	B	H	H	H	A	B	H	H	H	A	H	H	A	B	H	H	H	B	H	B	H	B	B	H	H	B	B	B	B	A	B	B
4712	JHI-Hv50k-2016-124830	2H	716364517	A	B	H	H	H	A	B	H	H	H	A	H	H	A	B	H	H	H	B	H	B	H	B	B	H	H	B	B	B	B	A	B	B
4713	JHI-Hv50k-2016-124833	2H	716364887	A	B	H	H	H	A	B	H	H	H	A	H	H	A	B	H	H	H	B	H	B	H	B	B	H	H	B	B	B	B	A	B	B
4714	JHI-Hv50k-2016-124850	2H	716369087	A	B	H	H	H	A	B	H	H	H	A	H	H	A	B	H	H	H	B	H	B	H	B	B	H	H	B	B	B	B	A	B	B
4715	JHI-Hv50k-2016-124888	2H	716478426	A	B	H	H	H	A	B	H	H	H	A	H	H	A	B	H	H	H	B	H	B	H	B	B	H	H	B	B	B	B	A	B	B
4717	JHI-Hv50k-2016-124924	2H	716482621	A	B	H	H	H	A	B	H	H	H	A	H	H	A	B	H	H	H	B	H	B	H	B	B	H	H	B	B	B	B	A	B	B
4718	JHI-Hv50k-2016-124927	2H	716482753	A	B	H	H	H	A	B	H	H	H	A	H	H	A	B	H	H	H	B	H	B	H	B	B	H	H	B	B	B	B	A	B	B
4719	JHI-Hv50k-2016-124930	2H	716482911	A	B	H	H	H	A	B	H	H	H	A	H	H	A	B	H	H	H	B	H	B	H	B	B	H	H	B	B	B	B	A	B	B
4720	JHI-Hv50k-2016-124972	2H	716589500	A	B	H	H	H	A	B	H	H	H	A	H	H	A	B	H	H	H	B	H	B	H	B	B	H	H	B	B	B	B	A	B	B
4721	JHI-Hv50k-2016-124974	2H	716589685	A	B	H	H	H	A	B	H	H	H	A	H	H	A	B	H	H	H	B	H	B	H	B	B	H	H	B	B	B	B	A	B	B
4722	JHI-Hv50k-2016-125036	2H	716823146	A	B	H	H	H	A	B	H	H	H	A	H	H	A	B	H	H	H	B	H	B	H	B	B	H	H	B	B	B	B	A	B	B
4723	JHI-Hv50k-2016-125068	2H	716825008	A	B	H	H	H	A	B	H	H	H	A	H	H	A	B	H	H	H	B	H	B	H	B	B	H	H	B	B	B	B	A	B	B
4726	JHI-Hv50k-2016-125083	2H	716825943	A	B	H	H	H	A	B	H	H	H	A	H	H	A	B	H	H	H	B	H	B	H	B	B	H	H	B	B	B	B	A	B	B
4727	JHI-Hv50k-2016-125086	2H	716826139	A	B	H	H	H	A	B	H	H	H	A	H	H	A	B	H	H	H	B	H	B	H	B	B	H	H	B	B	B	B	A	B	B
4734	JHI-Hv50k-2016-125295	2H	717372118	A	B	H	H	H	A	B	H	H	H	A	H	H	A	B	H	H	H	B	H	B	H	B	B	H	H	B	B	B	B	A	B	B
4735	JHI-Hv50k-2016-125298	2H	717372509	A	B	H	H	H	A	B	H	H	H	A	H	H	A	B	H	H	H	B	H	B	H	B	B	H	H	B	B	B	B	A	B	B
4736	JHI-Hv50k-2016-125308	2H	717373243	A	B	H	H	H	A	B	H	H	H	A	H	H	A	B	H	H	H	B	H	B	H	B	B	H	H	B	B	B	B	A	B	B
4737	JHI-Hv50k-2016-125310	2H	717373251	A	B	H	H	H	A	B	H	H	H	A	H	H	A	B	H	H	H	B	H	B	H	B	B	H	H	B	B	B	B	A	B	B
4740	JHI-Hv50k-2016-125359	2H	717611197	A	B	H	H	H	A	B	H	H	H	A	H	H	A	B	H	H	A	B	H	B	H	B	B	H	H	B	B	B	B	A	B	B
4741	JHI-Hv50k-2016-125366	2H	717611446	A	B	H	H	H	A	B	H	H	H	A	H	H	A	B	H	H	A	B	H	B	H	B	B	H	H	B	B	B	B	A	B	B
4745	JHI-Hv50k-2016-125433	2H	717873016	A	B	H	H	H	A	B	H	H	H	A	H	H	A	B	H	H	A	B	H	B	H	B	B	H	H	B	B	B	B	A	B	B
4747	JHI-Hv50k-2016-125449	2H	717878049	A	B	H	H	H	A	B	H	H	H	A	H	H	A	B	H	H	A	B	H	B	H	B	B	H	H	B	B	B	B	A	B	B
4748	JHI-Hv50k-2016-125451	2H	717878163	A	B	A	A	-	A	B	A	-	A	A	A	A	-	B	A	A	A	B	A	B	A	B	B	A	A	B	B	B	B	-	B	B
4754	JHI-Hv50k-2016-125681	2H	718155005	A	B	H	H	H	A	B	H	H	H	A	H	H	A	B	H	H	A	B	H	B	H	B	B	H	H	B	B	B	B	A	B	B
4755	JHI-Hv50k-2016-125682	2H	718155064	A	B	H	H	H	A	B	H	H	H	A	H	H	A	B	H	H	A	B	H	B	H	B	B	H	H	B	B	B	B	A	B	B
4757	JHI-Hv50k-2016-125733	2H	718209681	A	B	H	H	H	A	B	H	H	H	A	H	H	A	B	H	H	A	B	H	B	H	B	B	H	H	B	B	B	B	A	B	B
4759	JHI-Hv50k-2016-125741	2H	718211159	A	B	H	H	H	A	B	H	H	H	A	H	H	A	B	H	H	A	B	H	B	H	B	B	H	H	B	B	B	B	A	B	B
4760	JHI-Hv50k-2016-125747	2H	718212221	A	B	H	H	H	A	B	H	H	H	A	H	H	A	B	H	H	A	B	H	B	H	B	B	H	H	B	B	B	B	A	B	B
4761	JHI-Hv50k-2016-125766	2H	718334575	A	B	H	H	H	A	B	H	H	H	A	H	H	A	B	H	H	A	B	H	B	H	B	B	H	H	B	B	B	B	A	B	B
4764	JHI-Hv50k-2016-125822	2H	718431387	A	B	H	H	H	A	B	H	H	H	A	H	H	A	B	H	H	A	B	H	B	H	B	B	H	H	B	B	B	B	A	B	B
4765	JHI-Hv50k-2016-125845	2H	718434772	A	B	H	H	H	A	B	H	H	H	A	H	H	A	B	H	H	A	B	H	B	H	B	B	H	H	B	B	B	B	A	B	B
4766	JHI-Hv50k-2016-125851	2H	718435284	A	B	H	H	H	A	B	H	H	H	A	H	H	A	B	H	H	A	B	H	B	H	B	B	H	H	B	B	B	B	A	B	B
4767	JHI-Hv50k-2016-125852	2H	718435345	A	B	H	H	H	A	B	H	H	H	A	H	H	A	B	H	H	A	B	H	B	H	B	B	H	H	B	B	B	B	A	B	B
4769	JHI-Hv50k-2016-125904	2H	718483583	A	B	H	H	H	A	B	H	H	H	A	H	H	A	B	H	H	A	B	H	B	H	B	B	H	H	B	B	B	B	A	B	B
4770	JHI-Hv50k-2016-125905	2H	718483618	A	B	H	H	H	A	B	H	H	H	A	H	H	A	B	H	H	A	B	H	B	H	B	B	H	H	B	B	B	B	A	B	B
4771	JHI-Hv50k-2016-125911	2H	718585587	A	B	H	H	H	A	B	H	H	H	A	H	H	A	B	H	H	A	B	H	B	H	B	B	H	H	B	B	B	B	A	B	B
4772	JHI-Hv50k-2016-125914	2H	718586125	A	B	H	H	H	A	B	H	H	H	A	H	H	A	B	H	H	A	B	H	B	H	B	B	H	H	B	B	B	B	A	B	B
4773	JHI-Hv50k-2016-125918	2H	718588669	A	B	H	H	H	A	B	H	H	H	A	H	H	A	B	H	H	A	B	H	B	H	B	B	H	H	B	B	B	B	A	B	B
4931	JHI-Hv50k-2016-128164	2H	724913297	A	B	H	H	H	A	B	H	H	H	A	H	H	A	B	A	A	A	B	H	B	B	B	B	B	H	B	B	B	B	H	B	B
4933	JHI-Hv50k-2016-128173	2H	724914939	A	B	H	H	H	A	B	H	H	H	A	H	H	A	B	A	A	A	B	H	B	B	B	B	B	H	B	B	B	B	H	B	B
4948	JHI-Hv50k-2016-128362	2H	725104281	A	B	H	H	H	A	B	H	H	H	A	H	H	A	B	A	A	A	B	H	B	B	B	B	B	H	B	B	B	B	H	B	B
4950	JHI-Hv50k-2016-128392	2H	725118095	A	B	H	H	H	A	B	H	H	H	A	H	H	A	B	A	A	A	B	H	B	B	B	B	B	H	B	B	B	B	H	B	B
4953	JHI-Hv50k-2016-128447	2H	725230278	A	B	H	H	H	A	B	H	H	H	A	H	H	A	B	A	A	A	B	H	B	B	B	B	B	H	B	B	B	B	H	B	B
4954	JHI-Hv50k-2016-128451	2H	725230494	A	B	H	H	H	A	B	H	H	H	A	H	H	A	B	A	A	A	B	H	B	B	B	B	B	H	B	B	B	B	H	B	B
4959	JHI-Hv50k-2016-128523	2H	725520703	A	B	H	H	H	A	B	H	H	H	A	H	H	A	B	A	A	A	B	H	B	B	B	B	B	H	B	B	B	B	H	B	B
4961	JHI-Hv50k-2016-128530	2H	725521332	A	B	H	H	H	A	B	H	H	H	A	H	H	A	B	A	A	A	B	H	B	B	B	B	B	H	B	B	B	B	H	B	B
4966	JHI-Hv50k-2016-128643	2H	725654187	A	B	H	-	H	A	B	H	H	H	A	H	H	A	B	A	A	A	B	H	B	B	B	B	B	H	B	B	B	B	H	B	B
4971	JHI-Hv50k-2016-128659	2H	725655512	A	B	H	H	H	A	B	H	H	H	A	H	H	A	B	A	A	A	B	H	B	B	B	B	B	H	B	B	B	B	H	B	B
4974	JHI-Hv50k-2016-128726	2H	725775823	A	B	H	H	H	A	B	H	H	H	A	H	H	A	B	A	A	A	B	H	B	B	B	B	B	H	B	B	B	B	H	B	B
4975	JHI-Hv50k-2016-128729	2H	725776142	A	B	H	H	H	A	B	H	H	H	A	H	H	A	B	A	A	A	B	H	B	B	B	B	B	H	B	B	B	B	H	B	B
4977	JHI-Hv50k-2016-128757	2H	725794561	A	B	H	H	H	A	B	H	H	H	A	H	H	A	B	A	A	A	B	H	B	B	B	B	B	H	B	B	B	B	H	B	B
4979	JHI-Hv50k-2016-128784	2H	725814114	A	B	H	H	H	A	B	H	H	H	A	H	H	A	B	A	A	A	B	H	B	B	B	B	B	H	B	B	B	B	H	B	B
4984	JHI-Hv50k-2016-128884	2H	726003468	A	B	H	H	H	A	B	H	H	H	A	H	H	A	B	A	A	A	B	H	B	B	B	B	B	H	B	B	B	B	H	B	B
4985	JHI-Hv50k-2016-128918	2H	726011296	A	B	H	H	H	A	B	H	H	H	A	H	H	A	B	A	A	A	B	H	B	B	B	B	B	H	B	B	B	B	H	B	B
4986	JHI-Hv50k-2016-128924	2H	726012437	A	B	H	H	H	A	B	H	H	H	A	H	H	A	B	A	A	A	B	H	B	B	B	B	B	H	B	B	B	B	H	B	B
4987	JHI-Hv50k-2016-128936	2H	726013263	A	B	H	H	H	A	B	H	H	H	A	H	H	A	B	A	A	A	B	H	B	B	B	B	B	H	B	B	B	B	H	B	B
4988	JHI-Hv50k-2016-128967	2H	726021847	A	B	H	H	H	A	B	H	H	H	A	H	H	A	B	A	A	A	B	H	B	B	B	B	B	H	B	B	B	B	H	B	B
4989	JHI-Hv50k-2016-128994	2H	726023371	A	B	H	H	H	A	B	H	H	H	A	H	H	A	B	A	A	A	B	H	B	B	B	B	B	H	B	B	B	B	H	B	B
4990	JHI-Hv50k-2016-129026	2H	726026372	A	B	H	H	H	A	B	H	H	H	A	H	H	A	B	A	A	A	B	H	B	B	B	B	B	H	B	B	B	B	H	B	B
4991	JHI-Hv50k-2016-129045	2H	726082298	A	B	H	H	H	A	B	H	H	H	A	H	H	A	B	A	A	A	B	H	B	B	B	B	B	H	B	B	B	B	H	B	B
4997	JHI-Hv50k-2016-129097	2H	726243744	A	B	H	H	H	A	B	H	H	H	A	H	H	A	B	A	A	A	B	H	B	B	B	B	B	H	B	B	B	B	H	B	B
4999	JHI-Hv50k-2016-129258	2H	726865507	A	B	H	H	H	A	B	H	H	H	A	H	H	A	B	A	A	A	B	H	B	B	B	B	B	H	B	B	B	B	H	B	B
5003	JHI-Hv50k-2016-129366	2H	726904116	A	B	H	H	H	A	B	H	H	H	A	H	H	A	B	A	A	A	B	H	B	B	B	B	B	H	B	B	B	B	H	B	B
5004	JHI-Hv50k-2016-129384	2H	726970655	A	B	H	H	H	A	B	H	H	H	A	H	H	A	B	A	A	A	B	H	B	B	B	B	B	H	B	B	B	B	H	B	B
5006	JHI-Hv50k-2016-129529	2H	727209271	A	B	H	H	H	A	B	H	H	H	A	H	H	A	B	A	A	A	B	H	B	B	B	B	B	H	B	B	B	B	H	B	B
5007	JHI-Hv50k-2016-129532	2H	727209488	A	B	H	H	H	A	B	H	H	H	A	H	H	A	B	A	A	A	B	H	B	B	B	B	B	H	B	B	B	B	H	B	B
5009	JHI-Hv50k-2016-129545	2H	727210479	A	B	H	H	H	A	B	H	H	H	A	H	H	A	B	A	A	A	B	H	B	B	B	B	B	H	B	B	B	B	H	B	B
5010	JHI-Hv50k-2016-129546	2H	727210530	A	B	H	H	H	A	B	H	H	H	A	H	H	A	B	A	A	A	B	H	B	B	B	B	B	H	B	B	B	B	H	B	B
5014	JHI-Hv50k-2016-129603	2H	727267084	A	B	H	H	H	A	B	H	H	H	A	H	H	A	B	A	A	A	B	H	B	B	B	B	B	H	B	B	B	B	H	B	B
5015	JHI-Hv50k-2016-129614	2H	727317312	A	B	H	H	H	A	B	H	H	H	A	H	H	A	B	A	A	A	B	H	B	B	B	B	B	H	B	B	B	B	H	B	B
5023	JHI-Hv50k-2016-129782	2H	727485120	A	B	H	H	H	A	B	H	H	H	A	H	H	A	B	A	A	A	B	H	B	B	B	B	B	H	B	B	B	B	H	B	B

5029	JHI-Hv50k-2016-129870	2H	727578152	A	B	H	H	H	A	B	H	H	H	A	H	H	A	B	A	A	A	B	H	B	B	B	B	B	H	B	B	B	B	H	B	B
5034	JHI-Hv50k-2016-129980	2H	727650034	A	B	H	H	H	A	B	H	H	H	A	H	H	A	B	A	A	A	B	H	B	B	B	B	B	H	B	B	B	B	H	B	B
5036	JHI-Hv50k-2016-129984	2H	727650229	A	B	H	H	H	A	B	H	H	H	A	H	H	A	B	A	A	A	B	H	B	B	B	B	B	H	B	B	B	B	H	B	B
5045	JHI-Hv50k-2016-130079	2H	727963299	A	B	H	H	H	A	B	H	H	H	A	H	H	A	B	A	A	A	B	H	B	B	B	B	B	H	B	B	B	B	H	B	B
5046	JHI-Hv50k-2016-130092	2H	727963982	A	B	H	H	H	A	B	H	H	H	A	H	H	A	B	A	A	A	B	H	B	B	B	B	B	H	B	B	B	B	H	B	B
5048	JHI-Hv50k-2016-130099	2H	727964558	A	B	H	H	H	A	B	H	H	H	A	H	H	A	B	A	A	A	B	H	B	B	B	B	B	H	B	B	B	B	H	B	B
5051	JHI-Hv50k-2016-130123	2H	727978235	A	B	H	H	H	A	B	H	H	-	A	H	H	A	B	A	A	A	B	H	B	B	B	B	B	H	B	B	B	B	H	B	B
5052	JHI-Hv50k-2016-130125	2H	727978317	A	B	H	H	H	A	B	H	H	H	A	H	H	A	B	A	A	A	B	H	B	B	B	B	B	H	B	B	B	B	H	B	B
5054	JHI-Hv50k-2016-130137	2H	728032722	A	B	H	H	H	A	B	H	H	H	A	H	H	A	B	A	A	A	B	H	B	B	B	B	B	H	B	B	B	B	H	B	B
5060	JHI-Hv50k-2016-130203	2H	728158952	A	B	H	H	H	A	B	H	H	H	A	H	H	A	B	A	A	A	B	H	B	B	B	B	B	H	B	B	B	B	H	B	B
5061	JHI-Hv50k-2016-130206	2H	728159388	A	B	H	H	H	A	B	H	H	H	A	H	H	A	B	A	A	A	B	H	B	B	B	B	B	H	B	B	B	B	H	B	B
5062	JHI-Hv50k-2016-130223	2H	728240034	A	B	H	H	H	A	B	H	H	H	A	H	H	A	B	A	A	A	B	H	B	B	B	B	B	H	B	B	B	B	H	B	B
5066	JHI-Hv50k-2016-130257	2H	728298636	A	B	H	H	H	A	B	H	H	H	A	H	H	A	B	A	A	A	B	H	B	B	B	B	B	H	B	B	B	B	H	B	B
5067	JHI-Hv50k-2016-130259	2H	728298775	A	B	H	H	H	A	B	H	H	H	A	H	H	A	B	A	A	A	B	H	B	B	B	B	B	H	B	B	B	B	H	B	B
5068	JHI-Hv50k-2016-130260	2H	728298989	A	B	H	H	H	A	B	H	H	H	A	H	H	A	B	A	A	A	B	H	B	B	B	B	B	H	B	B	B	B	H	B	B
5071	JHI-Hv50k-2016-130387	2H	728830270	A	B	H	H	H	A	B	H	H	A	A	H	H	A	B	A	A	A	B	H	B	B	B	B	B	H	B	B	B	B	H	B	B
5072	JHI-Hv50k-2016-130389	2H	728830413	A	B	H	H	H	A	B	H	H	A	A	H	H	A	B	A	A	A	B	H	B	B	B	B	B	H	B	B	B	B	H	B	B
5073	JHI-Hv50k-2016-130413	2H	728831999	A	B	H	H	H	A	B	H	H	A	A	H	H	A	B	A	A	A	B	H	B	B	B	B	B	H	B	B	B	B	H	B	B
5076	JHI-Hv50k-2016-130469	2H	729140966	A	B	H	H	H	A	B	H	H	A	A	H	H	A	B	A	A	A	B	H	B	B	B	B	B	H	B	B	B	B	H	B	B
5077	JHI-Hv50k-2016-130522	2H	729159339	A	B	H	H	H	A	B	H	H	A	A	H	H	A	B	A	A	A	B	H	B	B	B	B	B	H	B	B	B	B	H	B	B
5078	JHI-Hv50k-2016-130575	2H	729210040	A	B	H	H	H	A	B	H	H	A	A	H	H	A	B	A	A	A	B	H	B	B	B	B	B	H	B	B	B	B	H	B	B
5094	JHI-Hv50k-2016-130645	2H	729224492	A	B	H	H	H	A	B	H	H	A	A	H	H	A	B	A	A	A	B	H	B	B	B	B	B	H	B	B	B	B	H	B	B
5097	JHI-Hv50k-2016-130683	2H	729229245	A	B	H	H	H	A	B	H	H	A	A	H	H	A	B	A	A	A	B	H	B	B	B	B	B	H	B	B	B	B	H	B	B
5106	JHI-Hv50k-2016-130757	2H	729294590	A	B	B	B	-	A	B	B	B	A	A	B	B	A	B	A	A	A	B	B	B	B	B	B	B	-	B	B	B	B	-	B	B
5107	JHI-Hv50k-2016-130764	2H	729295193	A	B	H	H	H	A	B	H	H	A	A	H	H	A	B	A	A	A	B	H	B	B	B	B	B	H	B	B	B	B	H	B	B
5115	JHI-Hv50k-2016-130854	2H	729542946	A	B	H	H	H	A	B	H	H	A	A	H	H	A	B	A	A	A	B	H	B	B	B	B	B	H	B	B	B	B	H	B	B
5117	JHI-Hv50k-2016-130867	2H	729558530	A	B	H	H	H	A	B	H	H	A	A	H	H	A	B	A	A	A	B	H	B	B	B	B	B	H	B	B	B	B	H	B	B
5118	JHI-Hv50k-2016-130920	2H	729727320	A	B	H	H	H	A	B	H	H	A	A	H	H	A	B	A	A	A	B	H	B	B	B	B	B	H	B	B	B	B	H	B	B
5119	JHI-Hv50k-2016-130926	2H	729750874	A	B	H	H	H	A	B	H	H	A	A	H	H	A	B	A	A	A	B	H	B	B	B	B	B	H	B	B	B	B	H	B	B
5123	JHI-Hv50k-2016-130949	2H	729841762	A	B	-	-	H	A	B	H	H	A	A	H	H	A	B	A	A	A	B	H	B	B	B	B	B	H	B	B	B	B	H	B	B
5124	JHI-Hv50k-2016-130956	2H	729842319	A	B	H	H	H	A	B	H	H	A	A	H	H	A	B	A	A	A	B	H	B	B	B	B	B	H	B	B	B	B	H	B	B
5132	JHI-Hv50k-2016-131016	2H	730029363	A	B	H	H	H	A	B	H	H	A	A	H	H	A	B	A	A	A	B	H	B	B	B	B	B	H	B	B	B	B	H	B	B
5135	JHI-Hv50k-2016-131068	2H	730852717	A	B	H	H	H	A	B	H	H	A	A	H	H	A	B	A	A	A	B	H	B	B	B	B	B	H	B	B	B	B	H	B	B
5139	JHI-Hv50k-2016-131130	2H	730980511	A	B	H	H	H	A	B	H	H	A	A	H	H	A	B	A	A	A	B	H	B	B	B	B	B	H	B	B	B	B	H	B	B
5142	JHI-Hv50k-2016-131168	2H	731034043	A	B	H	H	H	A	B	H	H	A	A	H	H	A	B	A	A	A	B	H	B	B	B	B	B	H	B	B	B	B	H	B	B
5148	JHI-Hv50k-2016-131231	2H	731142957	A	B	H	H	H	A	B	H	H	A	A	H	H	A	B	A	A	A	B	H	B	B	B	B	B	H	B	B	B	B	H	B	B
5151	JHI-Hv50k-2016-131291	2H	731204776	A	B	H	H	H	A	B	H	H	-	A	H	H	A	B	A	A	A	B	H	B	B	B	B	B	H	B	B	B	B	H	B	B
5152	JHI-Hv50k-2016-131292	2H	731204835	A	B	-	-	H	A	B	H	-	-	A	H	H	A	B	A	A	A	B	H	B	B	B	B	B	H	B	B	B	B	H	B	B
5153	JHI-Hv50k-2016-131311	2H	731229343	A	B	A	A	H	A	B	H	-	A	A	H	H	A	B	A	A	A	B	H	B	B	B	B	B	H	B	B	B	B	H	B	B
5155	JHI-Hv50k-2016-131322	2H	731231115	A	B	B	B	B	A	B	B	B	-	A	B	B	A	B	A	A	A	B	B	B	B	B	B	B	B	B	B	B	B	B	B	B
5156	JHI-Hv50k-2016-131327	2H	731272759	A	B	H	H	H	A	B	H	H	A	A	H	H	A	B	A	A	A	B	H	B	B	B	B	B	H	B	B	B	B	H	B	B
5157	JHI-Hv50k-2016-131341	2H	731273583	A	B	H	H	H	A	B	H	H	A	A	H	H	A	B	A	A	A	B	H	B	B	B	B	B	H	B	B	B	B	H	B	B
5158	JHI-Hv50k-2016-131359	2H	731304950	A	B	H	H	H	A	B	H	H	A	A	H	H	A	B	A	A	A	B	H	B	B	B	B	B	H	B	B	B	B	H	B	B
5159	JHI-Hv50k-2016-131363	2H	731305353	A	B	H	H	H	A	B	H	H	A	A	H	H	A	B	A	A	A	B	H	B	B	B	B	B	H	B	B	B	B	H	B	B
5162	JHI-Hv50k-2016-131389	2H	731689387	A	B	H	H	H	A	B	H	H	A	A	H	H	A	B	A	A	A	B	H	B	B	B	B	B	H	B	B	B	B	H	B	B
5163	JHI-Hv50k-2016-131412	2H	731698236	A	B	H	H	H	A	B	H	H	A	A	H	H	A	B	A	A	A	B	H	B	B	B	B	B	H	B	B	B	B	H	B	B
5170	JHI-Hv50k-2016-131436	2H	731902187	A	B	H	H	H	A	B	H	H	A	A	H	H	A	B	A	A	A	B	H	B	B	B	B	B	H	B	B	B	B	H	B	B
5174	JHI-Hv50k-2016-131463	2H	732343812	A	B	-	-	H	A	B	H	H	-	A	H	H	A	B	A	A	A	B	H	B	B	B	B	B	H	B	B	B	B	H	B	B
5175	JHI-Hv50k-2016-131473	2H	732423848	A	B	H	H	H	A	B	H	H	A	A	H	H	A	B	A	A	A	B	H	B	B	B	B	B	H	B	B	B	B	H	B	B
5181	JHI-Hv50k-2016-131493	2H	732543479	A	B	H	H	H	A	B	H	H	A	A	H	H	A	B	A	A	A	B	H	B	B	B	B	B	H	B	B	B	B	H	B	B
5187	JHI-Hv50k-2016-131545	2H	732583082	A	B	H	H	H	A	B	H	H	A	A	H	H	A	B	A	A	A	B	H	B	B	B	B	B	H	B	B	B	B	H	B	B
5189	JHI-Hv50k-2016-131588	2H	732618705	A	B	H	H	H	A	B	H	H	A	A	H	H	A	B	A	A	A	B	H	B	B	B	B	B	H	B	B	B	B	H	B	B
5190	JHI-Hv50k-2016-131590	2H	732618917	A	B	H	H	H	A	B	H	H	A	A	H	H	A	B	A	A	A	B	H	B	B	B	B	B	H	B	B	B	B	H	B	B
5191	JHI-Hv50k-2016-131610	2H	732633801	A	B	H	H	H	A	B	H	H	A	A	H	H	A	B	A	A	A	B	H	B	B	B	B	B	H	B	B	B	B	H	B	B
5197	JHI-Hv50k-2016-131677	2H	732695207	A	B	H	H	H	A	B	H	H	A	A	H	H	A	B	A	A	A	B	H	B	B	B	B	B	H	B	B	B	B	H	B	B
5198	JHI-Hv50k-2016-131682	2H	732697789	A	B	-	H	H	A	B	H	H	A	A	H	H	A	B	A	A	A	B	H	B	B	B	B	B	H	B	B	B	B	H	B	B
5201	JHI-Hv50k-2016-131709	2H	732702705	A	B	H	H	H	A	B	H	H	A	A	H	H	A	B	A	A	A	B	H	B	B	B	B	B	H	B	B	B	B	H	B	B
5202	JHI-Hv50k-2016-131711	2H	732703341	A	B	H	H	H	A	B	H	H	A	A	H	H	A	B	A	A	A	B	H	B	B	B	B	B	H	B	B	B	B	H	B	B
5204	JHI-Hv50k-2016-131717	2H	732704168	A	B	H	H	H	A	B	H	H	A	A	H	H	A	B	A	A	A	B	H	B	B	B	B	B	H	B	B	B	B	H	B	B
5212	JHI-Hv50k-2016-131956	2H	733155945	A	B	H	H	H	A	B	H	H	A	A	H	H	A	B	A	A	A	B	H	B	B	B	B	B	H	B	B	B	B	H	B	B
5214	JHI-Hv50k-2016-131986	2H	733393195	A	B	H	H	H	A	B	H	H	A	A	H	H	A	B	A	A	A	B	H	B	B	B	B	B	H	B	B	B	B	H	B	B
5223	JHI-Hv50k-2016-132074	2H	733499809	A	B	H	H	H	A	B	H	H	A	A	H	H	A	B	A	A	A	B	H	B	B	B	B	B	H	B	B	B	B	H	B	B
5224	JHI-Hv50k-2016-132105	2H	733502210	A	B	H	H	H	A	B	H	H	A	A	H	H	A	B	A	A	A	B	H	B	B	B	B	B	H	B	B	B	B	H	B	B
5230	JHI-Hv50k-2016-132173	2H	733526540	A	B	H	H	H	A	B	H	H	A	A	H	H	A	B	A	A	A	B	H	B	B	B	B	B	H	B	B	B	B	H	B	B
5232	JHI-Hv50k-2016-132196	2H	733666799	A	B	H	H	H	A	B	H	H	A	A	H	H	A	B	A	A	A	B	H	B	B	B	B	B	H	B	B	B	B	H	B	B
5236	JHI-Hv50k-2016-132238	2H	733684674	A	B	H	H	H	A	B	H	H	A	A	H	H	A	B	A	A	A	B	H	B	B	B	B	B	H	B	B	B	B	H	B	B
5238	JHI-Hv50k-2016-132248	2H	733685002	A	B	A	A	-	A	B	A	A	A	A	A	A	A	B	A	A	A	B	-	B	B	B	B	B	-	B	B	B	B	A	B	B
5242	JHI-Hv50k-2016-132254	2H	733685277	A	B	H	H	H	A	B	H	H	A	A	H	H	A	B	A	A	A	B	H	B	B	B	B	B	H	B	B	B	B	H	B	B
5243	JHI-Hv50k-2016-132258	2H	733685496	A	B	H	H	H	A	B	H	H	A	A	H	H	A	B	A	A	A	B	H	B	B	B	B	B	H	B	B	B	B	H	B	B
5250	JHI-Hv50k-2016-132317	2H	733688594	A	B	H	H	H	A	B	H	H	A	A	H	H	A	B	A	A	A	B	H	B	B	B	B	B	H	B	B	B	B	H	B	B
5253	JHI-Hv50k-2016-132327	2H	733688990	A	B	H	H	H	A	B	H	H	A	A	H	H	A	B	A	A	A	B	H	B	B	B	B	B	H	B	B	B	B	H	B	B
5254	JHI-Hv50k-2016-132365	2H	733691100	A	B	H	H	H	A	B	H	H	A	A	H	H	A	B	A	A	A	B	H	B	B	B	B	B	H	B	B	B	B	H	B	B
5257	JHI-Hv50k-2016-132384	2H	733695514	A	B	H	H	H	A	B	H	H	A	A	H	H	A	B	A	A	A	B	H	B	B	B	B	B	H	B	B	B	B	H	B	B
5258	JHI-Hv50k-2016-132385	2H	733695576	A	B	H	H	H	A	B	H	H	A	A	H	H	A	B	A	A	A	B	H	B	B	B	B	B	H	B	B	B	B	H	B	B
5263	JHI-Hv50k-2016-132415	2H	733917652	A	B	H	H	H	A	B	H	H	A	A	H	H	A	B	A	A	A	B	H	B	B	B	B	B	H	B	B	B	B	H	B	B
5264	JHI-Hv50k-2016-132416	2H	733917690	A	B	H	H	H	A	B	H	H	A	A	H	H	A	B	A	A	A	B	H	B	B	B	B	B	H	B	B	B	B	H	B	B
5265	JHI-Hv50k-2016-132427	2H	733924825	A	B	H	H	H	A	B	H	H	A	A	H	H	A	B	A	A	A	B	H	B	B	B	B	B	H	B	B	B	B	H	B	B
5266	JHI-Hv50k-2016-132428	2H	733925058	A	B	H	H	H	A	B	H	H	A	A	H	H	A	B	A	A	A	B	H	B	B	B	B	B	H	B	B	B	B	H	B	B
5269	JHI-Hv50k-2016-132440	2H	733926337	A	B	H	H	H	A	B	H	H	A	A	H	H	A	B	A	A	A	B	H	B	B	B	B	B	H	B	B	B	B	H	B	B
5274	JHI-Hv50k-2016-132483	2H	734126714	A	B	H	H	H	A	B	H	H	A	A	H	H	A	B	A	A	A	B	H	B	B	B	B	B	H	B	B	B	B	H	B	B
5276	JHI-Hv50k-2016-132508	2H	734178629	A	B	H	H	H	A	B	H	H	A	A	H	H	A	B	A	A	A	B	H	B	B	B	B	B	H	B	B	B	B	H	B	B
5279	JHI-Hv50k-2016-132529	2H	734189807	A	B	H	H	H	A	B	H	H	A	A	H	H	A	B	A	A	A	B	H	B	B	B	B	B	H	B	B	B	B	H	B	B
5280	JHI-Hv50k-2016-132531	2H	734189962	A	B	H	H	H	A	B	H	H	A	A	H	H	A	B	A	A	A	B	H	B	B	B	B	B	H	B	B	B	B	H	B	B
5281	JHI-Hv50k-2016-132538	2H	734194363	A	B	H	H	H	A	B	H	H	A	A	H	H	A	B	A	A	A	B	H	B	B	B	B	B	H	B	B	B	B	H	B	B
5282	JHI-Hv50k-2016-132561	2H	734335186	A	B	H	H	H	A	B	H	H	A	A	H	H	A	B	A	A	A	B	H	B	B	B	B	B	H	B	B	B	B	H	B	B
5284	JHI-Hv50k-2016-132577	2H	734431895	A	B	H	H	H	A	B	H	H	A	A	H	H	A	B	A	A	A	B	H	B	B	B	B	B	H	B	B	B	B	H	B	B
5288	JHI-Hv50k-2016-132608	2H	734457857	A	B	H	H	H	A	B	H	H	A	A	H	H	A	B	A	A	A	B	H	B	B	B	B	B	H	B	B	B	B	H	B	B
5293	JHI-Hv50k-2016-132630	2H	734460560	A	B	A	A	A	A	-	A	A	A	A	A	A	A	-	A	A	A	-	A	-	-	-	-	-	A	B	B	-	B	A	-	-
5296	JHI-Hv50k-2016-132670	2H	734501016	A	B	H	H	H	A	B	H	H	A	A	H	H	A	B	A	A	A	B	H	B	B	B	B	B	H	B	B	B	B	H	B	B
5297	JHI-Hv50k-2016-132671	2H	734501176	A	B	H	H	H	A	B	H	H	H	A	H	H	A	B	A	A	A	B	H	B	B	B	B	B	H	B	B	B	B	H	B	B
5303	JHI-Hv50k-2016-132763	2H	734858477	A	B	A	-	H	A	B	H	A	A	A	H	H	A	B	A	A	A	B	H	B	B	B	B	B	H	B	B	B	B	H	B	B
5304	JHI-Hv50k-2016-132771	2H	734859614	A	B	-	H	H	A	B	H	H	-	A	H	H	A	B	-	A	A	B	H	B	B	B	B	B	H	B	B	B	B	H	B	B
5305	JHI-Hv50k-2016-132785	2H	734871088	A	B	H	H	H	A	B	H	H	A	A	H	H	A	B	A	A	A	B	H	B	B	B	B	B	H	B	B	B	B	H	B	B
5306	JHI-Hv50k-2016-132801	2H	735080290	A	B	H	H	H	A	B	H	H	A	A	H	H	A	B	A	A	A	B	H	B	B	B	B	B	H	B	B	B	B	H	B	B
5307	JHI-Hv50k-2016-132804	2H	735080430	A	B	H	H	H	A	B	H	H	A	A	H	H	A	B	A	A	A	B	H	B	B	B	B	B	H	B	B	B	B	H	B	B
5310	JHI-Hv50k-2016-132811	2H	735081531	A	B	H	H	H	A	B	H	H	A	A	H	H	A	B	A	A	A	B	H	B	B	B	B	B	H	B	B	B	B	H	B	B
5312	JHI-Hv50k-2016-132814	2H	735082241	A	B	H	H	H	A	B	H	H	A	A	H	H	A	B	A	A	A	B	H	B	B	B	B	B	H	B	B	B	B	H	B	B
5313	JHI-Hv50k-2016-132817	2H	735082601	A	B	H	H	H	A	B	H	H	A	A	H	H	A	B	A	A	A	B	H	B	B	B	B	B	H	B	B	B	B	H	B	B
5316	JHI-Hv50k-2016-132830	2H	735083984	A	B	H	H	H	A	B	H	H	A	A	H	H	A	B	A	A	A	B	H	B	B	B	B	B	H	B	B	B	B	H	B	B
5318	JHI-Hv50k-2016-132845	2H	735115161	A	B	H	H	H	A	B	H	H	A	A	H	H	A	B	A	A	A	B	H	B	B	B	B	B	H	B	B	B	B	H	B	B
5321	JHI-Hv50k-2016-132853	2H	735119986	A	B	-	-	H	A	B	H	-	A	A	H	H	A	B	A	A	A	B	H	B	B	B	B	B	H	B	B	B	B	H	B	B
5326	JHI-Hv50k-2016-132982	2H	735299956	A	B	H	H	H	A	B	H	H	A	A	H	H	A	B	A	A	A	B	H	B	B	B	B	B	H	B	B	B	B	H	B	B
5327	JHI-Hv50k-2016-132986	2H	735300130	A	B	-	-	H	A	B	H	H	A	A	H	H	A	B	A	A	A	B	H	B	B	B	B	B	H	B	B	B	B	H	B	B
5328	JHI-Hv50k-2016-132988	2H	735300329	A	B	H	H	H	A	B	H	H	A	A	H	H	A	B	A	A	A	B	H	B	B	B	B	B	H	B	B	B	B	H	B	B
5329	JHI-Hv50k-2016-133019	2H	735302807	A	B	H	H	H	A	B	H	H	A	A	H	H	A	B	A	A	A	B	H	B	B	B	B	B	H	B	B	B	B	H	B	B
5331	JHI-Hv50k-2016-133055	2H	735380416	A	B	H	H	H	A	B	H	H	A	A	H	H	A	B	A	A	A	B	H	B	B	B	B	B	H	B	B	B	B	H	B	B
5332	JHI-Hv50k-2016-133067	2H	735389799	A	B	H	H	H	A	B	H	H	A	A	H	H	A	B	A	A	A	B	H	B	B	B	B	B	H	B	B	B	B	H	B	B

5333	JHI-Hv50k-2016-133076	2H	735390089	A	B	H	H	H	A	B	H	H	A	A	H	H	A	B	A	A	A	B	H	B	B	B	B	B	H	B	B	B	B	H	B	B
5334	JHI-Hv50k-2016-133079	2H	735390155	A	B	H	H	H	A	B	H	H	A	A	H	H	A	B	A	A	A	B	H	B	B	B	B	B	H	B	B	B	B	H	B	B
5335	JHI-Hv50k-2016-133080	2H	735390224	A	B	H	H	H	A	B	H	H	A	A	H	H	A	B	A	A	A	B	H	B	B	B	B	B	H	B	B	B	B	H	B	B
5338	JHI-Hv50k-2016-133112	2H	735404593	A	B	H	H	H	A	B	H	H	A	A	H	H	A	B	A	A	A	B	H	B	B	B	B	B	H	B	B	B	B	H	B	B
5340	JHI-Hv50k-2016-133167	2H	736212969	A	B	H	H	H	A	B	H	H	A	A	H	H	A	B	A	A	A	B	-	B	B	B	B	B	H	B	B	B	B	H	B	B
5341	JHI-Hv50k-2016-133174	2H	736213883	A	B	H	H	H	A	B	H	H	A	A	H	H	A	B	A	A	A	B	H	B	B	B	B	B	H	B	B	B	B	H	B	B
5342	JHI-Hv50k-2016-133181	2H	736214477	A	B	H	H	H	A	B	H	H	A	A	H	H	A	B	A	A	A	B	H	B	B	B	B	B	H	B	B	B	B	H	B	B
5343	JHI-Hv50k-2016-133197	2H	736236666	A	B	H	H	H	A	B	H	H	A	A	H	H	A	B	A	A	A	B	H	B	B	B	B	B	H	B	B	B	B	H	B	B
5344	JHI-Hv50k-2016-133198	2H	736236835	A	B	H	H	H	A	B	H	H	A	A	H	H	A	B	A	A	A	B	H	B	B	B	B	B	H	B	B	B	B	H	B	B
5345	JHI-Hv50k-2016-133208	2H	736245865	A	B	H	H	H	A	B	H	H	A	A	H	H	A	B	A	A	A	B	H	B	B	B	B	B	H	B	B	B	B	H	B	B
5346	JHI-Hv50k-2016-133214	2H	736246745	A	B	H	H	H	A	B	H	H	A	A	H	H	A	B	A	A	A	B	H	B	B	B	B	B	H	B	B	B	B	H	B	B
5347	JHI-Hv50k-2016-133218	2H	736246911	A	B	H	H	H	A	B	H	H	A	A	H	H	A	B	A	A	A	B	H	B	B	B	B	B	H	B	B	B	B	H	B	B
5352	JHI-Hv50k-2016-133293	2H	736369658	A	B	H	H	H	A	B	H	H	A	A	H	H	A	B	A	A	A	B	H	B	B	B	B	B	H	B	B	B	B	H	B	B
5356	JHI-Hv50k-2016-133336	2H	736751512	A	B	H	H	H	A	B	H	H	A	A	H	H	A	B	A	A	A	B	H	B	B	B	B	B	H	B	B	B	B	H	B	B
5358	JHI-Hv50k-2016-133345	2H	736753860	A	B	H	H	H	A	B	H	H	A	A	H	H	A	B	A	A	A	B	H	B	B	B	B	B	H	B	B	B	B	H	B	B
5361	JHI-Hv50k-2016-133390	2H	737026029	A	B	H	H	H	A	B	H	H	A	A	H	H	A	B	A	A	A	B	H	B	B	B	B	B	H	B	B	B	B	H	B	B
5362	JHI-Hv50k-2016-133408	2H	737028354	A	B	H	H	H	A	B	H	H	A	A	H	H	A	B	A	A	A	B	H	B	B	B	B	B	H	B	B	B	B	H	B	B
5371	JHI-Hv50k-2016-133510	2H	737177482	A	B	H	H	H	A	B	H	H	A	A	H	H	A	B	A	A	A	B	H	B	B	B	B	B	H	B	B	B	B	H	B	B
5373	JHI-Hv50k-2016-133573	2H	737639643	A	B	H	H	H	A	B	H	H	A	A	H	H	A	B	A	A	A	B	H	B	B	B	B	B	H	B	B	B	B	H	B	B
5374	JHI-Hv50k-2016-133575	2H	737639735	A	B	H	H	H	A	B	H	H	A	A	H	H	A	B	A	A	A	B	H	B	B	B	B	B	H	B	B	B	B	H	B	B
5376	JHI-Hv50k-2016-133595	2H	737641164	A	B	H	H	H	A	B	H	H	A	A	H	H	A	B	A	A	A	B	H	B	B	B	B	B	H	B	B	B	B	H	B	B
5377	JHI-Hv50k-2016-133604	2H	737641739	A	B	H	H	H	A	B	H	H	A	A	H	H	A	B	A	A	A	B	H	B	B	B	B	B	H	B	B	B	B	H	B	B
5378	JHI-Hv50k-2016-133605	2H	737641821	A	B	H	H	H	A	B	H	H	A	A	H	H	A	B	A	A	A	B	H	B	B	B	B	B	H	B	B	B	B	H	B	B
5379	JHI-Hv50k-2016-133639	2H	737644534	A	B	H	H	H	A	B	H	H	A	A	H	H	A	B	A	A	A	B	H	B	B	B	B	B	H	B	B	B	B	H	B	B
5380	JHI-Hv50k-2016-133655	2H	737645544	A	B	H	H	H	A	B	H	H	A	A	H	H	A	B	A	A	A	B	H	B	B	B	B	B	H	B	B	B	B	H	B	B
5382	JHI-Hv50k-2016-133699	2H	737655839	A	B	H	H	H	A	B	H	H	A	A	H	H	A	B	A	A	A	B	H	B	B	B	B	B	H	B	B	B	B	H	B	B
5384	JHI-Hv50k-2016-133720	2H	737660910	A	B	H	H	H	A	B	H	H	A	A	H	H	A	B	A	A	A	B	H	B	B	B	B	B	H	B	B	B	B	H	B	B
5389	JHI-Hv50k-2016-133808	2H	738054672	A	B	B	B	H	A	B	H	-	H	A	H	H	A	B	-	A	A	B	H	B	B	B	B	B	H	B	B	B	B	H	B	B
5390	JHI-Hv50k-2016-133812	2H	738054984	A	B	H	H	H	A	B	H	H	A	A	H	H	A	B	A	A	A	B	H	B	B	B	B	B	H	B	B	B	B	H	B	B
5394	JHI-Hv50k-2016-133841	2H	738326718	A	B	H	H	H	A	B	H	H	A	A	H	H	A	B	A	A	A	B	H	B	B	B	B	B	H	B	B	B	B	H	B	B
5395	JHI-Hv50k-2016-133850	2H	738342065	A	B	H	H	H	A	B	H	H	A	A	H	H	A	B	A	A	A	B	H	B	B	B	B	B	H	B	B	B	B	H	B	B
5402	JHI-Hv50k-2016-133904	2H	738614758	A	B	H	-	H	A	B	H	H	A	A	H	H	A	B	A	A	A	B	H	B	B	B	B	B	H	B	B	B	B	H	B	B
5421	JHI-Hv50k-2016-134196	2H	739244211	A	B	H	H	H	A	B	H	H	A	A	H	H	A	B	A	A	A	B	H	B	B	B	B	B	H	B	B	B	B	H	B	B
5437	JHI-Hv50k-2016-134401	2H	739584855	A	B	H	H	H	A	B	H	H	A	A	H	H	A	B	A	A	A	B	H	B	B	B	B	B	H	B	B	B	B	H	B	B
5438	JHI-Hv50k-2016-134432	2H	739588385	A	B	H	H	H	A	B	H	H	A	A	H	H	A	B	A	A	A	B	H	B	B	B	B	B	H	B	B	B	B	H	B	B
5440	JHI-Hv50k-2016-134486	2H	739994452	A	B	H	H	H	A	B	H	H	A	A	H	H	A	B	A	A	A	B	H	B	B	B	B	B	B	B	B	B	B	H	B	B
5441	JHI-Hv50k-2016-134491	2H	739994904	A	B	H	H	H	A	B	H	H	A	A	H	H	A	B	A	A	A	B	H	B	B	B	B	B	B	B	B	B	B	H	B	B
5442	JHI-Hv50k-2016-134503	2H	739996053	A	B	H	H	H	A	B	H	H	A	A	H	H	A	B	A	A	A	B	H	B	B	B	B	B	B	B	B	B	B	H	B	B
5444	JHI-Hv50k-2016-134583	2H	740038414	A	B	H	H	H	A	B	H	H	A	A	H	H	A	B	A	A	A	B	H	B	B	B	B	B	B	B	B	B	B	-	B	B
5446	JHI-Hv50k-2016-134740	2H	740255609	A	B	H	H	H	A	B	H	H	A	A	H	H	A	B	A	A	A	B	H	B	B	B	B	B	B	B	B	B	B	H	B	B
5447	JHI-Hv50k-2016-134751	2H	740256672	A	B	H	H	H	A	B	H	H	A	A	H	H	A	B	A	A	A	B	H	B	B	B	B	B	B	B	B	B	B	H	B	B
5448	JHI-Hv50k-2016-134802	2H	740414038	A	B	H	H	H	A	B	H	H	A	A	H	H	A	B	A	A	A	B	H	B	B	B	B	B	B	B	B	B	B	H	B	B
5449	JHI-Hv50k-2016-134808	2H	740414463	A	B	H	H	H	A	B	H	H	A	A	H	H	A	B	A	A	A	B	H	B	B	B	B	B	B	B	B	B	B	H	B	B
5451	JHI-Hv50k-2016-134862	2H	740580427	A	B	H	-	H	-	B	H	H	H	A	H	H	A	B	A	-	A	B	H	B	B	B	B	B	B	B	B	B	B	H	B	B
5453	JHI-Hv50k-2016-134872	2H	740689110	A	B	H	H	H	A	B	H	H	A	A	H	H	A	B	A	A	A	B	H	B	B	B	B	B	B	B	B	B	B	H	B	B
5455	JHI-Hv50k-2016-134874	2H	740691605	A	B	H	H	H	A	B	H	H	A	A	H	H	A	B	A	A	A	B	H	B	B	B	B	B	B	B	B	B	B	H	B	B
5459	JHI-Hv50k-2016-134947	2H	740892231	A	B	H	-	H	A	B	H	H	A	A	H	H	A	B	A	A	A	B	H	B	B	B	B	B	B	B	B	B	B	H	B	B
5469	JHI-Hv50k-2016-135041	2H	740990945	A	B	H	H	H	H	B	H	H	A	A	H	H	A	B	A	A	A	B	H	B	B	B	B	B	B	B	B	B	B	H	B	B
5472	JHI-Hv50k-2016-135056	2H	741019299	A	B	H	H	H	A	B	H	H	A	A	H	H	A	B	A	A	A	B	H	B	B	B	B	B	B	B	B	B	B	H	B	B
5473	JHI-Hv50k-2016-135074	2H	741027234	A	B	H	H	H	A	B	H	H	A	A	H	H	A	B	A	A	A	B	H	B	B	B	B	B	B	B	B	B	B	H	B	B
5477	JHI-Hv50k-2016-135102	2H	741037594	A	B	H	H	H	A	B	H	H	A	A	H	H	A	B	A	A	A	B	H	B	B	B	B	B	B	B	B	B	B	H	B	B
5479	JHI-Hv50k-2016-135143	2H	741100706	A	B	H	H	H	H	B	H	H	A	A	H	H	A	B	A	A	A	B	H	B	B	B	B	B	B	B	B	B	B	H	B	B
5486	JHI-Hv50k-2016-135227	2H	741282855	A	B	-	-	H	H	B	H	H	A	A	H	H	A	B	A	A	A	B	H	B	B	B	B	B	B	B	B	B	B	H	B	B
5487	JHI-Hv50k-2016-135232	2H	741283325	A	B	H	H	H	H	B	H	H	A	A	H	H	A	B	A	A	A	B	H	B	B	B	B	B	B	B	B	B	B	H	B	B
5488	JHI-Hv50k-2016-135316	2H	741603188	A	B	H	H	H	H	B	H	H	A	A	H	H	A	B	A	A	A	B	H	B	B	B	B	B	B	B	B	B	B	H	B	B
5489	JHI-Hv50k-2016-135356	2H	741621918	A	B	H	H	H	H	B	H	H	A	A	H	H	A	B	A	A	A	B	H	B	B	B	B	B	B	B	B	B	B	H	B	B
5491	JHI-Hv50k-2016-135382	2H	741642582	A	B	H	H	H	H	B	H	H	A	A	H	H	A	B	A	A	A	B	H	B	B	B	B	B	B	B	B	B	B	H	B	B
5494	JHI-Hv50k-2016-135426	2H	741978074	A	B	H	H	H	H	B	H	H	A	A	H	H	A	B	A	A	A	B	H	B	B	B	B	B	B	B	B	B	B	H	B	B
5498	JHI-Hv50k-2016-135435	2H	741980209	A	B	-	-	H	H	B	H	H	A	A	H	H	A	B	A	A	A	B	H	B	B	B	B	B	B	B	B	B	B	H	B	B
5499	JHI-Hv50k-2016-135438	2H	741986594	A	B	H	H	H	H	B	H	H	A	A	H	H	A	B	A	A	A	B	H	B	B	B	B	B	B	B	B	B	B	H	B	B
5502	JHI-Hv50k-2016-135470	2H	742003077	A	B	H	H	H	H	B	H	H	-	A	H	H	A	B	A	A	A	B	H	B	B	B	B	B	B	B	B	B	B	H	B	B
5503	JHI-Hv50k-2016-135474	2H	742003408	A	B	H	H	H	H	B	H	H	A	A	H	H	A	B	A	A	A	B	H	B	B	B	B	B	B	B	B	B	B	H	B	B
5504	JHI-Hv50k-2016-135537	2H	742046586	A	B	H	H	H	H	B	H	H	A	A	H	H	A	B	A	A	A	B	H	B	B	B	B	B	B	B	B	B	B	H	B	B
5511	JHI-Hv50k-2016-135623	2H	742094445	A	B	H	H	H	H	B	H	H	A	A	H	H	A	B	A	A	A	B	H	B	B	B	B	B	B	B	B	B	B	H	B	B
5519	JHI-Hv50k-2016-135641	2H	742104436	A	B	H	H	H	H	B	H	H	A	A	H	H	A	B	A	A	A	B	H	B	B	B	B	B	B	B	B	B	B	H	B	B
5540	JHI-Hv50k-2016-135937	2H	742285724	A	B	H	H	H	H	B	H	H	A	A	H	H	A	B	A	A	A	B	H	B	B	B	B	B	B	B	B	B	B	H	B	B
5544	JHI-Hv50k-2016-136049	2H	742648602	A	B	H	H	H	H	B	H	H	A	A	H	H	A	B	A	A	A	B	H	B	B	B	B	B	B	B	B	B	B	H	B	B
5545	JHI-Hv50k-2016-136058	2H	742649194	A	B	H	H	H	H	B	H	H	A	A	H	H	A	B	A	A	A	B	H	B	B	B	B	B	B	B	B	B	B	H	B	B
5546	JHI-Hv50k-2016-136071	2H	742794935	A	B	H	H	H	H	B	H	H	A	A	H	H	A	B	A	A	A	B	H	B	B	B	B	B	B	B	B	B	B	H	B	B
5547	JHI-Hv50k-2016-136072	2H	742794992	A	B	H	H	H	H	B	H	H	A	A	H	H	A	B	A	A	A	B	H	B	B	B	B	B	B	B	B	B	B	H	B	B
5552	JHI-Hv50k-2016-136125	2H	742851643	A	B	H	H	H	H	B	H	H	A	A	H	H	A	B	A	A	A	B	H	B	B	B	B	B	B	B	B	B	B	H	B	B
5555	JHI-Hv50k-2016-136149	2H	743019373	A	B	H	H	H	H	B	H	H	A	A	H	H	A	B	A	A	A	B	H	B	B	B	B	B	B	B	B	B	B	H	B	B
5556	JHI-Hv50k-2016-136164	2H	743020701	A	B	H	H	H	H	B	H	H	A	A	H	H	A	B	A	A	A	B	H	B	B	B	B	B	B	B	B	B	B	H	B	B
5559	JHI-Hv50k-2016-136190	2H	743139682	A	B	H	H	H	H	B	H	H	A	A	H	H	A	B	A	A	A	B	H	B	B	B	B	B	B	B	B	B	B	H	B	B
5560	JHI-Hv50k-2016-136204	2H	743140689	A	B	H	H	H	H	B	H	H	H	A	H	H	A	B	A	A	A	B	H	B	B	B	B	B	B	B	B	B	B	H	B	B
5563	JHI-Hv50k-2016-136221	2H	743143121	A	B	H	H	H	H	B	H	H	A	A	H	H	A	B	A	A	A	B	H	B	B	B	B	B	B	B	B	B	B	H	B	B
5565	JHI-Hv50k-2016-136245	2H	743172102	A	B	H	H	H	H	B	H	H	A	A	H	H	A	B	A	A	A	B	H	B	B	B	B	B	B	B	B	B	B	H	B	B
5567	JHI-Hv50k-2016-136275	2H	743174065	A	B	H	H	H	H	B	H	H	A	A	H	H	A	B	A	A	A	B	H	B	B	B	B	B	B	B	B	B	B	H	B	B
5570	JHI-Hv50k-2016-136304	2H	743181770	A	B	H	H	H	H	B	H	H	A	A	H	H	A	B	A	A	A	B	H	B	B	B	B	B	B	B	B	B	B	H	B	B
5582	JHI-Hv50k-2016-136561	2H	743378292	A	B	H	H	H	H	B	H	H	A	A	H	H	A	B	A	A	A	B	H	B	B	B	B	B	B	B	B	B	B	H	B	B
5584	JHI-Hv50k-2016-136583	2H	743380042	A	B	H	H	H	H	B	H	H	A	A	H	H	A	B	A	A	A	B	H	B	B	B	B	B	B	B	B	B	B	H	B	B
5585	JHI-Hv50k-2016-136587	2H	743380297	A	B	H	H	H	H	B	H	H	A	A	H	H	A	B	A	A	A	B	H	B	B	B	B	B	B	B	B	B	B	H	B	B
5586	JHI-Hv50k-2016-136633	2H	743455320	A	B	H	H	H	H	B	H	H	A	A	H	H	A	B	A	A	A	B	H	B	B	B	B	B	B	B	B	B	B	H	B	B
5588	JHI-Hv50k-2016-136635	2H	743455551	A	B	H	H	H	H	B	H	H	A	A	H	H	A	B	A	A	A	B	H	B	B	B	B	B	B	B	B	B	B	H	B	B
5592	JHI-Hv50k-2016-136728	2H	743758316	A	B	H	H	H	H	B	H	H	A	A	H	H	A	B	A	A	A	B	H	B	B	B	B	B	B	B	B	B	B	H	B	B
5593	JHI-Hv50k-2016-136731	2H	743759059	A	B	H	H	H	H	B	H	H	A	A	H	H	A	B	A	A	A	B	H	B	B	B	B	B	B	B	B	B	B	H	B	B
5594	JHI-Hv50k-2016-136732	2H	743759302	A	B	H	H	H	H	B	H	H	A	A	H	H	A	B	A	A	A	B	H	B	B	B	B	B	B	B	B	B	B	H	B	B
5596	JHI-Hv50k-2016-136859	2H	744438837	A	B	H	H	H	H	B	H	H	A	A	H	H	A	B	A	A	A	B	H	B	B	B	B	B	B	B	B	B	B	H	B	B
5599	JHI-Hv50k-2016-136929	2H	744576938	A	B	H	H	H	H	B	H	H	A	A	H	H	A	B	A	A	A	B	H	B	B	B	B	B	B	B	B	B	B	H	B	B
5602	JHI-Hv50k-2016-136937	2H	744583482	A	B	H	H	H	H	B	H	H	A	A	H	H	A	B	A	A	A	B	H	B	B	B	B	B	B	B	B	B	B	H	B	B
5604	JHI-Hv50k-2016-136962	2H	744662284	A	B	-	H	H	H	B	H	H	A	A	H	H	A	B	A	A	A	B	H	B	B	B	B	B	B	B	B	B	B	H	B	B
5606	JHI-Hv50k-2016-136999	2H	744765171	A	B	H	H	H	H	B	H	H	A	A	H	H	A	B	A	A	A	B	H	B	B	B	B	B	B	B	B	B	B	H	B	B
5608	JHI-Hv50k-2016-137000	2H	744765246	A	B	H	-	H	H	B	H	H	A	A	H	H	A	B	A	A	A	B	H	B	B	B	B	B	B	B	B	B	B	H	B	B
5609	JHI-Hv50k-2016-137012	2H	744774301	A	B	H	H	H	H	B	H	H	A	A	H	H	A	B	A	A	A	B	H	B	B	B	B	B	B	B	B	B	B	H	B	B
5610	JHI-Hv50k-2016-137020	2H	744775750	A	B	H	H	H	H	B	H	H	A	A	H	H	A	B	A	A	A	B	H	B	B	B	B	B	B	B	B	B	B	H	B	B
5611	JHI-Hv50k-2016-137060	2H	744825209	A	B	-	-	H	H	B	H	H	A	A	H	H	A	B	A	A	A	B	H	B	B	B	B	B	B	B	B	B	B	H	B	B
5612	JHI-Hv50k-2016-137061	2H	744825277	A	B	H	H	H	H	B	H	H	A	A	H	H	A	B	A	A	A	B	H	B	B	B	B	B	B	B	B	B	B	H	B	B
5613	JHI-Hv50k-2016-137071	2H	744833078	A	B	H	H	H	H	B	H	H	A	A	H	H	A	B	A	A	A	B	H	B	B	B	B	B	B	B	B	B	B	H	B	B
5622	JHI-Hv50k-2016-137127	2H	744899470	A	B	H	H	H	H	B	H	H	A	A	H	H	A	B	A	A	A	B	H	B	B	B	B	B	B	B	B	B	B	H	B	B
5623	JHI-Hv50k-2016-137128	2H	744899559	A	B	H	H	H	H	B	H	H	A	A	H	H	A	B	A	A	A	B	H	B	B	B	B	B	B	B	B	B	B	H	B	B
5624	JHI-Hv50k-2016-137159	2H	744900332	A	B	A	A	A	A	B	-	A	A	A	A	A	A	B	A	A	A	B	-	B	B	B	B	B	B	B	B	B	B	-	B	B
5628	JHI-Hv50k-2016-137311	2H	745069584	A	B	H	H	H	H	B	H	H	A	A	H	H	A	B	A	A	A	B	H	B	B	B	B	B	B	B	B	B	B	H	B	B
5630	JHI-Hv50k-2016-137315	2H	745070219	A	B	H	H	H	H	B	H	H	A	A	H	H	A	B	A	A	A	B	H	B	B	B	B	B	B	B	B	B	B	H	B	B
5634	JHI-Hv50k-2016-137359	2H	745303112	A	B	H	H	H	H	B	H	H	A	A	H	H	A	B	A	A	A	B	H	B	B	B	B	B	B	B	B	B	B	H	B	B
5635	JHI-Hv50k-2016-137369	2H	745320358	A	B	H	H	H	H	B	H	H	A	A	H	H	A	B	A	A	A	B	H	B	B	B	B	B	B	B	B	B	B	H	B	B
5647	JHI-Hv50k-2016-137415	2H	745332747	A	B	H	H	H	H	B	H	H	A	A	H	H	A	B	A	A	A	B	H	B	B	B	B	B	B	B	B	B	B	H	B	B
5648	JHI-Hv50k-2016-137417	2H	745333037	A	B	H	H	H	H	B	H	H	A	A	H	H	A	B	A	A	A	B	H	B	B	B	B	B	B	B	B	B	B	H	B	B

5649	JHI-Hv50k-2016-137428	2H	745334283	A	B	H	H	H	H	B	H	H	A	A	H	H	A	B	A	A	A	B	H	B	B	B	B	B	B	B	B	B	B	H	B	B
5651	JHI-Hv50k-2016-137432	2H	745334813	A	B	H	H	H	H	B	H	H	A	A	H	H	A	B	A	A	A	B	H	B	B	B	B	B	B	B	B	B	B	H	B	B
5652	JHI-Hv50k-2016-137433	2H	745334890	A	B	H	H	H	H	B	H	H	A	A	H	H	A	B	A	A	A	B	H	B	B	B	B	B	B	B	B	B	B	H	B	B
5661	JHI-Hv50k-2016-137586	2H	746329951	A	B	H	H	H	H	H	H	H	A	A	H	H	A	B	A	A	A	B	H	B	B	B	B	B	B	B	B	B	B	B	B	B
5665	JHI-Hv50k-2016-137652	2H	746372802	A	B	H	H	H	H	H	H	H	A	A	H	H	A	B	A	A	A	B	H	B	B	B	B	B	B	B	B	B	B	B	B	B
5676	JHI-Hv50k-2016-137786	2H	746569292	A	B	H	H	H	H	H	H	H	A	A	H	H	A	B	A	A	A	B	H	B	B	B	B	B	B	B	B	B	B	B	B	B
5680	JHI-Hv50k-2016-137800	2H	746572613	A	B	H	H	H	H	H	H	H	A	A	H	H	A	B	A	A	A	B	H	B	B	B	B	B	B	B	B	B	B	B	B	B
5681	JHI-Hv50k-2016-137803	2H	746573793	A	B	H	H	H	H	H	H	H	A	A	H	H	A	B	A	A	A	B	H	B	B	B	B	B	B	B	B	B	B	B	B	B
5684	JHI-Hv50k-2016-137842	2H	746744262	A	B	H	H	H	H	H	H	H	A	A	H	H	A	B	A	A	A	B	H	B	B	B	B	B	B	B	B	B	B	B	B	B
5685	JHI-Hv50k-2016-137852	2H	746744734	A	B	H	H	H	H	H	H	H	A	A	H	H	A	B	A	A	A	B	H	B	B	B	B	B	B	B	B	B	B	B	B	B
5689	JHI-Hv50k-2016-137909	2H	746990918	A	B	H	H	H	H	H	H	H	A	A	H	H	A	B	A	A	A	B	H	B	B	B	B	B	B	B	B	B	B	B	B	B
5690	JHI-Hv50k-2016-137912	2H	746991462	A	B	-	-	H	H	H	H	-	-	A	H	H	A	B	A	A	A	B	H	B	B	B	B	B	B	B	B	B	B	B	B	B
5694	JHI-Hv50k-2016-137991	2H	747125213	A	B	H	H	H	H	H	H	H	A	A	H	H	A	B	A	A	A	B	H	B	B	B	B	B	B	B	B	B	B	B	B	B
5695	JHI-Hv50k-2016-137999	2H	747127980	A	B	H	H	-	H	H	H	H	A	A	H	H	A	B	A	A	A	B	H	B	B	B	B	B	B	B	B	B	B	B	B	B
5701	JHI-Hv50k-2016-138112	2H	747409326	A	B	H	H	H	H	H	H	H	A	A	H	H	A	B	A	A	A	B	H	B	B	B	B	B	B	B	B	B	B	B	B	B
5741	JHI-Hv50k-2016-138648	2H	749598608	A	B	H	H	H	H	H	H	H	A	A	H	H	A	B	A	A	A	B	B	B	B	B	B	B	B	B	B	B	B	B	B	B
5742	JHI-Hv50k-2016-138652	2H	749598859	A	B	H	H	H	H	H	H	H	A	A	H	H	A	B	A	A	A	B	B	B	B	B	B	B	B	B	B	B	B	B	B	B
5744	JHI-Hv50k-2016-138670	2H	749599947	A	B	H	H	H	H	H	H	H	A	A	H	H	A	B	A	A	A	B	B	B	B	B	B	B	B	B	B	B	B	B	B	B
5748	JHI-Hv50k-2016-138719	2H	749741637	A	B	H	H	H	H	H	H	H	A	A	H	H	A	B	A	A	A	B	B	B	B	B	B	B	B	B	B	B	B	B	B	B
5749	JHI-Hv50k-2016-138729	2H	749741888	A	B	H	H	H	H	H	H	H	A	A	H	H	A	B	A	A	A	B	B	B	B	B	B	B	B	B	B	B	B	B	B	B
5751	JHI-Hv50k-2016-138751	2H	749753372	A	B	H	H	H	H	H	H	H	A	A	H	H	A	B	A	A	A	B	B	B	B	B	B	B	B	B	B	B	B	B	B	B
5752	JHI-Hv50k-2016-138752	2H	749753425	A	B	H	H	H	H	H	H	H	A	A	H	H	A	B	A	A	A	B	B	B	B	B	B	B	B	B	B	B	B	B	B	B
5753	JHI-Hv50k-2016-138766	2H	749753979	A	B	H	H	H	H	H	H	H	A	A	H	H	A	B	A	A	A	B	B	B	B	B	B	B	B	B	B	B	B	B	B	B
5754	JHI-Hv50k-2016-138839	2H	749813599	A	B	H	H	H	H	H	H	H	A	A	H	H	A	B	A	A	A	B	B	B	B	B	B	B	B	B	B	B	B	B	B	B
5755	JHI-Hv50k-2016-138849	2H	749857675	A	B	H	H	H	H	H	H	H	A	A	H	H	A	B	A	A	A	B	B	B	B	B	B	B	B	B	B	B	B	B	B	B
5759	JHI-Hv50k-2016-138894	2H	749995334	A	B	H	H	H	H	H	H	H	A	A	H	H	A	B	A	A	A	B	B	B	B	B	B	B	B	B	B	B	B	B	B	B
5760	JHI-Hv50k-2016-138899	2H	749995610	A	B	H	H	H	H	H	H	H	A	A	H	H	A	B	A	A	A	B	B	B	B	B	B	B	B	B	B	B	B	B	B	B
5761	JHI-Hv50k-2016-138932	2H	750041713	A	B	H	H	H	H	H	H	H	A	A	H	H	A	B	A	A	A	B	B	B	B	B	B	B	B	B	B	B	B	B	B	B
5762	JHI-Hv50k-2016-138947	2H	750042738	A	B	H	H	H	H	H	H	H	A	A	H	H	A	B	A	A	A	B	B	B	B	B	B	B	B	B	B	B	B	B	B	B
5763	JHI-Hv50k-2016-138980	2H	750114129	A	B	H	H	H	H	H	H	H	A	A	H	H	A	B	A	A	A	B	B	B	B	B	B	B	B	B	B	B	B	B	B	B
5764	JHI-Hv50k-2016-138994	2H	750144300	A	B	H	-	H	H	H	H	H	A	A	H	H	A	B	A	A	A	B	B	B	B	B	B	B	B	B	B	B	B	B	B	B
5765	JHI-Hv50k-2016-139028	2H	750301351	A	B	H	H	H	H	H	H	H	A	A	H	H	A	B	A	A	A	B	B	B	B	B	B	B	B	B	B	B	B	B	B	B
5766	JHI-Hv50k-2016-139076	2H	750406836	A	B	H	H	H	H	H	H	H	A	A	H	H	A	B	A	A	A	B	B	B	B	B	B	B	B	B	B	B	B	B	B	B
5767	JHI-Hv50k-2016-139077	2H	750406854	A	B	H	H	H	H	H	H	H	A	A	H	H	A	B	A	A	A	B	B	B	B	B	B	B	B	B	B	B	B	B	B	B
5769	JHI-Hv50k-2016-139104	2H	750465479	A	B	H	H	H	H	H	H	H	A	A	H	H	A	B	A	A	A	B	B	B	B	B	B	B	B	B	B	B	B	B	B	B
5770	JHI-Hv50k-2016-139146	2H	750523813	A	B	H	H	H	H	H	H	H	A	A	H	H	A	B	A	A	A	B	B	B	B	B	B	B	B	B	B	B	B	B	B	B
5771	JHI-Hv50k-2016-139148	2H	750524420	A	B	H	H	H	H	H	H	H	A	A	H	H	A	B	A	A	A	B	B	B	B	B	B	B	B	B	B	B	B	B	B	B
5772	JHI-Hv50k-2016-139151	2H	750524851	A	B	H	H	H	H	H	H	H	A	A	H	H	A	B	A	A	A	B	B	B	B	B	B	B	B	B	B	B	B	B	B	B
5773	JHI-Hv50k-2016-139165	2H	750531142	A	B	H	H	H	H	H	H	H	A	A	H	H	A	B	A	A	A	B	B	B	B	B	B	B	B	B	B	B	B	B	B	B
5775	JHI-Hv50k-2016-139171	2H	750612023	A	B	H	H	H	H	H	H	H	A	A	H	H	A	B	A	A	A	B	B	B	B	B	B	B	B	B	B	B	B	B	B	B
5778	JHI-Hv50k-2016-139216	2H	750620553	A	B	H	H	H	H	H	H	H	A	A	H	H	A	B	A	A	A	B	B	B	B	B	B	B	B	B	B	B	B	B	B	B
5779	JHI-Hv50k-2016-139244	2H	750627530	A	B	H	H	H	H	H	H	H	A	A	H	H	A	B	A	A	A	B	B	B	B	B	B	B	B	B	B	B	B	B	B	B
5781	JHI-Hv50k-2016-139283	2H	750747454	A	B	H	H	H	H	H	H	H	A	A	H	H	A	B	A	A	A	B	B	B	B	B	B	B	B	B	B	B	B	B	B	B
5782	JHI-Hv50k-2016-139304	2H	750764284	A	B	H	H	H	H	H	H	H	A	A	H	H	A	B	A	A	A	B	B	B	B	B	B	B	B	B	B	B	B	B	B	B
5785	JHI-Hv50k-2016-139333	2H	750812309	A	B	H	H	H	H	H	H	H	A	A	H	H	A	B	A	A	A	B	B	B	B	B	B	B	B	B	B	B	B	B	B	B
5788	JHI-Hv50k-2016-139338	2H	750812668	A	B	H	H	H	H	H	H	H	A	A	H	H	A	B	A	A	A	B	B	B	B	B	B	B	B	B	B	B	B	B	B	B
5789	JHI-Hv50k-2016-139339	2H	750812844	A	B	H	H	H	H	H	H	H	A	A	H	H	A	B	A	A	A	B	B	B	B	B	B	B	B	B	B	B	B	B	B	B
5791	JHI-Hv50k-2016-139341	2H	750813149	A	B	H	H	H	H	H	H	H	A	A	H	H	A	B	A	A	A	B	B	B	B	B	B	B	B	B	B	B	B	B	B	B
5793	JHI-Hv50k-2016-139377	2H	751013256	A	B	H	H	H	H	H	H	H	A	A	H	H	A	B	A	A	A	B	B	B	B	B	B	B	B	B	B	B	B	B	B	B
5795	JHI-Hv50k-2016-139420	2H	751017017	A	B	H	H	H	H	H	H	H	A	A	H	H	A	B	A	A	A	B	B	B	B	B	B	B	B	B	B	B	B	B	B	B
5796	JHI-Hv50k-2016-139434	2H	751128707	A	B	H	H	H	H	H	H	H	A	A	H	H	A	B	A	A	A	B	B	B	B	B	B	B	B	B	B	B	B	B	B	B
5797	JHI-Hv50k-2016-139435	2H	751128907	A	B	H	H	H	H	H	H	H	A	A	H	H	A	B	A	A	A	B	B	B	B	B	B	B	B	B	B	B	B	B	B	B
5799	JHI-Hv50k-2016-139507	2H	751205690	A	B	H	H	H	H	H	H	H	A	A	H	H	A	B	A	A	A	B	B	B	B	B	B	B	B	B	B	B	B	B	B	B
5800	JHI-Hv50k-2016-139525	2H	751206586	A	B	H	H	H	H	H	H	H	A	A	H	H	A	B	A	A	A	B	B	B	B	B	B	B	B	B	B	B	B	B	B	B
5802	JHI-Hv50k-2016-139558	2H	751301742	A	B	H	H	H	H	H	H	H	A	A	H	H	A	B	A	A	A	B	B	B	B	B	B	B	B	B	B	B	B	B	B	B
5804	JHI-Hv50k-2016-139629	2H	751554853	A	B	H	-	H	H	H	H	H	A	A	H	H	A	H	A	A	A	B	B	B	B	B	B	B	B	B	B	B	B	B	B	B
5812	JHI-Hv50k-2016-139823	2H	751893923	A	B	H	H	H	H	H	H	H	A	A	H	H	A	H	A	A	A	B	B	B	B	B	B	B	B	B	B	B	B	B	B	B
5813	JHI-Hv50k-2016-139825	2H	751894041	A	B	H	H	H	H	H	H	H	A	A	H	H	A	H	A	A	A	B	B	B	B	B	B	B	B	B	B	B	B	B	B	B
5824	JHI-Hv50k-2016-140235	2H	752071997	A	B	H	H	H	H	H	H	H	A	A	H	H	A	H	A	A	A	B	B	B	B	B	B	B	B	B	B	B	B	B	B	B
5825	JHI-Hv50k-2016-140239	2H	752072158	A	B	H	H	H	H	H	H	H	A	A	H	H	A	H	A	A	A	B	B	B	B	B	B	B	B	B	B	B	B	B	B	B
5826	JHI-Hv50k-2016-140382	2H	752361367	A	B	H	H	H	H	H	H	H	A	A	H	H	A	H	A	A	A	B	B	B	B	B	B	B	B	B	B	B	B	B	B	B
5829	JHI-Hv50k-2016-140483	2H	752928157	A	B	H	H	H	H	H	H	H	A	A	H	H	A	H	A	A	A	B	B	B	B	B	B	B	B	B	B	B	B	B	B	B
5831	JHI-Hv50k-2016-140496	2H	752961139	A	B	H	H	H	H	H	H	H	A	A	H	H	A	H	A	A	A	B	B	B	B	B	B	B	B	B	B	B	B	B	B	B
5837	JHI-Hv50k-2016-140579	2H	753099360	A	B	H	H	H	H	H	H	H	A	A	H	H	A	H	A	A	A	B	B	B	B	B	B	B	B	B	B	B	B	B	B	B
5838	JHI-Hv50k-2016-140582	2H	753099429	A	B	H	H	H	H	H	H	H	A	A	H	H	A	H	A	A	A	B	B	B	B	B	B	B	B	B	B	B	B	B	B	B
5840	JHI-Hv50k-2016-140672	2H	753122011	A	B	H	H	H	H	H	H	H	A	A	H	H	A	H	A	A	A	B	B	B	B	B	B	B	B	B	B	B	B	B	B	B
5844	JHI-Hv50k-2016-140683	2H	753123999	A	B	H	H	H	H	H	H	H	A	A	H	H	A	H	A	A	A	B	B	B	B	B	B	B	B	B	B	B	B	B	B	B
5849	JHI-Hv50k-2016-140869	2H	753303143	A	B	H	H	H	H	H	H	H	A	A	H	H	A	H	A	A	A	B	B	B	B	B	B	B	B	B	B	B	B	B	B	B
5864	JHI-Hv50k-2016-141240	2H	753924757	A	B	H	H	H	H	H	H	H	A	A	H	H	A	H	A	A	A	B	B	B	B	B	B	B	B	B	B	B	B	B	B	B
5866	JHI-Hv50k-2016-141264	2H	753926590	A	B	H	H	H	H	H	H	H	A	A	H	H	A	H	A	A	A	B	B	B	B	B	B	B	B	B	B	B	B	B	B	B
5868	JHI-Hv50k-2016-141337	2H	754498790	A	B	H	H	H	H	H	H	H	A	A	H	H	A	H	A	A	A	B	B	B	B	B	B	B	B	B	B	B	B	B	B	B
5869	JHI-Hv50k-2016-141347	2H	754502868	A	B	H	H	H	H	H	H	H	A	A	H	H	A	H	A	A	A	B	B	B	B	B	B	B	B	B	B	B	B	B	B	B
5875	JHI-Hv50k-2016-141407	2H	754561425	A	B	H	H	H	H	H	H	H	H	A	H	H	A	H	A	A	A	B	B	B	B	B	B	B	B	B	B	B	B	B	B	B
5876	JHI-Hv50k-2016-141423	2H	754603593	A	B	-	H	H	H	H	H	-	H	A	H	H	A	H	A	A	A	B	B	B	B	B	B	B	B	B	B	B	B	B	B	B
5877	JHI-Hv50k-2016-141424	2H	754603644	A	B	H	H	H	H	H	H	H	A	A	H	H	A	H	A	A	A	B	B	B	B	B	B	B	B	B	B	B	B	B	B	B
5878	JHI-Hv50k-2016-141429	2H	754603743	A	B	H	H	H	H	H	H	H	A	A	H	H	A	H	A	A	A	B	B	B	B	B	B	B	B	B	B	B	B	B	B	B
5882	JHI-Hv50k-2016-141465	2H	754624512	A	B	H	H	H	H	H	H	H	A	A	H	H	A	H	A	A	A	B	B	B	B	B	B	B	B	B	B	B	B	B	B	B
5887	JHI-Hv50k-2016-141499	2H	754636927	A	B	-	-	H	H	H	H	H	-	A	H	H	A	H	A	A	A	B	B	B	B	B	B	B	B	B	B	B	B	B	B	B
5894	JHI-Hv50k-2016-141647	2H	754713223	A	B	H	H	H	H	H	H	H	A	A	H	H	A	H	A	A	A	B	B	B	B	B	B	B	B	B	B	B	B	B	B	B
5900	JHI-Hv50k-2016-141703	2H	754786562	A	B	H	H	H	H	H	H	H	A	A	H	H	A	H	A	A	A	B	B	B	B	B	B	B	B	B	B	B	B	B	B	B
5910	JHI-Hv50k-2016-141906	2H	755078889	A	B	H	H	H	H	H	H	H	A	A	H	H	A	H	A	A	A	B	B	B	B	B	B	B	B	B	B	B	B	B	B	B
5918	JHI-Hv50k-2016-141981	2H	755181466	A	B	H	H	H	H	H	H	H	A	A	H	H	A	H	A	A	A	B	B	B	B	B	B	B	B	B	B	B	B	B	B	B
5921	JHI-Hv50k-2016-142033	2H	755197171	A	B	H	H	H	H	H	H	H	A	A	H	H	A	H	A	A	A	B	B	B	B	B	B	B	B	B	B	B	B	B	B	B
5923	JHI-Hv50k-2016-142044	2H	755314635	A	B	H	H	H	H	H	H	H	A	A	H	H	A	H	A	A	A	B	B	B	B	B	B	B	B	B	B	B	B	B	B	B
5924	JHI-Hv50k-2016-142048	2H	755314916	A	B	H	H	H	H	H	H	H	A	A	H	H	A	H	A	A	A	B	B	B	B	B	B	B	B	B	B	B	B	B	B	B
5928	JHI-Hv50k-2016-142124	2H	755729724	A	B	H	H	H	H	H	H	H	A	A	H	H	A	H	A	A	A	B	B	B	B	B	B	B	B	B	B	B	B	B	B	B
5963	JHI-Hv50k-2016-142540	2H	757114013	A	B	H	H	H	H	H	H	B	A	A	H	H	A	H	A	A	A	B	B	B	B	B	B	B	B	B	B	B	B	B	B	B
5970	JHI-Hv50k-2016-142592	2H	757213746	A	B	H	H	H	H	H	H	B	A	A	H	H	A	H	A	A	A	B	B	B	B	B	B	B	B	B	B	B	B	B	B	B
5988	JHI-Hv50k-2016-142653	2H	757383882	A	B	H	H	H	H	H	H	B	A	A	H	H	A	H	A	A	A	B	B	B	B	B	B	B	B	B	B	B	B	B	B	B
5990	JHI-Hv50k-2016-142660	2H	757384884	A	B	H	H	H	H	H	H	B	A	A	H	H	A	H	A	A	A	B	B	B	B	B	B	B	B	B	B	B	B	B	B	B
5993	JHI-Hv50k-2016-142663	2H	757385356	A	B	H	H	H	H	H	H	B	A	A	H	H	A	H	A	A	A	B	B	B	B	B	B	B	B	B	B	B	B	B	B	B
5996	JHI-Hv50k-2016-142682	2H	757386100	A	B	H	H	H	H	H	H	B	A	A	H	H	A	H	A	A	A	B	B	B	B	B	B	B	B	B	B	B	B	B	B	B
6001	JHI-Hv50k-2016-142733	2H	757667023	A	B	H	H	H	H	H	H	B	A	A	H	H	A	H	A	A	A	B	B	B	B	B	B	H	B	B	B	B	B	B	B	B
6005	JHI-Hv50k-2016-142802	2H	757746012	A	B	H	H	H	H	H	H	B	A	A	H	H	A	H	A	A	A	B	B	B	B	B	B	H	B	B	B	B	B	B	B	B
6008	JHI-Hv50k-2016-142805	2H	757746561	A	B	H	H	H	H	H	H	-	A	A	H	H	A	H	A	A	A	B	B	B	B	B	B	H	B	B	B	B	B	B	B	B
6009	JHI-Hv50k-2016-142806	2H	757746737	A	B	H	H	H	H	-	H	B	A	A	H	H	A	H	A	A	A	B	B	B	B	B	B	-	B	B	B	B	B	B	B	B
6010	JHI-Hv50k-2016-142842	2H	757819416	A	B	H	H	H	H	H	H	B	A	A	H	H	A	H	A	A	A	B	B	B	B	B	B	H	B	B	B	B	B	B	B	B
6027	JHI-Hv50k-2016-143134	2H	758368903	A	B	H	H	H	H	H	H	B	A	A	H	H	A	H	A	A	A	B	B	B	B	B	B	H	B	B	B	B	B	B	B	B
6028	JHI-Hv50k-2016-143149	2H	758369280	A	B	H	H	H	H	H	H	B	A	A	H	H	A	H	A	A	A	B	B	B	B	B	B	H	B	B	B	B	B	B	B	B
6030	JHI-Hv50k-2016-143180	2H	758384851	A	B	H	H	H	H	H	H	B	A	A	H	H	A	H	A	A	A	B	B	B	B	B	B	H	B	B	B	B	B	B	B	B
6036	JHI-Hv50k-2016-143262	2H	758401785	A	B	H	H	H	H	H	H	B	A	A	H	H	A	H	A	A	A	B	B	B	B	B	B	H	B	B	B	B	B	B	B	B
6040	JHI-Hv50k-2016-143352	2H	758407279	A	B	H	H	H	H	H	H	B	A	A	H	H	A	H	A	A	A	B	B	B	B	B	B	H	B	B	B	B	B	B	B	B
6046	JHI-Hv50k-2016-143440	2H	758556234	A	B	H	H	H	H	H	H	B	A	A	H	H	A	H	A	A	A	B	B	B	B	B	B	H	B	B	B	B	B	B	B	B
6048	JHI-Hv50k-2016-143482	2H	758585058	A	B	A	A	H	H	H	H	-	A	A	H	H	A	H	A	A	A	B	B	B	B	B	B	H	B	B	B	B	B	B	B	B
6051	JHI-Hv50k-2016-143532	2H	758651974	A	B	H	H	H	H	H	H	B	A	A	H	H	A	H	A	A	A	B	B	B	B	B	B	H	B	B	B	B	B	B	B	B
6059	JHI-Hv50k-2016-143636	2H	758717272	A	B	H	H	H	H	H	H	B	A	A	H	H	A	H	A	A	A	B	B	B	B	B	B	H	B	B	B	B	B	B	B	B
6062	JHI-Hv50k-2016-143641	2H	758717741	A	B	H	H	H	H	H	H	B	A	A	H	H	A	H	A	A	A	B	B	B	B	B	B	H	B	B	B	B	B	B	B	B

5812271

6068	JHI-Hv50k-2016-143740	2H	758850273	A	B	H	H	H	H	H	H	B	A	A	H	H	A	H	A	A	A	B	B	B	B	B	B	H	B	B	B	B	B	B	B	B
6071	JHI-Hv50k-2016-143807	2H	759011046	A	B	H	H	H	H	H	H	B	A	A	H	H	A	H	A	A	A	B	B	B	B	B	B	H	B	B	B	B	B	B	B	B
6079	JHI-Hv50k-2016-143888	2H	759146844	A	B	H	H	H	H	H	H	B	A	A	H	H	A	H	A	A	A	B	B	B	B	B	B	H	B	B	B	B	B	B	B	B
6081	JHI-Hv50k-2016-143890	2H	759147805	A	B	H	H	H	H	H	H	B	A	A	H	H	A	H	A	A	A	B	B	B	B	B	B	H	B	B	B	B	B	B	B	B
6082	JHI-Hv50k-2016-143904	2H	759162859	A	B	H	H	H	H	H	H	B	A	A	H	H	A	H	A	A	A	B	B	B	B	B	B	H	B	B	B	B	B	B	B	B
6086	JHI-Hv50k-2016-143975	2H	759281895	A	B	H	H	H	H	H	H	B	A	A	H	H	A	H	A	A	A	B	B	B	B	B	B	H	B	B	B	H	B	B	B	B
6088	JHI-Hv50k-2016-144034	2H	759353476	A	B	H	H	H	H	H	H	B	A	A	H	H	A	H	A	A	A	B	B	B	B	B	B	H	B	B	B	H	B	B	B	B
6091	JHI-Hv50k-2016-144078	2H	759410855	A	B	H	H	H	H	H	H	B	A	A	H	H	A	H	A	A	A	B	B	B	B	B	B	H	B	B	B	H	B	B	B	B
6092	JHI-Hv50k-2016-144152	2H	759744360	A	B	H	H	H	H	H	H	B	A	A	H	H	A	H	A	A	A	B	B	B	B	B	B	H	B	B	B	H	B	B	B	B
6095	JHI-Hv50k-2016-144232	2H	759828120	A	B	H	H	H	H	H	H	B	A	A	H	H	A	H	A	A	A	B	B	B	B	B	B	H	B	B	B	H	B	B	B	B
6096	JHI-Hv50k-2016-144285	2H	759833421	A	B	H	H	H	H	H	H	B	A	A	H	H	A	H	A	A	A	B	B	B	B	B	B	H	B	B	B	H	B	B	B	B
6098	JHI-Hv50k-2016-144336	2H	760023947	A	B	H	H	H	H	H	H	B	A	A	H	H	A	H	A	A	A	B	B	B	B	B	B	H	B	B	B	H	B	B	B	B
6099	JHI-Hv50k-2016-144340	2H	760024020	A	B	H	H	H	H	H	H	B	A	A	H	H	A	H	A	A	A	B	B	B	B	B	B	H	B	B	B	H	B	B	B	B
6100	JHI-Hv50k-2016-144342	2H	760024173	A	B	H	H	H	H	H	H	B	A	A	H	H	A	H	A	A	A	B	B	B	B	B	B	H	B	B	B	H	B	B	B	B
6102	JHI-Hv50k-2016-144507	2H	760139195	A	B	H	H	H	H	H	H	B	A	A	H	H	A	H	A	A	A	B	B	B	B	B	B	H	B	B	B	H	B	B	B	B
6104	JHI-Hv50k-2016-144573	2H	760184175	A	B	H	H	H	H	H	H	B	A	A	H	H	A	H	A	A	A	B	B	B	B	B	B	H	B	B	B	H	B	B	B	B
6108	JHI-Hv50k-2016-144755	2H	760725412	A	B	H	H	H	H	H	H	B	H	A	H	H	A	H	A	A	A	B	B	B	B	B	B	H	B	B	B	H	B	B	B	H
6112	JHI-Hv50k-2016-144805	2H	760752999	A	B	H	H	H	H	H	H	H	-	A	H	H	A	H	A	A	A	B	B	B	B	B	B	H	B	B	B	H	B	B	B	H
6114	JHI-Hv50k-2016-144813	2H	760753473	A	B	H	H	H	H	H	H	B	H	A	H	H	A	H	A	A	A	B	B	B	B	B	B	H	B	B	B	H	B	B	B	H
6117	JHI-Hv50k-2016-144820	2H	760762651	A	B	H	H	H	H	H	H	-	H	A	H	H	A	H	A	A	A	B	B	B	B	B	B	H	B	B	B	H	B	B	B	H
6125	JHI-Hv50k-2016-144957	2H	760935265	A	B	H	H	H	H	H	H	B	H	A	H	H	A	H	A	A	A	B	B	B	B	B	B	H	B	B	B	H	B	B	B	H
6129	JHI-Hv50k-2016-145116	2H	761107172	A	B	H	H	H	H	H	H	B	H	A	H	H	A	H	A	A	A	B	B	B	B	B	B	H	B	B	B	H	B	B	B	H
6130	JHI-Hv50k-2016-145127	2H	761107447	A	B	H	H	H	H	H	H	B	H	A	H	H	A	H	A	A	A	B	B	B	B	B	B	H	B	B	B	H	B	B	B	H
6132	JHI-Hv50k-2016-145133	2H	761107635	A	B	H	H	H	H	H	H	B	H	A	H	H	A	H	A	A	A	B	B	B	B	B	B	H	B	B	B	H	B	B	B	H
6134	JHI-Hv50k-2016-145354	2H	761264461	A	B	H	H	H	H	H	H	B	H	A	H	H	A	H	A	A	A	B	B	B	B	B	B	H	B	B	B	H	B	B	B	H
6136	JHI-Hv50k-2016-145476	2H	761299191	A	B	H	-	H	H	H	H	B	H	A	H	H	A	H	A	A	A	B	B	B	B	B	B	H	B	B	B	H	B	B	B	H
6143	JHI-Hv50k-2016-145572	2H	761327647	A	B	H	H	H	H	H	H	B	H	A	H	H	A	H	A	A	A	B	B	B	B	B	B	H	B	B	B	H	B	B	B	H
6154	JHI-Hv50k-2016-145709	2H	761623717	A	B	H	H	H	H	H	H	B	H	A	H	H	A	H	A	A	A	B	B	B	B	B	B	H	B	B	B	H	B	B	B	H
6167	JHI-Hv50k-2016-146031	2H	762527829	A	B	H	H	H	H	H	A	B	H	A	H	H	A	H	A	A	A	B	H	B	B	B	B	H	B	B	B	H	B	B	B	H
6168	JHI-Hv50k-2016-146053	2H	762651967	A	B	H	H	H	H	H	A	B	H	A	H	H	A	H	A	A	A	B	H	B	B	B	B	H	B	B	B	H	B	B	B	H
6169	JHI-Hv50k-2016-146123	2H	762708157	A	B	H	-	H	H	H	A	B	H	A	H	H	A	-	A	A	A	B	H	B	B	B	B	H	B	B	B	H	B	B	B	H
6179	JHI-Hv50k-2016-146318	2H	762989792	A	B	H	H	H	H	H	A	B	H	A	H	H	A	H	A	A	A	B	H	B	B	B	B	H	B	B	B	H	B	B	B	H
6181	JHI-Hv50k-2016-146344	2H	762992556	A	B	H	H	H	H	H	A	B	H	A	H	H	A	H	A	A	A	B	H	B	B	B	B	H	B	B	B	H	B	B	B	-
6187	JHI-Hv50k-2016-146702	2H	763551936	A	B	H	H	H	H	H	A	B	H	A	H	H	A	H	A	A	A	B	H	B	B	B	B	H	B	B	B	H	B	B	B	H
6189	JHI-Hv50k-2016-146783	2H	763961505	A	B	H	H	H	H	H	A	B	H	A	H	H	A	H	A	A	A	B	H	B	B	B	B	H	B	B	B	H	B	B	B	H
6197	JHI-Hv50k-2016-146921	2H	764279786	A	B	H	H	H	H	H	A	B	H	A	H	H	A	H	A	A	A	B	H	B	B	B	B	H	B	B	B	H	B	B	B	H
6202	JHI-Hv50k-2016-147075	2H	764420583	A	B	H	H	H	H	H	A	B	H	A	H	H	A	H	A	A	A	B	H	B	B	B	B	H	B	B	B	H	B	B	B	H
6203	JHI-Hv50k-2016-147080	2H	764421012	A	B	A	A	H	H	H	A	B	A	A	H	H	A	H	A	A	A	B	H	B	B	B	B	H	B	B	B	H	B	B	B	H
6204	JHI-Hv50k-2016-147100	2H	764424264	A	B	H	H	H	H	H	A	B	H	A	H	H	A	H	A	A	A	B	H	B	B	B	B	H	B	B	B	H	B	B	B	H
6205	JHI-Hv50k-2016-147120	2H	764591208	A	B	H	H	H	H	H	A	B	H	A	H	H	A	H	A	A	A	B	H	B	B	B	B	H	B	B	B	H	B	B	B	H
6206	JHI-Hv50k-2016-147130	2H	765154930	A	B	H	H	H	H	H	A	B	H	A	H	H	A	H	A	A	A	B	H	B	B	B	B	H	B	B	B	H	B	B	B	H
6207	JHI-Hv50k-2016-147162	2H	765158515	A	B	H	H	H	H	H	A	B	H	A	H	H	A	H	A	A	A	B	H	B	B	B	B	H	B	B	B	H	B	B	B	H
6208	JHI-Hv50k-2016-147165	2H	765158629	A	B	H	H	H	H	H	A	B	H	A	H	H	A	H	A	A	A	B	H	B	B	B	B	H	B	B	B	H	B	B	B	H
6211	JHI-Hv50k-2016-147190	2H	765606488	A	B	B	B	H	H	H	A	B	B	A	H	H	A	H	A	A	A	B	H	B	B	B	B	H	B	B	B	H	B	B	B	H
6212	JHI-Hv50k-2016-147195	2H	765607602	A	B	H	H	H	H	H	A	B	H	A	H	H	A	H	A	A	A	B	H	B	B	B	B	H	B	B	B	H	B	B	B	H
6213	JHI-Hv50k-2016-147197	2H	765607788	A	B	H	H	H	H	H	A	B	H	A	H	H	A	H	A	A	A	B	H	B	B	B	B	H	B	B	B	H	B	B	B	H
6214	JHI-Hv50k-2016-147198	2H	765607875	A	B	H	H	H	H	H	A	B	H	A	H	H	A	H	A	A	A	B	H	B	B	B	B	H	B	B	B	H	B	B	B	H
6215	JHI-Hv50k-2016-147217	2H	765618608	A	B	H	H	H	H	H	A	B	H	A	H	H	A	H	A	A	A	B	H	B	B	B	B	H	B	B	B	H	B	B	B	H
6218	JHI-Hv50k-2016-147265	2H	765631993	A	B	H	H	H	H	H	A	B	H	A	H	H	A	H	A	A	A	B	H	B	B	B	B	H	B	B	B	H	B	B	B	H
6219	JHI-Hv50k-2016-147293	2H	765637478	A	B	H	H	H	H	H	A	B	H	A	H	H	A	H	A	A	A	B	H	B	B	B	B	H	B	B	B	H	B	B	B	H
6223	JHI-Hv50k-2016-147337	2H	765722661	A	B	H	H	H	H	H	A	B	H	A	H	H	A	H	A	A	A	B	H	B	B	B	B	H	B	B	B	H	B	B	B	H
6225	JHI-Hv50k-2016-147392	2H	765754276	A	B	H	H	H	H	H	A	B	H	A	H	H	A	H	A	A	A	B	H	B	B	B	B	H	B	B	B	H	B	B	B	H
6226	JHI-Hv50k-2016-147394	2H	765754346	A	B	H	H	H	H	H	A	B	H	A	H	H	A	H	A	A	A	B	H	B	B	B	B	H	B	B	B	H	B	B	B	H
6227	JHI-Hv50k-2016-147420	2H	765910664	A	B	H	H	H	H	H	A	B	H	A	H	H	A	H	A	A	A	B	H	B	B	B	B	H	B	B	B	H	B	B	B	H
6234	JHI-Hv50k-2016-147621	2H	766081896	A	B	H	H	H	H	H	A	B	H	A	H	H	A	H	A	A	A	B	H	B	B	B	B	H	B	B	B	H	B	B	B	H
6238	JHI-Hv50k-2016-147709	2H	766101130	A	B	H	H	H	H	H	A	B	H	A	H	H	A	H	A	A	A	B	H	B	B	B	B	H	B	B	B	H	B	B	B	H
6246	JHI-Hv50k-2016-147863	2H	766950959	A	B	H	H	H	H	H	A	B	H	A	H	H	A	H	A	A	A	B	H	B	B	B	B	H	B	B	B	H	B	B	B	H
6254	JHI-Hv50k-2016-147952	2H	767057095	A	B	A	A	H	H	H	A	H	A	A	H	H	A	H	A	A	A	B	H	B	B	B	B	H	B	B	B	H	B	B	B	H
6255	JHI-Hv50k-2016-147953	2H	767057612	A	B	H	H	H	H	H	A	B	H	A	H	H	A	H	A	A	A	B	H	B	B	B	B	H	B	B	B	H	B	B	B	H

Additional Table 2. Primers used for the analysis of HvPTOX alleles.

Primer name	Sequence
F1	CCAGCTCCAGAGGTGGCTGT
F2	CGCTCCCGACGACAAAACA
F3	TGTCAGGCGTTGGGTGGCAA
F4	TTTCGAATAACGCCGCCTCT
F5	GTAGGCTCGGCATGAGACAA
R1	CAGCGCTCTAGCACGGAGGT
R2	TGTTTTGTCGTCGGGAGCG
R3	CATATGCCTGGTGCTACCGA
